# An analysis of the top 500 anesthesiology publications with the highest altmetric attention scores

**DOI:** 10.1097/MD.0000000000041523

**Published:** 2025-02-21

**Authors:** Dilek Ömür Arça, Başak Bayram, Nilay Boztaş, İsmail Erdemir, Murat Çetin, Gönül Sağiroğlu, Volkan Hanci

**Affiliations:** a Department of Anesthesiology and Reanimation, Faculty of Medicine, Dokuz Eylül University, Izmir, Turkey; b Department of Emergency Medicine, Chief Physician Izmir Metropolitan Manucipality Eşrefpaşa Hospital, Izmir, Turkey; c Department of Anesthesiology and Reanimation, Faculty of Medicine, Dokuz Eylül University, Izmir, Turkey; d Department of Emergency Medicine, Ministry of Health Dr. Behçet Uz Pediatrics and Surgery Training and Research Hospital, Izmir, Turkey; e Department of Anesthesiology and Reanimation, Faculty of Medicine, Dokuz Eylül University, Izmir, Turkey.

**Keywords:** Altmetric attention score, anesthesiology, citation rate

## Abstract

Altmetrics is a web-based measurement method that assesses the online dissemination and interactions of an article. We performed an altmetric analysis of 500 papers with the highest altmetric attention score (AAS) published in anesthesiology-related journals. Journals were identified from the Web of Science (WoS) Master Journal List by Clarivate using the category “Anesthesiology.” The altmetric data were obtained from the Altmetric Explorer database. The first 500 publications with the highest AAS scores among these journals were identified and analyzed. Using Spearman correlation, no correlation was observed between the AAS and either WoS or Google Scholar citations (*R* = 0.188, *P* < .001 and *R* = 0.161, *P* < .001, respectively). There was a weak correlation between blog mentions and both WoS citations and Google Scholar citations (*R* = 0.263, *P* < .001 and (*R* = 0.241, *P* < .001). A very strong correlation was observed between the number of Mendeley readers and both WoS and Google Scholar citations (*R* = 0.889, *P* < .001 and *R* = 0.905, *P* < .001). A significant difference in AAS and WoS citations was observed based on publication topic (*P* = .036 and *P* = .005, respectively), with algology being the most common topic (n = 206, 41.2%). Although AAS did not significantly affect traditional scientific citations, the analysis of subgroup correlations revealed notable differences. Our results suggest that traditional scientific citations (WoS and Google Scholar citations) are strongly influenced by the number of Mendeley readers. Further research is needed to understand these dynamics in academic discourse.

## 1. Introduction

The main purpose of scientific publishing is to share scientific findings, ideas, and discussions globally and within the academic community in order to drive scientific advancement.^[1,2]^ The widespread use of various online platforms has greatly enhanced the accessibility of scientific findings.^[1]^ In scientific publishing, the impact of articles is often assessed through various metrics, such as the number of citations and the impact factor (IF) of the journal in which they are published.^[2-6]^ Citation analysis, one of the most widely used bibliometric methods, can indicate an article’s credibility through frequent citations, thus supporting the researcher’s arguments.^[7-10]^ However, accumulating citations takes time, and delays of several years may occur in some fields.^[1,11,12]^ During this period, an article may lose its topicality or the attention of the relevant reader may be diverted from the subject of the article.^[1,13]^ As a result, the impact of this study may be diminished over time.^[1]^

With the increasing popularity of social media, a new concept called “altmetric analysis” has emerged as a result of scientific articles being shared on social media.^[14]^ Altmetrics, first proposed in 2010, tracks the sharing of articles on networks such as Twitter, Facebook, Mendeley, and Instagram, assigning a score known as the altmetric attention score (AAS).^[14,15]^ Unlike traditional citation metrics, the AAS reflects a publication’s potential to spread quickly and reach a wide audience. However, the AAS tends to measure short-term and immediate attention and interaction, which may not be sufficient to assess the long-term scientific impact of a publication. Articles on a popular topic may receive high AAS scores regardless of their scientific merit. The AAS cannot measure the scientific quality of a publication or the accuracy of its content. Popular topics or controversial studies may attract more attention, skewing the AAS.^[16]^

In addition to traditional bibliometric studies in various health fields, numerous studies have compared metric and bibliometric analyses.^[1,9,10,14]^ Despite the intense interest in altmetrics within the academic community, the relationship between AAS and traditional citation counts remains ambiguous. Previous studies in health sciences have generally reported a weak correlation between citation counts and AAS.^[15-19]^ However, there are also publications that report a positive correlation.^[20]^ There are a limited number of altmetric studies in anesthesiology literature.^[17,19,21]^

This study aimed to analyze 500 articles with the highest AAS scores in anesthesiology journals and evaluate the relationship between these scores and traditional citation counts. Our hypothesis posits that articles with higher AAS scores have higher citation counts. Through a meticulous analysis of these papers, our approach seeks to provide valuable insights into the evolving landscape of scientific communication.

## 2. Materials and methods

### 2.1. Study design

Because the databases used in this study were public, ethics committee approval was not required.

#### 2.1.1. Inclusion criteria

The top 500 publications with the highest AAS among anesthesiology journals indexed in the Web of Science (WoS) Master Journal List (MJL) by Clarivate.

#### 2.1.2. Exclusion criteria

Not among the top 500 publications with the highest AAS among anesthesiology journals and not indexed by the MJL.

### 2.2. Search strategy population

#### 2.2.1. Data extraction

The first step was to identify the journals to be searched to determine the journal/collection title. Therefore, the journals included in the study were identified by searching the MJL at Clarivate (https://mjl.clarivate.com/search-results) using the category “Anesthesiology.” The 64 journals obtained were included in this study (listed alphabetically in Appendix 1, Supplemental Digital Content, http://links.lww.com/MD/O373). The research data supporting this publication are available at https://archive.org/embed/appendix-3-the-top-500-aas-15112024.

#### 2.2.2. Searching the altmetric system

In the second step, the Altmetric database (Altmetric Explorer, Altmetric LLP, London, UK; https://www.altmetric.com) was used for March 20, 2024. The types of output “articles,” type of Open Access (OA), and “all outputs” were selected from the Altmetric database. The ISSN numbers of all journals included in the study were entered, and all publications were scanned without time limitations. All articles in these journals, including altmetrics data, were downloaded to Microsoft Excel (Microsoft Corporation, New York). Articles with the highest AAS score (500) were included in the study.

#### 2.2.3. Data classification

The collected data included AAS scores, title, authors, year of publication, journal name, institutional affiliation, article type, article subject, and number of mentions on online platforms (such as blogs, Twitter, Facebook, and Wikipedia). In the next step, to gather these details, access was obtained to either the abstract or full text of each publication to ensure accuracy and comprehensiveness. In addition, to create a citation-based metric for all evaluated publications, citation counts were recorded using WoSCiteScore (Clarivate, London, United Kingdom) and Google Scholar (Google LLC, California) databases. Furthermore, the IF, H-index, and quartiles of the journals were retrieved from the “SCImago Journal & Country Rank” (https://www.scimagojr.com/) for quartile classification.

The 500 publications included in this study were categorized according to topic. Based on their subject matter, these publications were classified into the following categories: algology, anesthesia practice, coronavirus disease 2019 (COVID-19), airway management, monitoring, healthcare worker safety, patient safety, regional anesthesia, intensive care, trauma, and others. Given the methodologies used in the publications, some were relevant to multiple topics. In such cases, each article was independently re-evaluated by 3 researchers, and the topic was assigned based on its primary purpose (i.e., the expected outcome of the study). Decisions were made unanimously.

For classification by publication type, the abstracts were initially reviewed. If the abstract did not provide sufficient information regarding the study design or type, the full text of the article was examined. The study type was approved by a consensus among the 3 researchers. These publications were classified according to type as follows: reviews, randomized clinical trials, prospective observational studies, retrospective observational studies, editorials, guidelines, surveys, case reports, and experimental studies. The institutional affiliation of the first author was used to determine the geographical origin of each publication. Average citations per year are calculated by dividing the total number of citations a paper has received by the number of years since its publication (average citation per year = total citation count/number of years since publication). All these data were evaluated to analyze the relationship between AAS and citation counts of the publications.

#### 2.2.4. Statistical analysis

Statistical analyses were performed using IBM SPSS Statistics version 29.0 (New York) for Windows and Jamovi, version 2.3.28 (SEM 0.9.5 package, New York). Categorical variables are described as numbers and percentages. The distribution of continuous variables was analyzed using the Kolmogorov–Smirnov test. Depending on the distribution of the variables, they were described as mean ± standard deviation or median and interquartile range (IQR). The Mann–Whitney *U* and Kruskal–Wallis tests were used to compare groups. Spearman correlation test was used to analyze the correlation of the AAS with the sources and traditional metrics. A *P* value of <.05 was determined as a significant difference.

## 3. Results

**Figure 1.**
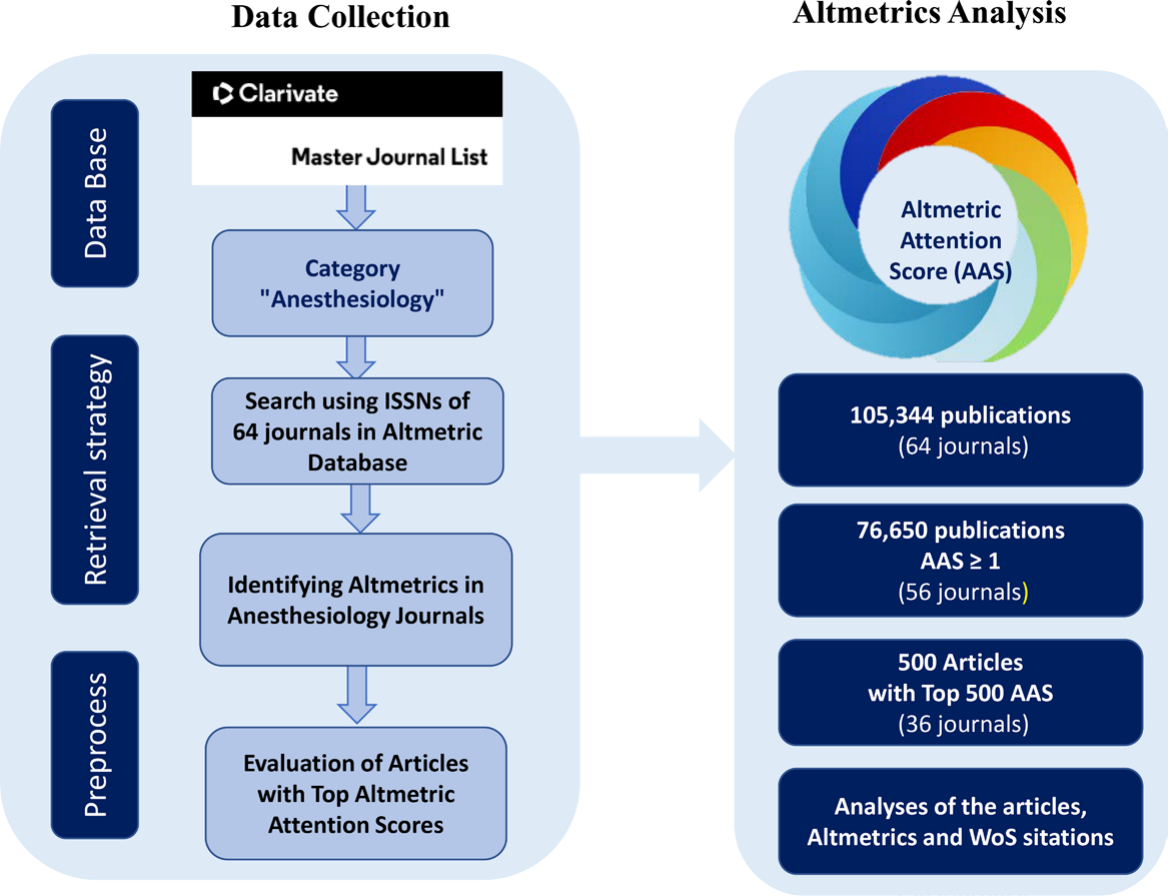
The flow chart of data analysis process. ISSN = International Standard Serial Number, WoS = Web of Science.

A total of 105.344 publications from these journals were identified, with a median AAS of 2 (IQR: 0–4). Of these, 76.650 publications had an AAS ≥ 1, and 3 publications were unavailable for the AAS assessment. The median AAS of the 76.647 assessable publications was 3 (IQR: 1–7), with the AAS of these papers ranging from 1 to 2731. Analysis of the data revealed that 76.649 publications originated in 56 journals. The top 500 publications with the highest AAS were identified and analyzed. One datum from the top 500 rankings was unavailable and excluded from the analysis; the next highest AAS was included to maintain a sample size of 500. Among the 500 articles analyzed, 1 was in German, 2 were in Spanish, and the remaining were in English. Because English abstracts were available for all 3 non-English articles, they were included in this study (Fig. 1). Despite our efforts to retrieve WoS values using digital object identifier numbers, we were unable to locate the corresponding data for 9 specific publications (2 posters and 1 infographic). The first 500 publications with the highest AAS from the included journals, along with their respective altmetric data, are detailed in Appendices 2 and 3, Supplemental Digital Content, http://links.lww.com/MD/O373, http://links.lww.com/MD/O373 (available at https://archive.org/embed/appendix-3-the-top-500-aas-15112024).

### 3.1. Analyzing of the altmetrics

The AAS of the top 500 papers ranged from 164 to 2731. The median AAS of the most cited papers was 281.5 (IQR: 211.0–417.5). For mentions from policy documents, patents, Wikipedia, Google, LinkedIn, Reddit, Pinterest, F100, Q/A, and videos, the median was 0.0 (IQR: 0.0–0.0). Additional sources and their respective metrics are listed in Table 1. The median WoS citations of these articles were 40.0 (IQR: 13.0–121.0) and Google Scholar citations were 75.0 (IQR: 23.0–215.5). The WoS citations per year for these articles was 6.6 (IQR: 2.0–16.9), while the Google Scholar citations per year was 11.9 (IQR: 3.8–31.7). The analysis revealed a weak correlation between AAS and WoS citations per year (*R* = 0.174, *P* < .001). In addition, there was an even weaker correlation between the AAS and Google Scholar citations (*R* = 0.142, *P* < .001).

**Table 1:** General descriptions of most mentioned 500 papers.

Source	Median (25%–75%))
AAS	280 (211**–**418)
Subgroups of AAS	
News mentions	27.0 (4.0**–**48.3
Blog mentions	1.0 (0.0**–**3.0)
X mentions	128.0 (14.0**–**381.0)
Facebook mentions	1.0 (0.0**–**5.0)
Number of the Mendeley readers	109.0 (39.0**–**228)
Number of the dimension citations	50.0 (16.0**–**227)
WoS citations	40.0 (13.0**–**121.0)
Google Scholar citations	75.0 (23.0**–**215.5)

AAS = altmetric attention score, WoS = Web of Science.

**Figure 2.**
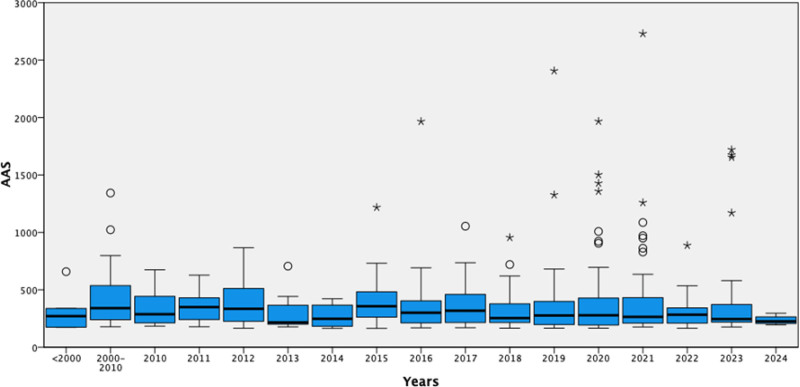
The figure illustrates the median AAS, of the articles across different years. AAS = altmetric attention score

The articles included in this study were published between 1985 and 2024. The highest median AAS was observed in 2015, with a value of 356.5 (IQR: 258.8–488.8). The second-highest median AAS was recorded between 2000 and 2010 (Fig. 2). For analysis purposes, the years were grouped into 3 categories: pre-2000, 2000-2010, and post-2010.

The article with the highest AAS of 2731 was titled “Timing of surgery following SARS-CoV-2 infection: an international prospective cohort study,” authored by the COVIDSurg Collaborative; GlobalSurg Collaborative, and published in the journal *Anesthesia* in 2021. Subsequent articles covered a diverse range of topics without discernible patterns. Among the first authors, Cook TM ranked 1st with 5 publications.

**Figure 3.**
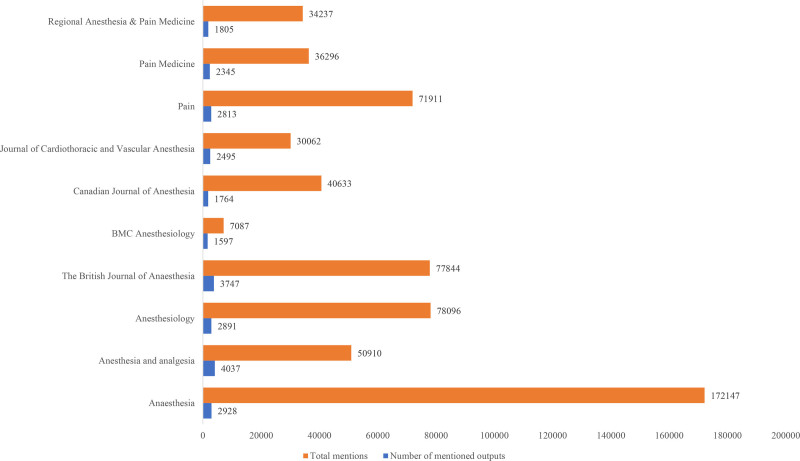
Total mentions and the number of mentioned outputs of the journal.

A total of 37 journals were included in the 500 top AAS list (Table 2). The IF, H-index, and quartiles of these journals were analyzed. The mean IF was 28.16 ± 25.58 (ranging from 2.00 to 107.00), and the mean H-index was 84.78 ± 72.21 (ranging from 1.00 to 296). The journal with the highest IF was “Anaesthesia” (IF: 10.7), and the journal with the highest H-index was “Pain” (H-index: 296). According to the SCImago Journal & Country Rank, there were 12 journals in the Q1 category, 13 journals in the Q2 category, 9 journals in the Q3 category, and 3 journals in the Q4 category (Table 2). In addition, 51.4% (n = 257) of these 500 publications appeared in only 3 journals. “Anaesthesia” accounted for 107 publications (21.4%), “Pain” for 93 publications (18.6%), and “Anesthesiology” for 56 publications (11.2%) (Table 3). The top 10 journals with the highest total number of mentions and publications are illustrated in Figure 3.

**Table 2:** Breakdown of the 500 top altmetric attention score papers by journal-wise distribution.

Journals and/collections	Journal ISSNs	Total papers	Impact factor	Quartile	H-index
*Acta Anaesthesiologica Scandinavica*	0001-5172, 1399-6576	1	1.9	Q2	122
*Ain-Shams Journal of Anesthesiology*	1687-7934, 2090-925X	1	0.5	Q4	8
*Anaesthesia*	0003-2409, 1365-2044	107	10.7	Q1	141
*Anaesthesia and Intensive Care*	0310-057X, 1448-0271	3	1.1	Q3	72
*Anaesthesia and Intensive Care Medicine*	1472-0299	1	0.2	Q4	19
*Anaesthesia Reports*	2637-3726	2	0.8	Q3	6
*Anesthesia and Analgesia*	0003-2999, 1526-7598	36	4.6	Q1	227
*Anesthesiology*	0003-3022, 1528-1175	56	9.1	Q1	267
*Anesthesiology Research & Practice*	1687-6962, 1687-6970	1	1.6	Q2	28
*Baillière’s Clinical Anaesthesiology, Best Practice & Research Clinical Anaesthesiology*	1521-6896, 1878-1608	3	4.7	Q1	82
*BJA: The British Journal of Anaesthesia*	1471-6771, 0007-0912	31	9.1	Q1	212
*BMC Anesthesiology*	1471-2253	3	2.3	Q2	54
*Canadian Journal of Anesthesia*	0832-610X, 1496-8975	11	3.4	Q1	113
*Clinical Journal of Pain*	0749-8047, 1536-5409	16	2.6	Q2	145
*Current Anesthesiology Reports*	2167-6275	2	1.6	Q2	20
*Der Schmerz*	0932-433X, 1432-2129	1	1.1	Q3	46
*European Journal of Pain*	1090-3801, 1532-2149	17	3.5	Q1	129
*International Anesthesiology Clinics*	0020-5907, 1537-1913	1	0.8	Q3	36
*Journal of Anesthesia*	0913-8668, 1438-8359	1	2.8	Q2	58
*Journal of Cardiothoracic and Vascular Anesthesia*	1053-0770, 1532-8422	10	2.3	Q2	96
*Journal of Clinical Anesthesia*	0952-8180, 1873-4529	6	5.0	Q1	81
*Journal of Clinical Monitoring and Computing*	1387-1307, 1573-2614	5	2	Q2	61
*Journal of Pain & Palliative Care Pharmacotherapy*	1536-0288, 1536-0539	1	0.9	Q3	45
*Korean Journal of Anesthesiology*	2005-6419, 2005-7563	1	4.2	Q1	44
*Local and Regional Anesthesia*	1178-7112	1	1.5	Q3	26
*Minerva Anestesiologica*	0375-9393, 1827-1596	2	2.9	Q2	70
*Paediatric Anaesthesia*	1155-5645, 1460-9592	3	1.7	Q2	98
*Pain (03043959*)	0304-3959, 1872-6623	93	5.9	Q1	296
*Pain Medicine*	1526-2375, 1526-4637	46	2.9	Q1	121
*Pain Physician*	1533-3159, 2150-1149	5	2.6	Q2	114
*Pain Practice*	1530-7085, 1533-2500	4	2.5	Q2	73
*Perioperative Medicine*	2047-0525	2	2	Q2	21
*Regional Anesthesia & Pain Medicine*	1098-7339, 1532-8651	18	5.1	Q1	124
*Revista Española de Anestesiología y Reanimación*	0034-9356, 2341-1929	2	0.9	Q3	28
*Saudi Journal of Anaesthesia*	1658-354X, 0975-3125	5	1.3	Q3	36
*Southern African Journal of Anaesthesia and Analgesia*	2220-1173, 2220-1181	1	0.3	Q4	13
*Trends in Anaesthesia and Critical Care*	2210-8440	1	1.4	Q3	25

ISSN = International Standard Serial Number.

**Table 3:** Analysis of the subjects of the papers, addresses, open access status, AAS, and WoS citations.

Specifications	n (%)	AAS Median (25%–75%)	*P*	WoS citations	*P*
Subjects					
Algology	206 (41.2)	282.0 (208.0**–**434.0)	.036*^,^†	60.0 (16.0**–**166.0)	.005*^,^†
Anesthesia practice	126 (25.2)	269.0 (212.0**–**350.0)		37.0 (18.0**–**97.0)	
COVID-19	47 (9.4)	369.0 (224.0**–**632.0)		53.0 (17.0**–**217.0)	
Airway management	24 (4.8)	253.0 (193.0**–**398.5)		19.0 (9.3**–**96.5)	
Monitorization	24 (4.8)	280.0 (222.0**–**403.0)		28.0 (5.0**–**57.0)	
Healthcare workers safety	19 (3.8)	350.0 (234.5**–**532.0)		40.5 (14.3**–**130.8)	
Patient safety	18 (3.6)	287.5 (217.5**–**447.3)		26.0 (8.0**–**64.8)	
Other	18 (3.6)	268.0 (177.5**–**478.5)		27.0 (3.0**–**128.0)	
Regional anesthesia	7 (1.4)	235.5 (180.0**–**548.0)		23.0 (1.0**–**67.0)	
Intensive care	6 (1.2)	234.5 (180.0**–**347.0)		2.5 (0.0**–**22.8)	
Trauma	5 (1.0)	219.0 (173.5**–**270.0)		23.0 (14.5**–**27.5)	
Type of the study					
Review	161 (32.2)	270.0 (206.0**–**396.5)	.005*^,^†	67.0 (22.5**–**213.5)	<.001*^,^†
Randomized clinical trials	84 (16.8)	327.0 (237.5**–**478.5)		46.0 (18.0**–**115.5)	
Prospective observational	75 (15.0)	266.5 (205.8**–**399.0)		39.0 (13.0**–**101.0)	
Retrospective observational	45 (9.0)	300.0 (225.5**–**462.5)		25.0 (9.0**–**70.8)	
Editorial	40 (8.0)	247.0 (191.5**–**430.0)		11.0 (1.0**–**41.3)	
Guideline	36 (7.2)	247.5 (208.3**–**352.0)		78.0 (28.5**–**207.0)	
Survey	29 (5.8)	244.0 (183.0**–**357.3)		24.0 (4.8**–**60.0)	
Case reports	16 (3.2)	351.5 (255.5**–**875.3)		13.0 (3.3**–**57.8)	
Experimental	14 (2.8)	384.5 (268.5**–**917.5)		19.0 (10.5**–**160.8)	
Country					
United States	217 (43.4)	310.0 (218.0**–**459.0)	.034*^,^‡	41.0 (12.3**–**134.5)	.915‡
Non-USA	283 (56.6)	266.0 (179.8**–**266.0)		40.0 (15.0**–**139.0)	
Open access situation					
Yes	421 (84.2)	287.0 (213.5**–**426.0)	.109‡	36.0 (12.5**–**112.5)	.844‡
No	79 (15.8)	259.0 (187.0**–**369.0)		49.0 (13.0**–**141.0)	

AAS = altmetric attention score, COVID-19 = coronavirus disease 2019, WoS = Web of Science.

* *P* < .05 statistically significant.

† Kruskal–Wallis test.

‡ Mann–Whitney *U* test.

To examine the relationship between publication altmetrics and traditional citation counts, additional analyses were conducted to explore the relationships between altmetrics and publication year, country, study type, and subject matter. Of the papers included in the study, 217 (43.4%) were published in the United States, 118 (23.6%) in the United Kingdom, and 33 each in Australia and Canada (6.6% each), encompassing a total of 39 countries (Fig. 3). When analyzed by country, there was a statistically significant difference in AAS citations (*P* = .034) but not in WoS citations (*P* = .915) (Table 3).

Publications were categorized according to the subject. The most common subjects were in algology (n = 206, 41.2%) and anesthesia practice (n = 126, 25.2%). Statistical analysis revealed a significant difference in both AAS and WoS citations across different subjects (*P* = .036 and *P* = .005, respectively) (Table 3). During the analysis of articles for methods, systematic reviews, meta-analyses, and reviews were grouped under a single category (review), while randomized controlled trials and prospective randomized studies were classified as randomized clinical trials. Among the 500 articles with the highest AAS scores, the most common research types were review articles (n = 161, 32.2%), randomized clinical trials (n = 84, 16.8%), and prospective observational studies (n = 69, 13.8%). The highest WoS citations by article type were found in guidelines, reviews, and randomized clinical trials, whereas the highest AAS was observed in experimental studies, case reports, and prospective observational studies (Table 3). Of the publications included in this study, 421 (84.0%) were articlesin OA articles. No statistically significant differences were observed in OA papers and AAS (*P* = .109) or WoS citations (*P* = .446). Detailed analyses of the papers’ subjects, study designs, OA status, AAS, and WoS citations are shown in Table 3.

### 3.2. Correlations of the citations and the altmetrics mentions

This section examines the correlation between scientific citations and altmetrics for the top 500 publications in anesthesiology. The analysis included the relationships between the number of mentions on various online platforms (such as blogs, X, Facebook, and Wikipedia) and citations from WoS and Google Scholar.

Using Spearman correlation, no correlation was observed between AAS and either WoS or Google Scholar citations (*R* = 0.099, *df* = 489, *P* = .028 and *R* = 0.092, *df* = 498, *P* = .040, respectively). However, the situation differed in the subgroup correlations. There was a strong correlation between news mentions and AAS (*R* = 0.748, *df* = 498, *P* < .001). There was a weak correlation between blog mentions and both WoS citations and Google Scholar citations (*R* = 0.263, *df* = 489, *P* < .001 and *R* = 0.241, *df* = 498, *P* < .001). In addition, a very strong correlation was observed between the number of Mendeley readers and both WoS citations and Google Scholar citations (*R* = 0.889, *df* = 489, *P* < .001 and *R* = 0.905, *df* = 498, *P* < .001, respectively). Table 4 presents the correlation matrices.

**Table 4:** Correlations between AAS, sources, and traditional citations from different databases.

	AAS	WOS citations	Google Scholar citations	News mentions	Blog mentions	X mentions	Facebook mentions	Number of Mendeley readers	Number of dimensions citations
AAS									
Pearson *r*	—								
*df*	—								
*P*	—								
WOS citations									
Pearson *r*	0.099	—							
*df*	489	—							
*P*	.028*	—							
Google Scholar citations									
Pearson *r*	0.092	0.987	—						
*df*	498	489	—						
*P*	.040*	<.001*	—						
News mentions									
Pearson *r*	0.748	0.056	0.055	—					
*df*	498	489	498	—					
*P*	<.001*	.217	.218	—					
Blog mentions									
Pearson *r*	0.364	0.263	0.241	0.187	—				
*df*	498	489	498	498	—				
*P*	<.001*	<.001*	<.001*	<.001*	—				
X mentions									
Pearson *r*	0.409	0.026	0.020	-0.122	0.239	—			
*df*	498	489	498	498	498	—			
*P*	<.001*	.563	.656	.006*	<.001*	—			
Facebook mentions									
Pearson *r*	0.065	0.122	0.121	-0.009	0.181	0.152	—		
*df*	498	489	498	498	498	498	—		
*P*	.146	.007*	.007*	.836	<.001*	<.001*	—		
Number of Mendeley readers									
Pearson *r*	0.133	0.889	0.905	0.062	0.247	0.109	0.222	—	
df	498	489	498	498	498	498	498	—	
*P*	.003*	<.001*	<.001*	.168	<.001*	.014*	<.001*	—	
Number of dimensions citations									
Pearson *r*	0.100	0.994	0.989	0.050	0.265	0.037	0.130	0.896	—
*df*	498	489	498	498	498	498	498	498	—
*P*	.026*	<.001*	<.001*	.268	<.001*	.413	.004*	<.001*	—

AAS = altmetric attention score, *r* = Spearman rank correlation coefficient, WoS = Web of Science.

* *P* < .05 statistically significant.

## 4. Discussion

There are a limited number of altmetric studies in anesthesiology journals in the literature.^[19,21-23]^ Our study presents an altmetric analysis of the top 500 publications with the highest AAS among journals publishing in anesthesiology. The results of this study provide a perspective on the impact of the online dissemination of publications in anesthesiology on citation counts.

### 4.1. Main results and conclusions

The emphasis of this study was that no correlation was observed between AAS and citations from either WoS or Google Scholar. However, analysis of the subgroup correlations revealed notable differences. Specifically, there was a strong correlation between news mentions and AAS. In addition, a weak correlation was found between blog mentions and WoS and Google Scholar citations. Furthermore, a strong correlation was observed between the number of Mendeley readers and citations from both WoS and Google Scholar.

### 4.2. Comparison with literature

Based on our study findings, review articles and randomized clinical trials were identified as the most prevalent research types. The highest WoS citations by article type were found in guidelines, reviews, and randomized clinical trials, whereas the highest AAS was observed in experimental studies, case reports, and prospective observational studies. A high WoS citation count typically reflects an article’s quality, importance to ongoing research, and influence on the development of scientific knowledge. This metric is widely used to assess scholarly impact and dissemination of research findings.^[9,10]^

In the literature, some publications report both a weak correlation^[15,16,18]^ and no correlation^[2,10,24]^ between the AAS and citation counts. Grosh et al^[19]^ reviewed the 2-year publications of a single anesthesia journal (*Regional Anesthesia and Pain Medicine Journal*) and analyzed 100 articles with the highest AAS. The authors reported a weak but significant correlation between AAS and the number of citations. On the other hand, there was no correlation between AAS and traditional citation scores in various disciplines, including medical professionalism (*R* = 0.064, *P* = .661),^[10]^ vitreoretinal surgery (*R* = 0.091, *P* = .367),^[2]^ and plastic and reconstructive surgery.^[24]^ Rong et al^[22]^ identified and analyzed 100 most cited articles in 5 anesthesiology journals with the highest Clarivate analytics IF in 2016 and 2018. The authors found different results for both years. They reported a weak correlation between the AAS and citation counts for articles published in 2016 (*R* = 0.40) but found no correlation between the AAS and citation counts for articles published in 2018 (*R* = 0.13). In this study, we found no correlation between AAS and traditional citation counts.^[2,10,22]^ Nevertheless, the situation differed for subgroup correlations among the sources of Altmetrics. A weak correlation was observed between blog mentions and WoS and Google Scholar citations.

Moreover, a very strong correlation was found between the number of Mendeley readers and both WoS and Google Scholar citations (*R* = 0.889, *P* < .001 and *R* = 0.905, *P* < .001). Numerous altmetric studies have documented a positive correlation between Mendeley readership and number of citations.^[24-26]^ Similar to the results of the present study, Ruan et al^[24]^ found that citations were positively correlated with download rates (*R* = 0.31, *P* = .021) and Mendeley readership (*R* = 0.46, *P* = .001). Likewise, in their altmetric analysis of systemic sclerosis research, Doskaliuk et al^[25]^ reported a strong correlation (*ρ* = .612, *P* < .001) between the number of Mendeley readers and the citation counts of scholarly articles. Mendeley is a reference manager and academic social network that allows users to organize their research. The reader count in Mendeley indicates the number of users who saved a particular article in the library.^[24]^ Not all altmetrics may be useful for assessing the potential impact of an article, but Mendeley reader count could be a potential metric for understanding the influence of an article within the scientific community.

In this analysis, we found that more than 40% of the articles were published in the United States, and these articles had higher AAS and WoS citations than non-US articles. Similar to our findings, in other studies, highly cited articles were mainly produced in the United States and United Kingdom.^[10,24]^

OA publications that are freely available to everyone allow the article to reach a wider audience and, therefore, may create more sharing potential on social media. Some studies have reported that OA articles resulted in significantly greater AAS and citations than Paywalled access (PA) articles.^[17,26]^ In a study investigating the impact of OA and PA publications on AAS, intensive care medicine publications available as OA papers resulted in higher AAS than PA papers, but this was not the case for anesthesia.^[17]^ Similarly, Alfouzan et al^[7]^ analyzed the bibliometric properties of the 100 most cited articles in the field of anesthesiology and found that 55% of the most cited articles were published in the OA format. In our analysis, we found that 84.2% of the papers were OA, and there was no significant difference in AAS and WoS citations between free access papers and those behind paywalls.

We scanned the data without time constraints. The top 500 papers on the AAS were published between 1985 and 2024. The top 500 AAS papers were published in the last 5 years. However, the highest AAS median value was 356.5 (258.8–488.8) in 2015 (Fig. 2). When articles from 2015 were examined, the majority were on opioids and cannabinoids. With regard to opioids, the exponential increase in deaths from opioid overdoses in the United States over the last decade may explain this situation. In October 2017, the US Department of Health and Human Services declared the opioid crisis to be a national public health emergency.^[27]^ Similarly, AAS has been high in the last 5 years. We hypothesized that the high AAS observed during these periods could be attributed to the unique circumstances of the COVID-19 pandemic. During this period, a significant reduction in social activities due to quarantines and lockdowns, along with people spending more time at home, may have led to an increase in online engagement. In addition, heightened health concerns may have driven a greater interest in medical and health-related publications.

The most common subject was algology, and a significant difference was observed in both AAS and WoS citations across different subjects (*P* = .036, *P* = .005, respectively) (Table 3). One potential reason why algology has emerged as the most frequently shared topic on social platforms may be its broad scope. In addition, pain and its management are universally relatable topics, as almost everyone experiences pain at some point in their lives, making it easier for the general public to understand and empathize with its content. This relatability may have contributed to the higher AAS, as it encouraged broader social dissemination and discussion. In our study, COVID-19-related publications comprised 9.4% (n = 47) of the total, with an average AAS of 369.0 (IQR: 224.0–632.0), making it one of the most common topics. The urgent demand for reliable information has led to a sharp increase in scientific publications and their dissemination via social media.^[28]^ According to an altmetric study on COVID-19, the first COVID-19 publication appeared on January 17, 2020, with a rapid increase to 27,387 articles by August 2020 and 30,637 by September 2020.^[28]^ The high AAS scores for these publications can be attributed to the global urgency and widespread public interest. In addition, it can be considered that rapid sharing on social media and news platforms increased the visibility of these publications, allowing them to reach a broader audience beyond the academic community.

### 4.3. Limitations

This study had some limitations. First, although this study was designed to conduct an altmetric study in anesthesiology journals, many of the papers in the list also included algology and intensive care topics closely related to anesthesiology. Anesthesiology is a very broad branch of medicine and the content of its publications is very broad. This diversity poses challenges in defining a precise subject group for this study. It is necessary to include a large number of papers to account for this breadth. Future studies should focus on a more narrowly defined set of topics for greater specificity in the analysis.

Second, 105,344 publications were identified across 64 journals in our study, with a median AAS of 2 (IQR: 0–4). Among the top 500 publications, the median AAS was 281.5 (IQR: 211.0–417.5). Expanding the analysis to include the entire sample rather than limiting it to the top 500 may yield a weaker correlation between AAS and citation counts, as it would capture a broader range of articles with diverse levels of attention and impact. Thus, these findings may not be fully generalizable and may primarily reflect trends among highly ranked publications. This limitation should be considered when interpreting the results because the observed correlation may not apply to publications with lower AAS scores.

Third, using the category “Anesthesiology” in the MJL search engine may have resulted in the exclusion of some high-IF journals. However, we were concerned that specifically selecting high-IF journals in the field of anesthesiology from the MJL and including papers related to this discipline might introduce potential selection bias. Therefore, journals were selected without considering the IF scores.

Finally, while a high AAS reflects the popularity of a paper, it can be influenced by online engagement and may be subject to manipulation. English-language publications may also reach a larger audience, while some works may receive limited online attention because of access restrictions. In addition, altmetric scores can fluctuate over time; early high attention may not be sustained as accounts close or interest wanes.^[16]^ Despite these limitations, AAS and altmetric analysis, particularly when combined with traditional metrics, can yield valuable insights into the impact of publications.

## 5. Conclusions

Although AAS did not significantly affect traditional scientific citations, the analysis of subgroup correlations revealed notable differences. Our results suggest that traditional scientific citations (WoS and Google Scholar citations) are strongly influenced by the number of Mendeley readers. These platforms may have a positive impact by increasing citations, and promoting interaction and visibility within the academic community. Further research is needed to understand these dynamics in academic discourse.

## Author contributions

**Conceptualization:** Dilek Ömür Arça, Başak Bayram, Murat Çetin.

**Data curation:** Dilek Ömür Arça, Nilay Boztaş, İsmail Erdemir.

**Methodology:** Dilek Ömür Arça, Başak Bayram, Murat Çetin.

**Writing – original draft:** Dilek Ömür Arça.

**Formal analysis:** Başak Bayram, Gönül Sağiroğlu.

**Writing – review & editing:** Başak Bayram, Gönül Sağiroğlu, Volkan Hanci.

**Software:** Nilay Boztaş, İsmail Erdemir.

**Supervision:** Volkan Hanci.

## Supplementary Material


