# Supplementary material for: An analysis of the top 500 anesthesiology publications with the highest altmetric attention scores
Source: Medicine (Baltimore). 2025 Feb 21;104(8):e41523. doi: 10.1097/MD.0000000000041523 (PMC11856929; doi:10.1097/MD.0000000000041523)
Supplement: Supplementary file 1 [file medi-104-e41523-s001.docx]

**Appendix 1 WoS Master Journal List, Category Anesthesiology**

ANESTHESIOLOGY

clear

**Search Results**

Found 64 results (Page 1)

shareShare These Results

A & A PRACTICE

Publisher:

**LIPPINCOTT WILLIAMS & WILKINS , TWO COMMERCE SQ, 2001 MARKET ST, PHILADELPHIA, USA, PA, 19103**

ISSN / eISSN:

**2575-3126**

*Web of Science* Core Collection:

**Emerging Sources Citation Index**

shareShare This Journal

View profile page

** Requires free login.*

ACTA ANAESTHESIOLOGICA BELGICA

Publisher:

**ACTA MEDICAL BELGICA , AVENUE CIRCULAIRE 138 A, BRUSSELS, BELGIUM, B-1180**

ISSN / eISSN:

**0001-5164 / 2736-5239**

*Web of Science* Core Collection:

**Emerging Sources Citation Index**

shareShare This Journal

View profile page

** Requires free login.*

ACTA ANAESTHESIOLOGICA SCANDINAVICA

Publisher:

**WILEY , 111 RIVER ST, HOBOKEN, USA, NJ, 07030-5774**

ISSN / eISSN:

**0001-5172 / 1399-6576**

*Web of Science* Core Collection:

**Science Citation Index Expanded**

Additional *Web of Science* Indexes:

**Biological Abstracts | BIOSIS Previews | Current Contents Clinical Medicine | Current Contents Life Sciences | Essential Science Indicators**

shareShare This Journal

View profile page

** Requires free login.*

AIN SHAMS JOURNAL OF ANESTHESIOLOGY


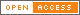


Publisher:

**AIN SHAMS UNIV , 1 Asmaa Fahmy Street, Cairo , Egypt, Heliopolis , 00000**

ISSN / eISSN:

**1687-7934 / 2090-925X**

*Web of Science* Core Collection:

**Emerging Sources Citation Index**

shareShare This Journal

View profile page

** Requires free login.*

ANAESTHESIA

Publisher:

**WILEY , 111 RIVER ST, HOBOKEN, USA, NJ, 07030-5774**

ISSN / eISSN:

**0003-2409 / 1365-2044**

*Web of Science* Core Collection:

**Science Citation Index Expanded**

Additional *Web of Science* Indexes:

**Current Contents Clinical Medicine | Current Contents Life Sciences | Essential Science Indicators**

shareShare This Journal

View profile page

** Requires free login.*

ANAESTHESIA AND INTENSIVE CARE

Publisher:

**SAGE PUBLICATIONS LTD , 1 OLIVERS YARD, 55 CITY ROAD, LONDON, ENGLAND, EC1Y 1SP**

ISSN / eISSN:

**0310-057X / 1448-0271**

*Web of Science* Core Collection:

**Science Citation Index Expanded**

Additional *Web of Science* Indexes:

**Current Contents Clinical Medicine | Essential Science Indicators**

shareShare This Journal

View profile page

** Requires free login.*

ANAESTHESIA AND INTENSIVE CARE MEDICINE

Publisher:

**ELSEVIER , RADARWEG 29, AMSTERDAM, Netherlands, 1043 NX**

ISSN / eISSN:

**1472-0299 / 1878-7584**

*Web of Science* Core Collection:

**Emerging Sources Citation Index**

shareShare This Journal

View profile page

** Requires free login.*

ANAESTHESIA CRITICAL CARE & PAIN MEDICINE

Publisher:

**ELSEVIER FRANCE-EDITIONS SCIENTIFIQUES MEDICALES ELSEVIER , 65 RUE CAMILLE DESMOULINS, CS50083, ISSY-LES-MOULINEAUX, FRANCE, 92442**

ISSN / eISSN:

**2352-5568**

*Web of Science* Core Collection:

**Science Citation Index Expanded**

Additional *Web of Science* Indexes:

**Biological Abstracts | BIOSIS Previews | Current Contents Clinical Medicine | Essential Science Indicators**

shareShare This Journal

View profile page

** Requires free login.*

ANAESTHESIA PAIN & INTENSIVE CARE


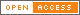


Publisher:

**ANAESTHESIA PAIN & INTENSIVE CARE , C/O TARIQ HAYAT KHAN, ED, 60-A, NAZIM-UD-DIN RD, ISLAMABAD, PAKISTAN, 00000**

ISSN / eISSN:

**1607-8322 / 2220-5799**

*Web of Science* Core Collection:

**Emerging Sources Citation Index**

shareShare This Journal

View profile page

** Requires free login.*

ANAESTHESIA REPORTS

Publisher:

**WILEY , 111 RIVER ST, HOBOKEN, USA, NJ, 07030-5774**

ISSN / eISSN:

**2637-3726**

*Web of Science* Core Collection:

**Emerging Sources Citation Index**

shareShare This Journal

View profile page

** Requires free login.*

**Active Filters info**

ANESTHESIOLOGY

clear

**Search Results**

Found 64 results (Page 2)

shareShare These Results

ANAESTHESIOLOGIE

Publisher:

**SPRINGER HEIDELBERG , TIERGARTENSTRASSE 17, HEIDELBERG, GERMANY, D-69121**

ISSN / eISSN:

**2731-6858 / 2731-6866**

*Web of Science* Core Collection:

**Science Citation Index Expanded**

Additional *Web of Science* Indexes:

**Biological Abstracts | BIOSIS Previews | Current Contents Clinical Medicine | Essential Science Indicators**

shareShare This Journal

View profile page

** Requires free login.*

ANAESTHESIOLOGY INTENSIVE THERAPY


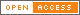


Publisher:

**TERMEDIA PUBLISHING HOUSE LTD , KLEEBERGA 2, POZNAN, Poland, 61-615**

ISSN / eISSN:

**1642-5758 / 1731-2531**

*Web of Science* Core Collection:

**Emerging Sources Citation Index**

shareShare This Journal

View profile page

** Requires free login.*

ANASTHESIOLOGIE & INTENSIVMEDIZIN

Publisher:

**AKTIV DRUCK & VERLAG GMBH , AN DER LOHWIESE 36, EBELSBACH, GERMANY, 97500**

ISSN / eISSN:

**0170-5334 / 1439-0256**

*Web of Science* Core Collection:

**Science Citation Index Expanded**

Additional *Web of Science* Indexes:

**Current Contents Clinical Medicine | Essential Science Indicators**

shareShare This Journal

View profile page

** Requires free login.*

ANASTHESIOLOGIE INTENSIVMEDIZIN NOTFALLMEDIZIN SCHMERZTHERAPIE

Publisher:

**GEORG THIEME VERLAG KG , RUDIGERSTR 14, STUTTGART, GERMANY, D-70469**

ISSN / eISSN:

**0939-2661 / 1439-1074**

*Web of Science* Core Collection:

**Science Citation Index Expanded**

Additional *Web of Science* Indexes:

**Essential Science Indicators**

shareShare This Journal

View profile page

** Requires free login.*

ANESTEZIOLOGIE A INTENZIVNI MEDICINA

Publisher:

**SOLEN SRO , Lazecka 297/51, 51, Olomouc, Czech Republic, 779 00**

ISSN / eISSN:

**1214-2158 / 1805-4412**

*Web of Science* Core Collection:

**Emerging Sources Citation Index**

shareShare This Journal

View profile page

** Requires free login.*

ANESTHESIA AND ANALGESIA

Publisher:

**LIPPINCOTT WILLIAMS & WILKINS , TWO COMMERCE SQ, 2001 MARKET ST, PHILADELPHIA, USA, PA, 19103**

ISSN / eISSN:

**0003-2999**

*Web of Science* Core Collection:

**Science Citation Index Expanded**

Additional *Web of Science* Indexes:

**Biological Abstracts | BIOSIS Previews | Current Contents Clinical Medicine | Current Contents Life Sciences | Essential Science Indicators**

shareShare This Journal

View profile page

** Requires free login.*

ANESTHESIE & REANIMATION

Publisher:

**ELSEVIER MASSON, CORPORATION OFFICE , 65 CAMILLE DESMOULINS CS50083 ISSY-LES-MOULINEAUX, PARIS, FRANCE, 92442**

ISSN / eISSN:

**2352-5800 / 2352-5819**

*Web of Science* Core Collection:

**Emerging Sources Citation Index**

shareShare This Journal

View profile page

** Requires free login.*

ANESTHESIOLOGY

Publisher:

**LIPPINCOTT WILLIAMS & WILKINS , TWO COMMERCE SQ, 2001 MARKET ST, PHILADELPHIA, USA, PA, 19103**

ISSN / eISSN:

**0003-3022 / 1528-1175**

*Web of Science* Core Collection:

**Science Citation Index Expanded**

Additional *Web of Science* Indexes:

**Biological Abstracts | BIOSIS Previews | Current Contents Clinical Medicine | Current Contents Life Sciences | Essential Science Indicators**

shareShare This Journal

View profile page

** Requires free login.*

ANESTHESIOLOGY RESEARCH AND PRACTICE


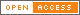


Publisher:

**HINDAWI LTD , ADAM HOUSE, 3RD FLR, 1 FITZROY SQ, LONDON, ENGLAND, W1T 5HF**

ISSN / eISSN:

**1687-6962 / 1687-6970**

*Web of Science* Core Collection:

**Emerging Sources Citation Index**

shareShare This Journal

View profile page

** Requires free login.*

ANNALS OF CARDIAC ANAESTHESIA


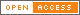


Publisher:

**WOLTERS KLUWER MEDKNOW PUBLICATIONS , WOLTERS KLUWER INDIA PVT LTD , A-202, 2ND FLR, QUBE, C T S NO 1498A-2 VILLAGE MAROL, ANDHERI EAST, MUMBAI, India, Maharashtra, 400059**

ISSN / eISSN:

**0971-9784 / 0974-5181**

*Web of Science* Core Collection:

**Emerging Sources Citation Index**

shareShare This Journal

View profile page

**Active Filters info**

ANESTHESIOLOGY

clear

**Search Results**

Found 64 results (Page 3)

shareShare These Results

BEST PRACTICE & RESEARCH-CLINICAL ANAESTHESIOLOGY

Publisher:

**ELSEVIER , RADARWEG 29, AMSTERDAM, Netherlands, 1043 NX**

ISSN / eISSN:

**1521-6896 / 1878-1608**

*Web of Science* Core Collection:

**Science Citation Index Expanded**

Additional *Web of Science* Indexes:

**Current Contents Clinical Medicine | Essential Science Indicators**

shareShare This Journal

View profile page

** Requires free login.*

BJA EDUCATION

Publisher:

**ELSEVIER SCI LTD , 125 London Wall, London, England, EC2Y 5AS**

ISSN / eISSN:

**2058-5349 / 2058-5357**

*Web of Science* Core Collection:

**Emerging Sources Citation Index**

shareShare This Journal

View profile page

** Requires free login.*

BMC ANESTHESIOLOGY


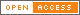


Publisher:

**BMC , CAMPUS, 4 CRINAN ST, LONDON, ENGLAND, N1 9XW**

ISSN / eISSN:

**1471-2253**

*Web of Science* Core Collection:

**Science Citation Index Expanded**

Additional *Web of Science* Indexes:

**Essential Science Indicators**

shareShare This Journal

View profile page

** Requires free login.*

BRAZILIAN JOURNAL OF ANESTHESIOLOGY


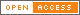


Publisher:

**ELSEVIER SCIENCE INC , STE 800, 230 PARK AVE, NEW YORK, USA, NY, 10169**

ISSN / eISSN:

**0104-0014 / 2352-2291**

*Web of Science* Core Collection:

**Science Citation Index Expanded**

Additional *Web of Science* Indexes:

**Essential Science Indicators**

shareShare This Journal

View profile page

** Requires free login.*

BRITISH JOURNAL OF ANAESTHESIA

Publisher:

**ELSEVIER SCI LTD , 125 London Wall, London, England, EC2Y 5AS**

ISSN / eISSN:

**0007-0912 / 1471-6771**

*Web of Science* Core Collection:

**Science Citation Index Expanded**

Additional *Web of Science* Indexes:

**Biological Abstracts | BIOSIS Previews | Current Contents Clinical Medicine | Current Contents Life Sciences | Essential Science Indicators**

shareShare This Journal

View profile page

** Requires free login.*

CANADIAN JOURNAL OF ANESTHESIA-JOURNAL CANADIEN D ANESTHESIE

Publisher:

**SPRINGER , ONE NEW YORK PLAZA, SUITE 4600 , NEW YORK, United States, NY, 10004**

ISSN / eISSN:

**0832-610X / 1496-8975**

*Web of Science* Core Collection:

**Science Citation Index Expanded**

Additional *Web of Science* Indexes:

**Biological Abstracts | BIOSIS Previews | Current Contents Clinical Medicine | Current Contents Life Sciences | Essential Science Indicators**

shareShare This Journal

View profile page

** Requires free login.*

CLINICAL JOURNAL OF PAIN

Publisher:

**LIPPINCOTT WILLIAMS & WILKINS , TWO COMMERCE SQ, 2001 MARKET ST, PHILADELPHIA, USA, PA, 19103**

ISSN / eISSN:

**0749-8047 / 1536-5409**

*Web of Science* Core Collection:

**Science Citation Index Expanded**

Additional *Web of Science* Indexes:

**Current Contents Clinical Medicine | Essential Science Indicators**

shareShare This Journal

View profile page

** Requires free login.*

CURRENT ANESTHESIOLOGY REPORTS

Publisher:

**SPRINGERNATURE , CAMPUS, 4 CRINAN ST, LONDON, ENGLAND, N1 9XW**

ISSN / eISSN:

**2167-6275**

*Web of Science* Core Collection:

**Emerging Sources Citation Index**

shareShare This Journal

View profile page

** Requires free login.*

CURRENT OPINION IN ANESTHESIOLOGY

Publisher:

**LIPPINCOTT WILLIAMS & WILKINS , TWO COMMERCE SQ, 2001 MARKET ST, PHILADELPHIA, USA, PA, 19103**

ISSN / eISSN:

**0952-7907 / 1473-6500**

*Web of Science* Core Collection:

**Science Citation Index Expanded**

Additional *Web of Science* Indexes:

**Current Contents Clinical Medicine | Essential Science Indicators**

shareShare This Journal

View profile page

** Requires free login.*

EGYPTIAN JOURNAL OF ANAESTHESIA


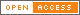


Publisher:

**TAYLOR & FRANCIS LTD , 2-4 PARK SQUARE, MILTON PARK, ABINGDON, England, OXON, OX14 4RN**

ISSN / eISSN:

**1110-1849**

*Web of Science* Core Collection:

**Emerging Sources Citation Index**

shareShare This Journal

View profile page

** Requires free login.*

Items per page:

10

21 – 30 of 64

**Search Results**

Found 64 results (Page 4)

shareShare These Results

EUROPEAN JOURNAL OF ANAESTHESIOLOGY

Publisher:

**LIPPINCOTT WILLIAMS & WILKINS , TWO COMMERCE SQ, 2001 MARKET ST, PHILADELPHIA, USA, PA, 19103**

ISSN / eISSN:

**0265-0215 / 1365-2346**

*Web of Science* Core Collection:

**Science Citation Index Expanded**

Additional *Web of Science* Indexes:

**Biological Abstracts | BIOSIS Previews | Current Contents Clinical Medicine | Essential Science Indicators**

shareShare This Journal

View profile page

** Requires free login.*

EUROPEAN JOURNAL OF PAIN

Publisher:

**WILEY , 111 RIVER ST, HOBOKEN, USA, NJ, 07030-5774**

ISSN / eISSN:

**1090-3801 / 1532-2149**

*Web of Science* Core Collection:

**Science Citation Index Expanded**

Additional *Web of Science* Indexes:

**Current Contents Clinical Medicine | Essential Science Indicators**

shareShare This Journal

View profile page

** Requires free login.*

INDIAN ANAESTHETISTS FORUM


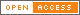


Publisher:

**WOLTERS KLUWER MEDKNOW PUBLICATIONS , WOLTERS KLUWER INDIA PVT LTD , A-202, 2ND FLR, QUBE, C T S NO 1498A-2 VILLAGE MAROL, ANDHERI EAST, MUMBAI, India, Maharashtra, 400059**

ISSN / eISSN:

**2589-7934 / 0973-0311**

*Web of Science* Core Collection:

**Emerging Sources Citation Index**

shareShare This Journal

View profile page

** Requires free login.*

INDIAN JOURNAL OF ANAESTHESIA


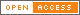


Publisher:

**WOLTERS KLUWER MEDKNOW PUBLICATIONS , WOLTERS KLUWER INDIA PVT LTD , A-202, 2ND FLR, QUBE, C T S NO 1498A-2 VILLAGE MAROL, ANDHERI EAST, MUMBAI, India, Maharashtra, 400059**

ISSN / eISSN:

**0019-5049 / 0976-2817**

*Web of Science* Core Collection:

**Emerging Sources Citation Index**

shareShare This Journal

View profile page

** Requires free login.*

INTERNATIONAL ANESTHESIOLOGY CLINICS

Publisher:

**LIPPINCOTT WILLIAMS & WILKINS , TWO COMMERCE SQ, 2001 MARKET ST, PHILADELPHIA, USA, PA, 19103**

ISSN / eISSN:

**0020-5907 / 1537-1913**

*Web of Science* Core Collection:

**Emerging Sources Citation Index**

shareShare This Journal

View profile page

** Requires free login.*

INTERNATIONAL JOURNAL OF OBSTETRIC ANESTHESIA

Publisher:

**ELSEVIER SCI LTD , 125 London Wall, London, England, EC2Y 5AS**

ISSN / eISSN:

**0959-289X / 1532-3374**

*Web of Science* Core Collection:

**Science Citation Index Expanded**

Additional *Web of Science* Indexes:

**Current Contents Clinical Medicine | Essential Science Indicators**

shareShare This Journal

View profile page

** Requires free login.*

JA CLINICAL REPORTS


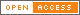


Publisher:

**SPRINGER , ONE NEW YORK PLAZA, SUITE 4600 , NEW YORK, United States, NY, 10004**

ISSN / eISSN:

**2363-9024**

*Web of Science* Core Collection:

**Emerging Sources Citation Index**

shareShare This Journal

View profile page

** Requires free login.*

JOURNAL OF ANESTHESIA

Publisher:

**SPRINGER JAPAN KK , SHIROYAMA TRUST TOWER 5F, 4-3-1 TORANOMON, MINATO-KU, TOKYO, JAPAN, 105-6005**

ISSN / eISSN:

**0913-8668 / 1438-8359**

*Web of Science* Core Collection:

**Science Citation Index Expanded**

Additional *Web of Science* Indexes:

**Essential Science Indicators**

shareShare This Journal

View profile page

** Requires free login.*

JOURNAL OF CARDIOTHORACIC AND VASCULAR ANESTHESIA

Publisher:

**W B SAUNDERS CO-ELSEVIER INC , 1600 JOHN F KENNEDY BOULEVARD, STE 1800, PHILADELPHIA, USA, PA, 19103-2899**

ISSN / eISSN:

**1053-0770 / 1532-8422**

*Web of Science* Core Collection:

**Science Citation Index Expanded**

Additional *Web of Science* Indexes:

**Current Contents Clinical Medicine | Essential Science Indicators**

shareShare This Journal

View profile page

** Requires free login.*

JOURNAL OF CLINICAL ANESTHESIA

Publisher:

**ELSEVIER SCIENCE INC , STE 800, 230 PARK AVE, NEW YORK, USA, NY, 10169**

ISSN / eISSN:

**0952-8180 / 1873-4529**

*Web of Science* Core Collection:

**Science Citation Index Expanded**

Additional *Web of Science* Indexes:

**Current Contents Clinical Medicine | Essential Science Indicators**

**Active Filters info**

ANESTHESIOLOGY

clear

**Search Results**

Found 64 results (Page 5)

shareShare These Results

JOURNAL OF CLINICAL MONITORING AND COMPUTING

Publisher:

**SPRINGER HEIDELBERG , TIERGARTENSTRASSE 17, HEIDELBERG, GERMANY, D-69121**

ISSN / eISSN:

**1387-1307 / 1573-2614**

*Web of Science* Core Collection:

**Science Citation Index Expanded**

Additional *Web of Science* Indexes:

**Current Contents Clinical Medicine | Essential Science Indicators**

shareShare This Journal

View profile page

** Requires free login.*

JOURNAL OF NEUROANAESTHESIOLOGY AND CRITICAL CARE


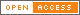


Publisher:

**GEORG THIEME VERLAG KG , RUDIGERSTR 14, STUTTGART, GERMANY, D-70469**

ISSN / eISSN:

**2348-0548 / 2348-926X**

*Web of Science* Core Collection:

**Emerging Sources Citation Index**

shareShare This Journal

View profile page

** Requires free login.*

JOURNAL OF NEUROSURGICAL ANESTHESIOLOGY

Publisher:

**LIPPINCOTT WILLIAMS & WILKINS , TWO COMMERCE SQ, 2001 MARKET ST, PHILADELPHIA, USA, PA, 19103**

ISSN / eISSN:

**0898-4921 / 1537-1921**

*Web of Science* Core Collection:

**Science Citation Index Expanded**

Additional *Web of Science* Indexes:

**Current Contents Clinical Medicine | Essential Science Indicators**

shareShare This Journal

View profile page

** Requires free login.*

JOURNAL OF OBSTETRIC ANAESTHESIA AND CRITICAL CARE


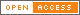


Publisher:

**WOLTERS KLUWER MEDKNOW PUBLICATIONS , WOLTERS KLUWER INDIA PVT LTD , A-202, 2ND FLR, QUBE, C T S NO 1498A-2 VILLAGE MAROL, ANDHERI EAST, MUMBAI, India, Maharashtra, 400059**

ISSN / eISSN:

**2249-4472 / 2249-9539**

*Web of Science* Core Collection:

**Emerging Sources Citation Index**

shareShare This Journal

View profile page

** Requires free login.*

JOURNAL OF PAIN & PALLIATIVE CARE PHARMACOTHERAPY

Publisher:

**TAYLOR & FRANCIS INC , 530 WALNUT STREET, STE 850, PHILADELPHIA, USA, PA, 19106**

ISSN / eISSN:

**1536-0288 / 1536-0539**

*Web of Science* Core Collection:

**Emerging Sources Citation Index**

shareShare This Journal

View profile page

** Requires free login.*

KOREAN JOURNAL OF ANESTHESIOLOGY


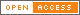


Publisher:

**KOREAN SOC ANESTHESIOLOGISTS , 101-3503, LOTTE CASTLE PRESIDENT, 109 MAPO-DAERO, MAPO-GU, SEOUL, South Korea, 04146**

ISSN / eISSN:

**2005-6419 / 2005-7563**

*Web of Science* Core Collection:

**Science Citation Index Expanded**

Additional *Web of Science* Indexes:

**Current Contents Clinical Medicine | Essential Science Indicators**

shareShare This Journal

View profile page

** Requires free login.*

LOCAL AND REGIONAL ANESTHESIA


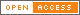


Publisher:

**DOVE MEDICAL PRESS LTD , PO BOX 300-008, ALBANY, NEW ZEALAND, AUCKLAND, 0752**

ISSN / eISSN:

**1178-7112**

*Web of Science* Core Collection:

**Emerging Sources Citation Index**

shareShare This Journal

View profile page

** Requires free login.*

MINERVA ANESTESIOLOGICA

Publisher:

**EDIZIONI MINERVA MEDICA , CORSO BRAMANTE 83-85 INT JOURNALS DEPT., TURIN, ITALY, 10126**

ISSN / eISSN:

**0375-9393 / 1827-1596**

*Web of Science* Core Collection:

**Science Citation Index Expanded**

Additional *Web of Science* Indexes:

**Current Contents Clinical Medicine | Essential Science Indicators**

shareShare This Journal

View profile page

** Requires free login.*

PAIN

Publisher:

**LIPPINCOTT WILLIAMS & WILKINS , TWO COMMERCE SQ, 2001 MARKET ST, PHILADELPHIA, USA, PA, 19103**

ISSN / eISSN:

**0304-3959 / 1872-6623**

*Web of Science* Core Collection:

**Science Citation Index Expanded**

Additional *Web of Science* Indexes:

**Biological Abstracts | BIOSIS Previews | Current Contents Clinical Medicine | Current Contents Life Sciences | Essential Science Indicators**

shareShare This Journal

View profile page

** Requires free login.*

PAIN MEDICINE

Publisher:

**OXFORD UNIV PRESS , GREAT CLARENDON ST, OXFORD, ENGLAND, OX2 6DP**

ISSN / eISSN:

**1526-2375 / 1526-4637**

*Web of Science* Core Collection:

**Science Citation Index Expanded**

Additional *Web of Science* Indexes:

**Current Contents Clinical Medicine | Essential Science Indicators**

shareShare This Journal

View profile page

ANESTHESIOLOGY

clear

**Search Results**

Found 64 results (Page 6)

shareShare These Results

PAIN PHYSICIAN

Publisher:

**AM SOC INTERVENTIONAL PAIN PHYSICIANS , 81 LAKEVIEW DR, PADUCAH, USA, KY, 42001**

ISSN / eISSN:

**1533-3159 / 2150-1149**

*Web of Science* Core Collection:

**Science Citation Index Expanded**

Additional *Web of Science* Indexes:

**Current Contents Clinical Medicine | Essential Science Indicators**

shareShare This Journal

View profile page

** Requires free login.*

PAIN PRACTICE

Publisher:

**WILEY , 111 RIVER ST, HOBOKEN, USA, NJ, 07030-5774**

ISSN / eISSN:

**1530-7085 / 1533-2500**

*Web of Science* Core Collection:

**Science Citation Index Expanded**

Additional *Web of Science* Indexes:

**Current Contents Clinical Medicine | Essential Science Indicators**

shareShare This Journal

View profile page

** Requires free login.*

PEDIATRIC ANESTHESIA

Publisher:

**WILEY , 111 RIVER ST, HOBOKEN, USA, NJ, 07030-5774**

ISSN / eISSN:

**1155-5645 / 1460-9592**

*Web of Science* Core Collection:

**Science Citation Index Expanded**

Additional *Web of Science* Indexes:

**Current Contents Clinical Medicine | Essential Science Indicators**

shareShare This Journal

View profile page

** Requires free login.*

PEDIATRIC ANESTHESIA AND CRITICAL CARE JOURNAL


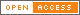


Publisher:

**UNIV HOSPITAL OSPEDALI RIUNITI , UNIV DEPT ANESTHESIA & INTENSIVE CARE, FOGGIA, ITALY, 00000**

ISSN / eISSN:

**2281-8421**

*Web of Science* Core Collection:

**Emerging Sources Citation Index**

shareShare This Journal

View profile page

** Requires free login.*

PERIOPERATIVE MEDICINE


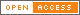


Publisher:

**BMC , CAMPUS, 4 CRINAN ST, LONDON, ENGLAND, N1 9XW**

ISSN / eISSN:

**2047-0525**

*Web of Science* Core Collection:

**Science Citation Index Expanded**

Additional *Web of Science* Indexes:

**Current Contents Clinical Medicine | Essential Science Indicators**

shareShare This Journal

View profile page

** Requires free login.*

REGIONAL ANESTHESIA AND PAIN MEDICINE

Publisher:

**BMJ PUBLISHING GROUP , BRITISH MED ASSOC HOUSE, TAVISTOCK SQUARE, LONDON, ENGLAND, WC1H 9JR**

ISSN / eISSN:

**1098-7339 / 1532-8651**

*Web of Science* Core Collection:

**Science Citation Index Expanded**

Additional *Web of Science* Indexes:

**Biological Abstracts | BIOSIS Previews | Current Contents Clinical Medicine | Essential Science Indicators**

shareShare This Journal

View profile page

** Requires free login.*

REVISTA ESPANOLA DE ANESTESIOLOGIA Y REANIMACION

Publisher:

**ELSEVIER , RADARWEG 29, AMSTERDAM, Netherlands, 1043 NX**

ISSN / eISSN:

**0034-9356 / 2340-3284**

*Web of Science* Core Collection:

**Emerging Sources Citation Index**

shareShare This Journal

View profile page

** Requires free login.*

SAUDI JOURNAL OF ANAESTHESIA


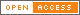


Publisher:

**WOLTERS KLUWER MEDKNOW PUBLICATIONS , WOLTERS KLUWER INDIA PVT LTD , A-202, 2ND FLR, QUBE, C T S NO 1498A-2 VILLAGE MAROL, ANDHERI EAST, MUMBAI, India, Maharashtra, 400059**

ISSN / eISSN:

**1658-354X / 0975-3125**

*Web of Science* Core Collection:

**Emerging Sources Citation Index**

shareShare This Journal

View profile page

** Requires free login.*

SCHMERZ

Publisher:

**SPRINGER HEIDELBERG , TIERGARTENSTRASSE 17, HEIDELBERG, GERMANY, D-69121**

ISSN / eISSN:

**0932-433X / 1432-2129**

*Web of Science* Core Collection:

**Science Citation Index Expanded**

Additional *Web of Science* Indexes:

**Current Contents Clinical Medicine | Essential Science Indicators**

shareShare This Journal

View profile page

** Requires free login.*

SEMINARS IN CARDIOTHORACIC AND VASCULAR ANESTHESIA

Publisher:

**SAGE PUBLICATIONS INC , 2455 TELLER RD, THOUSAND OAKS, USA, CA, 91320**

ISSN / eISSN:

**1089-2532 / 1940-5596**

*Web of Science* Core Collection:

**Emerging Sources Citation Index**

shareShare This Journal

View profile page

** Requires free login.*

Items per page:

10

51 – 60 of 64

ANESTHESIOLOGY

clear

**Search Results**

Found 64 results (Page 7)

shareShare These Results

SOUTHERN AFRICAN JOURNAL OF ANAESTHESIA AND ANALGESIA

Publisher:

**MEDPHARM PUBLICATIONS PTY LTD , PO BOX 14804 LYTTELTON MANOR, CENTURION, SOUTH AFRICA, 0140**

ISSN / eISSN:

**2220-1181 / 2220-1173**

*Web of Science* Core Collection:

**Emerging Sources Citation Index**

shareShare This Journal

View profile page

** Requires free login.*

SRI LANKAN JOURNAL OF ANAESTHESIOLOGY


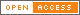


Publisher:

**COLL ANAESTHESIOLOGISTS SRI LANKA , 6 WIJERAMA MAWATHA, COLOMBO, SRI LANKA, 7**

ISSN / eISSN:

**1391-8834 / 2279-1965**

*Web of Science* Core Collection:

**Emerging Sources Citation Index**

shareShare This Journal

View profile page

** Requires free login.*

TRENDS IN ANAESTHESIA AND CRITICAL CARE

Publisher:

**ELSEVIER SCI LTD , 125 London Wall, London, England, EC2Y 5AS**

ISSN / eISSN:

**2210-8440 / 2210-8467**

*Web of Science* Core Collection:

**Emerging Sources Citation Index**

shareShare This Journal

View profile page

** Requires free login.*

TURKISH JOURNAL OF ANAESTHESIOLOGY AND REANIMATION


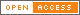


Publisher:

**GALENOS PUBL HOUSE , Kacamak Sokak 21/1, ISTANBUL, Turkiye, Findikzade, 34093**

ISSN / eISSN:

**2667-6370**

*Web of Science* Core Collection:

**Emerging Sources Citation Index**

shareShare This Journal

View profile page

** Requires free login.*

| Appendix 2: The Top 500 AAS Publications in Anesthesiology list. | | | | | | | | | | | | | |
| --- | --- | --- | --- | --- | --- | --- | --- | --- | --- | --- | --- | --- | --- |
| **Rank** | **AAS** | **Title** | **First Author** | **Journal/**  **Collection Title** | **Year** | **OA** | **Country** | **Subject** | **Study type** | **WoS**  **Citation** | **Google Schooler Citation** | **Wos/**  **Years** | **Scholar Citation**  **/Years** |
| 1 | 2731 | Timing of surgery following SARS-CoV? infection: an international prospective cohort study | COVIDSurg Collaborative; GlobalSurg Collaborative. | Anaesthesia | 2021 | Free | UK | COVID19 | Observational Study | 279 | 405 | 69.75 | 101.25 |
| 2 | 2407 | Microdeletion in a FAAH pseudogene identified in a patient with high anandamide concentrations and pain insensitivity | Habib AM. | BJA: The British Journal of Anaesthesia | 2019 | Free | UK | Algology | Case report | 67 | 117 | 11.17 | 19.50 |
| 3 | 1967 | Aspirin Use Is Associated With Decreased Mechanical Ventilation. Intensive Care Unit Admission. and In-Hospital Mortality in Hospitalized Patients With Coronavirus Disease 2019. | Chow JH. | Anesthesia and analgesia | 2020 | Free | USA | COVID-19 | Retrospective | 211 | 356 | 42.20 | 71.20 |
| 4 | 1966 | Open-label placebo treatment in chronic low back pain | Carvalho C. | Pain (03043959) | 2016 | Free | Portugal | Algology | RCT | 275 | 446 | 30.56 | 49.56 |
| 5 | 1720 | Anesthesia and glucagon-like peptide-1 receptor agonists: proceed with caution! | Jones PM. | Canadian Journal of Anesthesia/Journal canadien d'anesthésie | 2023 | Free | USA | Anesthesia practice | Editorial | 13 | 28 | 6.50 | 14.00 |
| 6 | 1674 | Semaglutide. delayed gastric emptying. and intraoperative pulmonary aspiration: a case report | Klein SR. | Canadian Journal of Anesthesia/Journal canadien d'anesthésie | 2023 | Free | USA | Anesthesia practice | Case report | 30 | 61 | 15.00 | 30.50 |
| 7 | 1654 | Relationship between perioperative semaglutide use and residual gastric content: A retrospective analysis of patients undergoing elective upper endoscopy | Silveira SQ. | Journal of Clinical Anesthesia | 2023 | Free | Brasil | Anesthesia practice | Retrospective study | 30 | 74 | 15.00 | 37.00 |
| 8 | 1502 | COVID?19 vaccines: one step towards the beginning of the end of the global impact of the pandemic | Cook TM. | Anaesthesia | 2020 | Free | UK | COVID-19 | Editorial | 4 | 5 | 0.80 | 1.00 |
| 9 | 1428 | Outcomes from intensive care in patients with COVID-19: a systematic review and meta-analysis of observational studies | Armstrong RA. | Anaesthesia | 2020 | Free | UK | COVID 19 | Review | 273 | 437 | 54.60 | 87.40 |
| 10 | 1358 | A quantitative evaluation of aerosol generation during tracheal intubation and extubation | Brown J. | Anaesthesia | 2020 | Free | UK | HCWH | Observational Study | 129 | 206 | 25.80 | 41.20 |
| 11 | 1343 | Cytokines. Inflammation. and Pain | Zhang JM. | International Anesthesiology Clinics | 2007 | Free | China. USA. Ohio | Algology | Review | 1785 | 3772 | 99.17 | 209.56 |
| 12 | 1327 | The effect of sleep deprivation and disruption on DNA damage and health of doctors | Cheung V. | Anaesthesia | 2019 | Free | UK. Hong Kong | HCWS | Observational Study | 39 | 75 | 6.50 | 12.50 |
| 13 | 1259 | The effect of respiratory activity. non-invasive respiratory support and facemasks on aerosol generation and its relevance to COVID-19 | Wilson NM. | Anaesthesia | 2021 | Free | Australia | COVID-19 | Observational Study | 79 | 121 | 19.75 | 30.25 |
| 14 | 1218 | Transdermal cannabidiol reduces inflammation and pain-related behaviours in a rat model of arthritis | Hammell DC. | European Journal of Pain | 2015 | Free | USA | Algology | Experimental study | 183 | 416 | 18.30 | 41.60 |
| 15 | 1170 | Early-life adversity increases morphine tolerance and persistent inflammatory hypersensitivity through upregulation of opioid receptors in mice | Singleton S. | Pain (03043959) | 2023 | Free | UK | Algoloy | Experimental study | 0 | 1 | 0.00 | 0.50 |
| 16 | 1086 | COVID-19 and extracorporeal membrane oxygenation: experiences as a patient. general practitioner. wife and mother | Gupta A. | Anaesthesia Reports | 2021 | Free | UK | COVID-19 | Case report | 4 | 4 | 1.00 | 1.00 |
| 17 | 1054 | Data fabrication and other reasons for non-random sampling in 5087 randomised. controlled trials in anaesthetic and general medical journals | Carlisle JB. | Anaesthesia | 2017 | Free | UK | Others | RCT | 123 | 201 | 15.38 | 25.13 |
| 18 | 1023 | Effects of Skin Pigmentation on Pulse Oximeter Accuracy at Low Saturation | Bickler PE. | Anesthesiology | 2005 | Free | USA | Monitorization | Observational Study | 212 | 390 | 10.60 | 19.50 |
| 19 | 1008 | A junior doctor’s experience of critical illness: from treating patients to becoming a patient with COVID-19 | Ramachandran S. | Anaesthesia Reports | 2020 | Free | UK | COVID-19 | Case report | 6 | 9 | 1.20 | 1.80 |
| 20 | 971 | General anaesthesia in end of life care: extending the indications for anaesthesia beyond surgery | Takla A. | Anaesthesia | 2021 | Free | UK | Anesthesia practise | Review | 9 | 13 | 2.25 | 3.25 |
| 21 | 955 | Cannabidiol modulates serotonergic transmission and reverses both allodynia and anxiety-like behavior in a model of neuropathic pain | De Gregorio D. | Pain (03043959) | 2018 | Free | Canada | Algology | Experimental study | 212 | 357 | 30.29 | 51.00 |
| 22 | 950 | The sociocultural context of pediatric pain: an examination of the portrayal of pain in children's popular media. | Mueri K. | Pain (03043959) | 2021 | No | Canada | Algology | Observational study | 9 | 14 | 2.25 | 3.50 |
| 23 | 923 | The revised International Association for the Study of Pain definition of pain: concepts. challenges. and compromises. | Raja SN. | Pain (03043959) | 2020 | Free | USA | Algology | Survey | 1481 | 3890 | 296.20 | 778.00 |
| 24 | 905 | SARS-CoV-2 spike protein co-opts VEGF-A/neuropilin-1 receptor signaling to induce analgesia | Moutal A. | Pain (03043959) | 2020 | Free | USA | COVID-19 | Experimental study | 63 | 152 | 12.60 | 30.40 |
| 25 | 887 | Disentangling self from pain: mindfulness meditation–induced pain relief is driven by thalamic–default mode network decoupling | Riegner G | Pain (03043959) | 2022 | Free | USA | Algology | RCT | 9 | 20 | 3.00 | 6.67 |
| 26 | 867 | Time to Take Stock: A Meta-Analysis and Systematic Review of Analgesic Treatment Disparities for Pain in the United States | Meghani SH. | Pain Medicine | 2012 | Free | USA | Algology | Reviev | 321 | 505 | 24.69 | 38.85 |
| 27 | 862 | Prevalence of chronic pain among adults in the United States | Yong RJ. | Pain (03043959) | 2021 | No | USA | Algology | Retrospective study | 254 | 497 | 63.50 | 124.25 |
| 28 | 829 | Recent cannabis use and nightly sleep duration in adults: a population analysis of the NHANES from 2005 to 2018 | Diep C. | Regional Anesthesia & Pain Medicine | 2021 | Free | Canada | Algology | Retrospective study | 9 | 20 | 2.25 | 5.00 |
| 29 | 798 | Anesthetic Requirement Is Increased in Redheads | Liem EB. | Anesthesiology | 2004 | Free | USA | Anesthesia practise | RCT | 108 | 247 | 5.14 | 11.76 |
| 30 | 788 | Physiology of Thermoregulation | Kurz A. | Bailliere's Best Practice & Research. Clinical Anaesthesiology | 2008 | No | USA | Others | Review | 133 | 341 | 7.82 | 20.06 |
| 31 | 743 | The context of a noxious stimulus affects the pain it evokes | Moseley GL. | Pain (03043959) | 2007 | No | UK | Algology | Observational study | 101 | 210 | 5.61 | 11.67 |
| 32 | 736 | For nature cannot be fooled. Why we need to talk about fatigue | Farquhar M. | Anaesthesia | 2017 | Free | UK | HCWS | Editorial | 19 | 30 | 2.38 | 3.75 |
| 33 | 731 | Rates of opioid misuse. abuse. and addiction in chronic pain | Vowles KE. | Pain (03043959) | 2015 | No | USA | Algology | Review | 799 | 1360 | 79.90 | 136.00 |
| 34 | 720 | International Stakeholder Community of Pain Experts and Leaders Call for an Urgent Action on Forced Opioid Tapering | Darnall BD. | Pain Medicine | 2018 | Free | USA | Algology | Editorial | 83 | 132 | 11.86 | 18.86 |
| 35 | 706 | Sex differences in pain: a brief review of clinical and experimental findings | Bartley EJ. | BJA: The British Journal of Anaesthesia | 2013 | Free | USA | Algology | Review | 1198 | 2099 | 99.83 | 174.92 |
| 36 | 695 | Corticosteroids for Patients With Coronavirus Disease 2019 (COVID-19) With Different Disease Severity: A Meta-Analysis of Randomized Clinical Trials | Pasin L. | Journal of Cardiothoracic and Vascular Anesthesia | 2020 | Free | Italy | COVID 19 | Review | 53 | 104 | 10.60 | 20.80 |
| 37 | 692 | Long-lasting antinociceptive effects of green light in acute and chronic pain in rats | Ibrahim MM. | Pain (03043959) | 2016 | Free | USA | Algology | Experimental study | 69 | 104 | 7.67 | 11.56 |
| 38 | 681 | Music versus midazolam during preoperative nerve block placements: a prospective randomized controlled study | Graff V. | Regional Anesthesia & Pain Medicine | 2019 | No | USA | Egional Anesthesia | RCT | 38 | 71 | 6.33 | 11.83 |
| 39 | 674 | Global Warming Potential of Inhaled Anesthetics: application to clinical use | Ryan SM. | Anesthesia and analgesia | 2010 | Free | USA | Anesthesia practice | Observational study | 200 | 390 | 13.33 | 26.00 |
| 40 | 672 | Point-of-care lung ultrasound in patients with COVID-19 a narrative review | Smith MJ. | Anaesthesia | 2020 | Free | UK | COVID-19 | Review | 217 | 39 | 43.40 | 78.20 |
| 41 | 667 | The Impact of Massage Therapy on Function in Pain Populations—A Systematic Review and Meta-Analysis of Randomized Controlled Trials: Part I. Patients Experiencing Pain in the General Population | Crawford C. | Pain Medicine | 2016 | Free | UK | Algology | Review | 63 | 100 | 7.00 | 11.11 |
| 42 | 658 | The role of prior pain experience and expectancy in psychologically and physically induced pain | Bayer TL. | Pain (03043959) | 1998 | No | New Zealand | Algology | RCT | 28 | 70 | 1.04 | 2.59 |
| 43 | 649 | Opioids in chronic non-cancer pain: systematic review of efficacy and safety | Kalso E. | Pain (03043959) | 2004 | No | UK | Algology | Review | 901 | 1597 | 42.90 | 76.05 |
| 44 | 644 | Dark Skin Decreases the Accuracy of Pulse Oximeters at Low Oxygen Saturation: The Effects of Oximeter Probe Type and Gender | Feiner JR. | Anesthesia and analgesia | 2007 | Free | USA | Patient safety | RCT | 217 | 387 | 12.06 | 21.50 |
| 45 | 643 | Chronic pain as a symptom or a disease | Treede RD. | Pain (03043959) | 2019 | No | Germany | Algology | Review | 1354 | 2515 | 225.67 | 419.17 |
| 46 | 641 | TRALI – Definition. mechanisms. incidence and clinical relevance | Toy P. | Bailliere's Best Practice & Research. Clinical Anaesthesiology | 2007 | Free | USA | İntensive care | Review | 67 | 121 | 3.72 | 6.72 |
| 47 | 637 | Life Cycle Greenhouse Gas Emissions of Anesthetic Drugs | Sherman J. | Anesthesia and analgesia | 2012 | Free | USA | Anesthesia practice | Observational Study | 263 | 432 | 20.23 | 33.23 |
| 48 | 635 | Impact of vaccination by priority group on UK deaths. hospital admissions and intensive care admissions from COVID-19 | Cook TM. | Anaesthesia | 2021 | Free | UK | COVID-19 | Retrospective study | 48 | 84 | 12.00 | 21.00 |
| 49 | 632 | What we do when a COVID-19 patient needs an operation: operating room preparation and guidance | Ti LK. | Canadian Journal of Anesthesia/Journal canadien d'anesthésie | 2020 | Free | Singapore | COVID-19 | Editorial | 261 | 705 | 52.20 | 141.00 |
| 50 | 627 | A comprehensive review of opioid-induced hyperalgesia. | Lee M. | Pain Physician | 2011 | Free | USA | Algology | Review | 711 | 1434 | 50.79 | 102.43 |
| 51 | 622 | Risks to healthcare workers following tracheal intubation of patients with COVID?19: a prospective international multicentre cohort study | El-Boghdadly K. | Anaesthesia | 2020 | Free | UK | HCWS | Observational Study | 191 | 290 | 38.20 | 58.00 |
| 52 | 620 | Global Capnography Project (GCAP): implementation of capnography in Malawi – an international anaesthesia quality improvement project | Jooste R. | Anaesthesia | 2018 | Free | Malawi | Patient safetys | Observational study | 36 | 47 | 5.14 | 6.71 |
| 53 | 618 | The Effect of Low-Carbohydrate and Low-Fat Diets on Pain in Individuals with Knee Osteoarthritis | Strath LJ. | Pain Medicine | 2019 | Free | USA | Anesthesia practise | RCT | 37 | 62 | 6.17 | 10.33 |
| 54 | 617 | Pre- operative nutrition and the elective surgical patient: why. how and what? | Gillis C. | Anaesthesia | 2019 | Free | USA | Anesthesia practise | Review | 105 | 182 | 17.50 | 30.33 |
| 55 | 595 | Association of Multimodal Pain Management Strategies with Perioperative Outcomes and Resource Utilization | Memtsoudis SG. | Anesthesiology | 2018 | Free | USA | Algology | RCT | 127 | 198 | 18.14 | 28.29 |
| 56 | 580 | ASRA Pain Medicine consensus guidelines on the management of the perioperative patient on cannabis and cannabinoids | Shah S. | Regional Anesthesia & Pain Medicine | 2023 | Free | USA | Algology | Guideline | 24 | 38 | 12.00 | 19.00 |
| 57 | 569 | Molecular mechanisms of opioid receptor-dependent signaling and behavior. | Al-Hasani R. | Anesthesiology | 2011 | Free | USA | Algology | Review | 644 | 1242 | 46.00 | 88.71 |
| 58 | 559 | World Health Organization-World Federation of Societies of Anaesthesiologists (WHO-WFSA) International Standards for a Safe Practice of Anesthesia | Gelb AW. | Anesthesia and analgesia | 2018 | Free | USA | Patient safety | Guideline | 47 | 138 | 6.71 | 19.71 |
| 59 | 555 | Calibration of Contactless Pulse Oximetry | Verkruysse W. | Anesthesia and analgesia | 2016 | Free | Netherlands | Monitorization | RCT | 56 | 114 | 6.22 | 12.67 |
| 60 | 551 | Factors Related to Migraine Patients’ Decisions to Initiate Behavioral Migraine Treatment Following a Headache Specialist’s Recommendation: A Prospective Observational Study | Minen MT. | Pain Medicine | 2018 | Free | USA | Algology | Observational Study | 22 | 30 | 3.14 | 4.29 |
| 61 | 551 | The Impact of Massage Therapy on Function in Pain Populations—A Systematic Review and Meta-Analysis of Randomized Controlled Trials: Part II. Cancer Pain Populations | Boyd C. | Pain Medicine | 2016 | Free | USA | Algology | Review | 54 | 100 | 6.00 | 11.11 |
| 62 | 549 | Delayed Detection of Esophageal Intubation in Anesthesia Malpractice Claims | Honardar MR. | Anesthesia and analgesia | 2017 | Free | USA | Airway management | Retrospective study | 19 | 38 | 2.38 | 4.75 |
| 63 | 548 | Functional and Patient-Reported Outcomes in Symptomatic Lumbar Spinal Stenosis Following Percutaneous Decompression | Mekhail N. | Pain Practice | 2012 | No | USA | Algology | Retrospective | 32 | 57 | 2.46 | 4.38 |
| 64 | 548 | Upper Extremity Regional Anesthesia | Neal JM. | Regional Anesthesia & Pain Medicine | 2009 | Free | USA | Regional Anesthesia | Review | 214 | 439 | 13.38 | 27.44 |
| 65 | 535 | Lidocaine infusions for refractory chronic migraine: a retrospective analysis | Schwenk ES. | Regional Anesthesia & Pain Medicine | 2022 | No | USA | Algology | Retrospective | 6 | 6 | 2.00 | 2.00 |
| 66 | 526 | Efficacy of ginger for nausea and vomiting: a systematic review of randomized clinical trials | Ernst E. | BJA: The British Journal of Anaesthesia | 2000 | Free | UK | Anesthesia practise | Review | 232 | 731 | 0.80 | 1.56 |
| 67 | 526 | Occupational COVID-19 risk for anaesthesia and intensive care staff – low-risk specialties in a high?risk setting | Cook TM. | Anaesthesia | 2020 | Free | UK | COVID-19 | Editorial | 20 | 39 | 46.40 | 146.20 |
| 68 | 522 | Balanced Opioid-free Anesthesia with Dexmedetomidine versus Balanced Anesthesia with Remifentanil for Major or Intermediate Noncardiac Surgery. | Beloeil H. | Anesthesiology | 2021 | Free | France | Anesthesia practise | RCT | 121 | 171 | 40.00 | 55.00 |
| 69 | 522 | The analysis of 168 randomised controlled trials to test data integrity | Carlisle. JB. | Anaesthesia | 2012 | Free | USA | Other | Review | 160 | 220 | 9.31 | 13.15 |
| 70 | 517 | False individual patient data and zombie randomised controlled trials submitted to Anaesthesia | J B Carlisle. | Anaesthesia | 2020 | Free | USA | Side Other | RCT | 60 | 91 | 12.00 | 18.20 |
| 71 | 517 | Airborne transmission of severe acute respiratory syndrome coronavirus?2 to healthcare workers: a narrative review | Wilson NM. | Anaesthesia | 2020 | Free | Australia | COVID -19 | Review | 229 | 355 | 45.80 | 71.00 |
| 72 | 515 | Emotional awareness and expression therapy. cognitive behavioral therapy. and education for fibromyalgia | Lumley MA. | Pain (03043959) | 2017 | Free | USA | Algology | RCT | 130 | 232 | 16.25 | 29.00 |
| 73 | 515 | Outcomes for Extremely Premature Infants | Glass HC. | Anesthesia and analgesia | 2015 | Free | USA | Anesthesia practise | Review | 426 | 945 | 42.60 | 94.50 |
| 74 | 513 | Consensus guidelines for managing the airway in patients with COVID-19 | Cook TM. | Anaesthesia | 2020 | Free | UK | COVID-19 | Guideline | 620 | 1122 | 124.00 | 224.40 |
| 75 | 508 | Cohort Study of the Impact of High-dose Opioid Analgesics on Overdose Mortality | Dasgupta N. | Pain Medicine | 2015 | Free | USA | Algology | Observational Study | 235 | 383 | 23.50 | 38.30 |
| 76 | 505 | Measurement of airborne particle exposure during simulated tracheal intubation using various proposed aerosol containment devices during the COVID-19 pandemic | Simpson JP. | Anaesthesia | 2020 | Free | Australia | COVID-19 | RCT | 103 | 5 | 20.60 | 1.00 |
| 77 | 504 | User Design and Experience Preferences in a Novel Smartphone Application for Migraine Management: A Think Aloud Study of the RELAXaHEAD Application | Minen MT. | Pain Medicine | 2018 | Free | USA | Algology | Observational Study l | 22 | 33 | 3.14 | 4.71 |
| 78 | 502 | Quantitative evaluation of aerosol generation during manual facemask ventilation | Shrimpton AJ. | Anaesthesia | 2021 | Free | UK | HCWS | RCT | 12 | 19 | 3.00 | 4.75 |
| 79 | 502 | Human Abuse Potential of the New Opioid Analgesic Molecule NKTR-181 Compared with Oxycodone | Webster L. | Pain Medicine | 2017 | Free | USA | Algology | RCT | 25 | 30 | 3.13 | 3.75 |
| 80 | 501 | American Society for Enhanced Recovery and Perioperative Quality Initiative Joint Consensus Statement on Nutrition Screening and Therapy Within a Surgical Enhanced Recovery Pathway | Wischmeyer PE. | Anesthesia and analgesia | 2018 | Free | USA | Anesthesia practise | Review | 223 | 377 | 26.71 | 40.86 |
| 81 | 501 | A History of Being Prescribed Controlled Substances and Risk of Drug Overdose Death | Paulozzi LJ. | Pain Medicine | 2012 | Free | USA | HCWS | Retrospective study | 177 | 282 | 17.15 | 29.00 |
| 82 | 497 | Screening for Insomnia: An Observational Study Examining Sleep Disturbances. Headache Characteristics. and Psychiatric Symptoms in Patients Visiting a Headache Center. | Begasse de Dhaem O. | Pain Medicine | 2017 | Free | USA | Algology | Retrospective study | 2 | 8 | 0.25 | 1.00 |
| 83 | 497 | Dorsal root ganglion stimulation yielded higher treatment success rate for complex regional pain syndrome and causalgia at 3 and 12 months | Deer TR. | Pain (03043959) | 2016 | Free | USA | Algology | RCT | 346 | 512 | 38.44 | 56.89 |
| 84 | 495 | Preventing unrecognised oesophageal intubation: a consensus guideline from the Project for Universal Management of Airways and international airway societies* | Chrimes N. | Anaesthesia | 2022 | Free | USA (multicenter) | Airway manwgament | Guideline | 89 | 104 | 29.67 | 34.67 |
| 85 | 495 | Complications of Spinal Cord Stimulation and Peripheral Nerve Stimulation Techniques: A Review of the Literature | Eldabe S. | Pain Medicine | 2015 | Free | UK | Algology | Review | 212 | 339 | 21.20 | 33.90 |
| 86 | 488 | Goals of Chronic Pain Management | Henry SG. | Clinical journal of pain | 2017 | Free | USA | Algology | Survey | 60 | 93 | 2.00 | 2.63 |
| 87 | 488 | Burnout in Healthcare Workers: Prevalence. Impact and Preventative Strategies | De Hert S. | Local and Regional Anesthesia | 2020 | Free | Belgium | HCWS | Review | 197 | 584 | 39.40 | 116.80 |
| 88 | 488 | Adverse Events Associated With 10-kHz Dorsal Column Spinal Cord Stimulation | D'Souza RS. | Clinical journal of pain | 2022 | No | USA | Algology | Retrospective | 16 | 21 | 20.00 | 31.00 |
| 89 | 479 | Striving for Consensus on Approaches to Category 1 Testing of Abuse-Deterrent Formulations of Opioids: Discussions from the First Category 1 Focus Group Meeting | Cone EJ. | Pain Practice | 2016 | Free | USA | Algology | Review | 5 | 5 | 0.56 | 0.56 |
| 90 | 479 | Virtual reality for persistent pain: A new direction for behavioral pain management | Keefe FJ. | Pain (03043959) | 2012 | Free | USA | Algology | Editorial | 11 | 197 | 8.54 | 15.15 |
| 91 | 477 | Dramatic Cervical Spine Injury Secondary to Videolaryngoscopy in a Patient Suffering from Ankylosing Spondylitis | Epaud A. | Anesthesiology | 2021 | Free | France | Airway management | Case report | 14 | 19 | 3.50 | 4.75 |
| 92 | 471 | Intra-abdominal hypertension in cardiac surgery patients: a multicenter observational sub-study of the Accuryn registry | Khanna AK. | Journal of Clinical Monitoring and Computing | 2022 | Free | USA | Anesthesia practice | Observational Study | 13 | 15 | 3.00 | 7.33 |
| 93 | 471 | Delta-9-tetrahydrocannabinol decreases masticatory muscle sensitization in female rats through peripheral cannabinoid receptor activation | Wong H. | European Journal of Pain | 2017 | Free | Canada | Algology | Experimental study | 9 | 22 | 1.63 | 1.88 |
| 94 | 470 | Far From “Just a Poke” | McMurtry CM. | Clinical journal of pain | 2015 | Free | UK | Algology | Review | N/A | 396 | 0.00 | 39.60 |
| 95 | 468 | An Open-Label Study of Sufentanil Sublingual Tablet 30-Mcg in Patients with Postoperative Pain | Hutchins JL. | Pain Medicine | 2017 | Free | Canada | Algology | Review | 20 | 26 | 2.50 | 3.25 |
| 96 | 465 | Liability Associated with Obstetric Anesthesia | Davies JM. | Anesthesiology | 2009 | Free | USA | Anesthesia  practice | Retrospective study | 125 | 252 | 0.56 | 0.69 |
| 97 | 465 | Comparison of Remote Electrical Neuromodulation and Standard-Care Medications for Acute Treatment of Migraine in Adolescents: A Post Hoc Analysis. | Hershey AD. | Pain Medicine | 2021 | Free | USA | Algology | Observational Study | 9 | 11 | 31.25 | 63.00 |
| 98 | 460 | The SLUScore | Stapelfeldt WH. | Anesthesia and analgesia | 2017 | Free | USA | Patient safety | RCT | 55 | 82 | 6.88 | 10.25 |
| 99 | 459 | Opioid Analgesic Prescribing: Facts vs Assumptions | Rose ME. | Pain Medicine | 2017 | Free | USA | Algology | Review | 69 | 146 | 8.63 | 18.25 |
| 100 | 456 | HTX-011 reduced pain intensity and opioid consumption versus bupivacaine HCl in bunionectomy: phase III results from the randomized EPOCH 1 study. | Viscusi E. | Regional Anesthesia & Pain Medicine | 2019 | Free | USA | Algoloy | RCT | 31 | 49 | 5.17 | 8.17 |
| 101 | 455 | Avoidable intensive care unit resource use and costs of unvaccinated patients with COVID-19: a historical population-based cohort study | Bagshaw SM. | Canadian Journal of Anesthesia/Journal canadien d'anesthésie | 2022 | Free | Canada | COVID-19 | Retrospective study | 3 | 5 | 1.00 | 1.67 |
| 102 | 455 | Pre-operative respiratory optimisation: an expert review | Lumb AB. | Anaesthesia | 2019 | Free | UK | Aneshesia practise | Review | 27 | 69 | 4.50 | 11.50 |
| 103 | 452 | Optimisation of pre-operative anaemia in patients before elective major surgery – why. who. when and how? | Munting KE. | Anaesthesia | 2019 | Free | Netherlands | Anesthesia practise | Review | 84 | 140 | 14.00 | 23.33 |
| 104 | 452 | Factors Associated with Suicidal Ideation in Patients with Chronic Non-Cancer Pain | Racine M. | Pain Medicine | 2016 | Free | Canada | Anesthetics | Survey | 22 | 40 | 2.44 | 4.44 |
| 105 | 447 | Difficult Airway Society guidelines for awake tracheal intubation (ATI) in adults | Ahmad I. | Anaesthesia | 2019 | Free | UK | Airway management | Guideline | 222 | 375 | 37.00 | 62.50 |
| 106 | 447 | Development of a Risk Index for Serious Prescription Opioid-Induced Respiratory Depression or Overdose in Veterans’ Health Administration Patients | Zedler B. | Pain Medicine | 2015 | Free | USA | Algoly | Retrospective study | 108 | 156 | 10.80 | 15.60 |
| 107 | 444 | Point-of-care Ultrasound in Cardiac Arrest | Paul JA. | Anesthesiology | 2021 | Free | USA | Anesthesia practise | Review | 14 | 28 | 3.50 | 7.00 |
| 108 | 443 | Efficacy of biofeedback for migraine: A meta-analysis | Nestoriuc Y. | Pain (03043959) | 2006 | No | Germany | Algology | Editorial | 165 | 438 | 0.26 | 0.89 |
| 109 | 443 | Is Routine Use of a Face Mask Necessary in the Operating Room? | Sellden E. | Anesthesiology | 2010 | Free | Sweeden | Patient safty | Review | 5 | 17 | 11.00 | 29.20 |
| 110 | 442 | Acupuncture Is Theatrical Placebo | Colquhoun D. | Anesthesia and analgesia | 2013 | Free | USA | Algology | Editorial | 76 | 174 | 6.33 | 14.50 |
| 111 | 442 | The Accuracy of 6 Inexpensive Pulse Oximeters Not Cleared by the Food and Drug Administration | Lipnick MS. | Anesthesia and analgesia | 2016 | Free | USA | Monitorization | Observational Study | 57 | 105 | 6.33 | 11.67 |
| 112 | 440 | Consensus statement on measures to promote equitable authorship in the publication of research from international partnerships | Morton B. | Anaesthesia | 2021 | Free | USA | Other | Guideline | 87 | 136 | 21.75 | 34.00 |
| 113 | 438 | Sufentanil Sublingual Tablet 30 mcg for the Management of Pain Following Abdominal Surgery: A Randomized. Placebo-Controlled. Phase-3 Study | Minkowitz HS. | Pain Practice | 2017 | Free | USA | Algology | RCT | 25 | 38 | 3.13 | 4.75 |
| 114 | 434 | Scope and Nature of Pain- and Analgesia-Related Content of the United States Medical Licensing Examination (USMLE) | Fishman SM. | Pain Medicine | 2018 | Free | USA | Algology | Survey | 22 | 37 | 3.14 | 5.29 |
| 115 | 431 | Increasing placebo responses over time in U.S. clinical trials of neuropathic pain | Tuttle AH. | Pain (03043959) | 2015 | No | Canada | Algology | Review | 169 | 268 | 16.90 | 26.80 |
| 116 | 430 | Long-Term Consequences of Chronic Pain: Mounting Evidence for Pain as a Neurological Disease and Parallels with Other Chronic Disease States | Fine PG. | Pain Medicine | 2011 | Free | USA | Algology | Review | 191 | 384 | 13.64 | 27.43 |
| 117 | 428 | Recomendaciones de consenso respecto al soporte respiratorio no invasivo en el paciente adulto con insuficiencia respiratoria aguda secundaria a infección por SARS-CoV-2 Clinical consensus recommendations regarding non-invasive respiratory support in the adult patient with acute respiratory failure secondary to SARS-CoV-2 infection | Cinesi Gómez C. | Revista española de anestesiología y reanimación | 2020 | Free | Spain | COVID-19 | Guideline | 37 | 146 | 7.40 | 29.20 |
| 118 | 427 | Altered microbiome composition in individuals with fibromyalgia. | Minerbi A. | Pain (03043959) | 2019 | Free | Canada | Algology | RCT | 104 | 172 | 17.33 | 28.67 |
| 119 | 426 | Yesterday’s luxury – today’s necessity: end-tidal CO2 monitoring during conscious sedation | Kurrek MM. | Canadian Journal of Anesthesia/Journal canadien d'anesthésie | 2012 | Free | Canada | Monitorization | Editorial | 5 | 13 | 0.38 | 1.00 |
| 120 | 426 | A possible neural mechanism for photosensitivity in chronic pain | Martenson ME. | Pain (03043959) | 2016 | Free | USA | Algology | RCT | 39 | 62 | 0.89 | 2.00 |
| 121 | 426 | Neurodevelopmental outcomes after prenatal exposure to anaesthesia for maternal surgery: a propensity-score weighted bidirectional cohort study | Bleeser T. | Anaesthesia | 2022 | Free | Belgium | Anesthesia Practice | Retrospective study | 8 | 18 | 13.00 | 20.67 |
| 122 | 424 | 2022 American Society of Anesthesiologists Practice Guidelines for Management of the Difficult Airway* | Apfelbaum JL. | Anesthesiology | 2021 | Free | USA | Airway manegement | Review | 363 | 674 | 90.75 | 168.50 |
| 123 | 422 | Methotrexate: A Gold Standard for Treatment of Rheumatoid Arthritis | Shinde CG. | Journal of Pain & Palliative Care Pharmacotherapy | 2014 | No | India | Algology | Review | N/A | 99 | 0.00 | 9.00 |
| 124 | 419 | Using an animated patient avatar to improve perception of vital sign information by anaesthesia professionals | Tscholl DW. | BJA: The British Journal of Anaesthesia | 2018 | Free | Switzerland | Monitorization | RCT | 28 | 39 | 4.00 | 5.57 |
| 125 | 418 | Impact of COVID-19 pandemic on chronic pain management: Looking for the best way to deliver care | Puntillo F. | Bailliere's Best Practice & Research. Clinical Anaesthesiology | 2020 | Free | Italy | COVID-19 | Review | 70 | 117 | 14.00 | 23.40 |
| 126 | 416 | Situation awareness errors in anesthesia and critical care in 200 cases of a critical incident reporting system | Schulz CM. | BMC Anesthesiology | 2016 | Free | Germany | Patient safety | Retrospective study | 64 | 128 | 7.11 | 14.22 |
| 127 | 413 | Early prediction of acute kidney injury after transapical and transaortic aortic valve implantation with urinary G1 cell cycle arrest biomarkers | Dusse F. | BMC Anesthesiology | 2016 | Free | Germany | Anesthesia practicey | Observational Study | 25 | 75 | 2.78 | 8.33 |
| 128 | **409** | Economic Costs of Nonmedical Use of Prescription Opioids | Hansen RN. | Clinical journal of pain | 2011 | No | USA | Algology | Observational study | 131 | 290 | 33.14 | 52.71 |
| 129 | **409** | Guidelines for the management of tracheal intubation in critically ill adults | Higgs A. | BJA: The British Journal of Anaesthesia | 2017 | Free | UK | Airway manegament | Guideline | 464 | 738 | 16.38 | 36.25 |
| 130 | **408** | Topical capsaicin for pain management: therapeutic potential and mechanisms of action of the new high-concentration capsaicin 8% patch | Anand P. | BJA: The British Journal of Anaesthesia | 2011 | Free | UK | Algology | Review | 429 | 806 | 30.64 | 57.57 |
| 131 | **405** | Injury-prone: peripheral nerve injuries associated with prone positioning for COVID-19-related acute respiratory distress syndrome | Malik GR. | BJA: The British Journal of Anaesthesia | 2020 | Free | USA | COVID-19 | Editorial | 60 | 122 | 12.00 | 24.40 |
| 132 | **404** | Abstract PR438 (Abstract PR438: Validation Study of the Covariates Model for Target Controlled Infusion of Propofol) | Hawthorne. | Anesthesia and analgesia | 2016 | No | UK | Anesthesia practise | Observational Study | N/A | 2 | 0.00 | 0.11 |
| 133 | **404** | Abstract PR437 (Abstract PR437: Calculating the Keo for the Covariates Model for Target Controlled Infusion of Propofol) | Hawthorne. | Anesthesia and analgesia | 2016 | No | UK | Anesthesia practise | Observational Study | N/A | 1 | 0.00 | 0.22 |
| 134 | **403** | SEER Sonorheometry (SEER Sonorheometry: Listening to What the Clot Has to Say) | Sniecinski. | Anesthesia and analgesia | 2016 | Free | USA | Monitorization | Editorial | 5 | 7 | 0.56 | 0.78 |
| 135 | **402** | Perioperative Opioid Administration. | Shanthanna H. | Anesthesiology | 2020 | Free | Canada | Algology | Review | 109 | 158 | 21.80 | 31.60 |
| 136 | **399** | Trajectories and predictors of the long-term course of low back pain | Chen Y. | Pain (03043959) | 2017 | Free | UK | Algology | Observational Study | 83 | 127 | 1.75 | 2.88 |
| 137 | **399** | The impact of parental contact upon cortical noxious-related activity in human neonates | Jones L. | European Journal of Pain | 2020 | Free | Canada | Algology | Observational | 14 | 23 | 16.60 | 25.40 |
| 138 | **399** | Pre-operative optimisation of the surgical patient with diagnosed and undiagnosed diabetes: a practical review | Levy N. | Anaesthesia | 2019 | Free | UK | Anesthesia practise | Review | 31 | 60 | 5.17 | 10.00 |
| 139 | **397** | SEER Sonorheometry Versus Rotational Thromboelastometry in Large Volume Blood Loss Spine Surgery | Naik BI | Anesthesia and analgesia | 2016 | Free | USA | Monitorization | Observational Study | 26 | 31 | 2.89 | 3.44 |
| 140 | **396** | Comorbidity and utilization of medical services by pain patients receiving opioid medications: Data from an insurance claims database | Cicero TJ. | Pain (03043959) | 2009 | Free | USA | Algology | Observational Study | 91 | 140 | 5.69 | 8.75 |
| 141 | **396** | A Novel Device for the Evaluation of Hemostatic Function in Critical Care Settings | Ferrante EA. | Anesthesia and analgesia | 2016 | Free | USA | Monitorization | Observational Study | 52 | 76 | 5.78 | 8.44 |
| 142 | **393** | A Comparison of a New Ultrasound-Based Whole Blood Viscoelastic Test (SEER Sonorheometry) Versus Thromboelastography in Cardiac Surgery | Reynolds PS. | Anesthesia and analgesia | 2016 | Free | USA | Anesthesia practice | Observational Study | 29 | 40 | 3.22 | 4.44 |
| 143 | **391** | The aerosol box for intubation in coronavirus disease 2019 patients: an in-situ simulation crossover study | Begley JL. | Anaesthesia | 2020 | Free | Australia | COVID-19 | Experimental study | 160 | 276 | 32.00 | 55.20 |
| 144 | **389** | The Effect of Medicinal Cannabis on Pain and Quality-of-Life Outcomes in Chronic Pain | Haroutounian S. | Clinical journal of pain | 2016 | No | Israel | Algology | Review | 178 | 281 | 19.78 | 31.22 |
| 145 | **389** | Sex-Based Differences in Pain Perception and Treatment | Paller CJ. | Pain Medicine | 2009 | Free | USA | Algology | Observational Study | 260 | 515 | 16.25 | 32.19 |
| 146 | **388** | Incidence of Spontaneous Resorption of Lumbar Disc Herniation: A Meta-Analysis. | Zhong M. | Pain Physician | 2017 | Free | China | Algology | Review | 56 | 144 | 7.00 | 18.00 |
| 147 | **386** | Combining Guided Intervention of Education and Relaxation (GIER) with Remote Electrical Neuromodulation (REN) in the Acute Treatment of Migraine. | Buse DC. | Pain Medicine | 2022 | Free | USA | Algology | O Observational Study | 6 | 10 | 2.00 | 3.33 |
| 148 | **385** | Placenta accreta and anesthesia: A multidisciplinary approach | Khokhar RS. | Saudi Journal of Anaesthesia | 2016 | Free | Saudi Arabia | Anesthesia practice | Case report | 7 | 11 | 0.78 | 1.22 |
| 149 | 384 | Effects of testosterone replacement in men with opioid-induced androgen deficiency | Basaria S. | Pain (03043959) | 2015 | Free | USA | Anesthesia practice | RCT | 81 | 128 | 8.10 | 12.80 |
| 150 | 382 | Psychological factors. prehabilitation and surgical outcomes: evidence and future directions | Levett DZH. | Anaesthesia | 2019 | Free | UK | Anesthesai practise | Review | 126 | 216 | 21.00 | 36.00 |
| 151 | 381 | Cannabis and cannabinoids for the treatment of people with chronic noncancer pain conditions | Black N. | Pain (03043959) | 2018 | Free | Australia | Algology | Review | 242 | 443 | 34.57 | 63.29 |
| 152 | 380 | SARS-CoV-2 infection and venous thromboembolism after surgery: an international prospective cohort study | COVIDSurg Collaborative; GlobalSurg Collaborative | Anaesthesia | 2021 | Free | UK | COVID-19 | Observational Study | 63 | 70 | 15.75 | 17.50 |
| 153 | 378 | Interactions between cannabinoid and opioid receptors in a mouse model of diabetic neuropathy | Toniolo EF. | Pain (03043959) | 2021 | Free | Brazil | Anesthesia practice | Experimental Study | 3 | 10 | 0.75 | 2.50 |
| 154 | 378 | Tablet-based Interactive Distraction (TBID) vs oral midazolam to minimize perioperative anxiety in pediatric patients: a noninferiority randomized trial | Seiden SC. | Paediatric Anaesthesia | 2014 | No | USA | Anesthesai practise | RCT | 52 | 104 | 4.73 | 9.45 |
| 155 | 377 | 2023 American Society of Anesthesiologists Practice Guidelines for Monitoring and Antagonism of Neuromuscular Blockade: A Report by the American Society of Anesthesiologists Task Force on Neuromuscular Blockade | Thilen SR. | Anesthesiology | 2023 | Free | USA | Monitorization | Guideline | 64 | 115 | 32.00 | 57.50 |
| 156 | 375 | Cancelled operations: a 7-day cohort study of planned adult inpatient surgery in 245 UK National Health Service hospitals | Wong DJN. | BJA: The British Journal of Anaesthesia | 2018 | Free | UK | Patient safety | Observational Study | 77 | 96 | 11.00 | 13.71 |
| 157 | 374 | Attenuation of early phase inflammation by cannabidiol prevents pain and nerve damage in rat osteoarthritis | Philpott HT. | Pain (03043959) | 2017 | Free | Canada | Algology | Experimental study | 163 | 314 | 20.38 | 39.25 |
| 158 | 373 | Timing of elective surgery and risk assessment after SARS-CoV-2 infection: an update | El-Boghdadly K. | Anaesthesia | 2022 | Free | UK | COVID-19 | Guideline | 44 | 71 | 14.67 | 23.67 |
| 159 | 372 | Real-world study of intranasal ketamine for use in patients with refractory chronic migraine: a retrospective analysis | Yuan H. | Regional Anesthesia & Pain Medicine | 2023 | Free | USA | Algology | Retrospective study | 1 | 2 | 0.50 | 1.00 |
| 160 | 371 | Predicting preschool pain-related anticipatory distress | Racine NM. | Pain (03043959) | 2016 | No | Canada | Algoloy | RCT | 15 | 36 | 1.67 | 4.00 |
| 161 | 370 | An efficient randomised. placebo-controlled clinical trial with the irreversible fatty acid amide hydrolase-1 inhibitor PF-04457845. which modulates endocannabinoids but fails to induce effective analgesia in patients with pain due to osteoarthritis of the knee | Huggins JP. | Pain (03043959) | 2012 | No | UK | Algology | RCT | 310 | 418 | 23.85 | 32.15 |
| 162 | 369 | Intubation and Ventilation amid the COVID-19 Outbreak | Meng L. | Anesthesiology | 2020 | Free | USA | COVID-19 | Review | 360 | 782 | 72.00 | 156.40 |
| 163 | 367 | Video Laryngoscopy-Guided Transesophageal Echocardiography Probe Insertion: A Worthy Approach to Consider | Kumar N. | Journal of Cardiothoracic and Vascular Anesthesia | 2022 | Free | USA | Airway management | Editorial | 1 | 1 | 1.00 | 2.67 |
| 164 | 367 | Anxiolytic and sedative polypharmacy among US opioid users: a cross-sectional study | Sites BD. | Regional Anesthesia & Pain Medicine | 2022 | No | USA | Algology | Survey | 1 | 1 | 0.33 | 0.33 |
| 165 | 367 | Unusual Access to Airway with Transorbital Intubation | dos Reis Falcão LF. | Anesthesiology | 2014 | Free | Brasil | Airway managemenr | Case report | 3 | 8 | 0.09 | 0.09 |
| 166 | 366 | Opioid Induced Hyperalgesia | Yi P. | Pain Medicine | 2015 | Free | USA | Algology | Review | 113 | 221 | 23.20 | 40.90 |
| 167 | 366 | Hyperchloremia After Noncardiac Surgery Is Independently Associated with Increased Morbidity and Mortality | McCluskey SA. | Anesthesia and analgesia | 2013 | Free | CANADA | Anesthesia practice | Retrospective study | 232 | 409 | 9.42 | 18.42 |
| 168 | 363 | Indifference or hypersensitivity? Solving the riddle of the pain profile in individuals with autism | Hoffman T. | Pain (03043959) | 2022 | No | Israel | Algology | RCT | 4 | 7 | 1.33 | 2.33 |
| 169 | 361 | An Experimental Test of the Effectiveness of Unsolicited Reporting by a Prescription Drug Monitoring Program in Reducing Inappropriate Acquisition of Opioids. | McDonald DC. | Pain Medicine | 2018 | Free | USA | Algology | Experimental Study | 12 | 18 | 1.71 | 2.57 |
| 170 | 361 | Preparing previously COVID-19-positive patients for elective surgery: a framework for preoperative evaluation | Bui N. Coetzer M. | Perioperative Medicine | 2021 | Free | USA | COVID-19 | RCT | 30 | 58 | 7.50 | 14.50 |
| 171 | 361 | Opioid pharmacology. | Trescot AM. | Pain Physician | 2008 | Free | USA | Algology | Review | 581 | 1359 | 34.18 | 79.94 |
| 172 | 360 | Acute Low Back Pain? Do Not Blame the Weather-A Case-Crossover Study. | Beilken K. | Pain Medicine | 2016 | Free | Australia | Algology | RCT | 23 | 22 | 2.56 | 2.44 |
| 173 | 359 | Continuous hemoglobin and plethysmography variability index monitoring can modify blood transfusion practice and is associated with lower mortality | Cros J. | Journal of Clinical Monitoring and Computing | 2019 | No | France | Monitorization | Observational Study | 6 | 37 | 1.00 | 6.17 |
| 174 | 355 | Managing patients with chronic pain during the COVID-19 outbreak: considerations for the rapid introduction of remotely supported (eHealth) pain management services | Eccleston C. | Pain (03043959) | 2020 | Free | UK | COVID-19 | Review | 315 | 500 | 63.00 | 100.00 |
| 175 | 354 | A cross-sectional study of immune seroconversion to SARS-CoV-2 in frontline maternity health professionals | Bampoe S. | Anaesthesia | 2020 | Free | UK | COVID-19 | Observational Study | 15 | 26 | 3.00 | 5.20 |
| 176 | 354 | A national survey of the effects of fatigue on trainees in anaesthesia in the UK | McClelland L. | Anaesthesia | 2017 | Free | UK | HCWS | Survey | 91 | 111 | 11.38 | 13.88 |
| 177 | 353 | Segregation for reduction of regulated medical waste in the operating room: a case report | Shinn HK. | Korean Journal of Anesthesiology | 2016 | Free | Korea | HCWS | Case report | 20 | 45 | 2.22 | 5.00 |
| 178 | 353 | Preoperative Anxiety and Emergence Delirium and Postoperative Maladaptive Behaviors | Kain ZN. | Anesthesia and analgesia | 2004 | Free | USA | Anesthesia practice | RCT | 403 | 874 | 19.19 | 41.62 |
| 179 | 350 | Evaluation of Perioperative Medication Errors and Adverse Drug Events | Nanji KC. | Anesthesiology | 2016 | Free | USA | Anesthesia practice | Case report | 174 | 369 | 19.33 | 41.00 |
| 180 | 350 | Pre-operative cardiac optimisation: a directed review | Lee LKK. | Anaesthesia | 2019 | Free | China | Anesthesia practise | Review | 22 | 51 | 3.67 | 8.50 |
| 181 | 349 | Respiratory Depression with Tramadol in a Patient with Renal Impairment and CYP2D6 Gene Duplication | Stamer UM. | Anesthesia and analgesia | 2008 | Free | Germany | Anesthesia practice | Case report | 120 | 216 | 7.06 | 12.71 |
| 182 | 347 | Health Implications of Disrupted Circadian Rhythms and the Potential for Daylight as Therapy | Brainard J. | Anesthesiology | 2015 | Free | Germany | HCWS | Review | 42 | 106 | 2.40 | 3.70 |
| 183 | 347 | Self-reported Race/Ethnicity and Intraoperative Occult Hypoxemia: A Retrospective Cohort Study | Burnett GW. | Anesthesiology | 2022 | Free | USA | Monitoriztion | Retrospective study | 24 | 37 | 14.00 | 35.33 |
| 184 | 345 | Guidelines for the safe practice of total intravenous anaesthesia (TIVA) | Nimmo AF. | Anaesthesia | 2019 | Free | UK | Anesthesia practice | Guideline | 153 | 244 | 25.50 | 40.67 |
| 185 | 344 | Chronic Hiccups | Kohse EK. | Anesthesia and analgesia | 2017 | Free | Germany. | Anesthesia practise | Review | 33 | 70 | 4.13 | 8.75 |
| 186 | 343 | Post-acute sensory neurological sequelae in patients with severe acute respiratory syndrome coronavirus 2 infection: the COVID-PN observational cohort study | Odozor CU. | Pain (03043959) | 2022 | No | USA | COVID-19 | Observational study | 7 | 11 | 2.33 | 3.67 |
| 187 | 340 | ASRA Pain Medicine consensus guidelines on the management of the perioperative patient on cannabis and cannabinoids: an infographic | Shah S. | Regional Anesthesia & Pain Medicine | 2023 | Free | USA | Algology | Guideline | 1 | 2 | 0.50 | 1.00 |
| 188 | 340 | Reframe the pain: Divided attention and positive memory reframing to reduce needle pain and distress in children—A feasibility randomized controlled trial | Braithwaite FA. | European Journal of Pain | 2022 | Free | Australia | Anesthesia practice | RCT | 4 | 6 | 5.67 | 8.67 |
| 189 | 340 | Pregnancy and Labor Epidural Effects on Gastric Emptying: A Prospective Comparative Study | Bouvet L. | Anesthesiology | 2022 | Free | France | Anesthesia practice | Observational Study | 17 | 26 | 37.33 | 71.33 |
| 190 | 340 | Increased Sensitivity to Thermal Pain and Reduced Subcutaneous Lidocaine Efficacy in Redheads | Liem EB. | Anesthesiology | 2005 | Free | USA | Algology | RCT | 112 | 214 | 0.20 | 0.30 |
| 191 | 338 | Chronic use of opioid analgesics in non-malignant pain: Report of 38 cases | Portenoy RK. | Pain (03043959) | 1986 | No | USA | Algology | Case report | 596 | 1251 | 15.28 | 32.08 |
| 192 | 338 | Multimodal General Anesthesia | Brown EN. | Anesthesia and analgesia | 2018 | Free | USA | Anesthesia practise | Review | 224 | 516 | 32.00 | 73.71 |
| 193 | 336 | The role of fit testing N95/FFP2/FFP3 masks: a narrative review | Regli A. | Anaesthesia | 2020 | Free | Australia. | HCWS | Review | 60 | 116 | 12.00 | 23.20 |
| 194 | 334 | Opioid dose and pain effects of an online pain self-management program to augment usual care in adults with chronic pain: a multisite randomized clinical trial | Wilson M. | Pain (03043959) | 2022 | Free | USA | Algology | RCT | 2 | 5 | 0.67 | 1.67 |
| 195 | 333 | Peri-operative optimisation of elderly and frail patients: a narrative review | Chan SP. | Anaesthesia | 2019 | Free | China | Anesthesia practise | Review | 68 | 123 | 11.33 | 20.50 |
| 196 | 331 | Patient outcomes after opioid dose reduction among patients with chronic opioid therapy | Hallvik SE. | Pain (03043959) | 2021 | Free | USA | Algology | Retrospective study | 30 | 43 | 7.50 | 10.75 |
| 197 | 330 | Challenges with Implementing the Centers for Disease Control and Prevention Opioid Guideline: A Consensus Panel Report | Kroenke K. | Pain Medicine | 2019 | Free | USA | Algology | Review | 135 | 206 | 22.50 | 34.33 |
| 198 | 327 | Pharmacokinetic Properties of a Sufentanil Sublingual Tablet Intended to Treat Acute Pain | Fisher DM. | Anesthesiology | 2018 | Free | USA | Algology | RCT | 19 | 33 | 2.57 | 4.43 |
| 199 | 327 | Conditioned open-label placebo for opioid reduction after spine surgery: a randomized controlled trial | Flowers KM. | Pain (03043959) | 2021 | Free | USA | Algology | RCT | 18 | 31 | 4.75 | 8.25 |
| 200 | 323 | A randomized. double-blind. placebo-controlled study of daily cannabidiol for the treatment of canine osteoarthritis pain. | Verrico CD. | Pain (03043959) | 2020 | Free | USA | COVAlgology | RCT | 90 | 166 | 18.00 | 33.20 |
| 201 | 320 | Nutrition deficit during intensive care stay: incidence. predisposing factors and outcomes | Nurkkala JP. | Minerva Anestesiologica | 2020 | Free | Finland | Anethesia practise | Retrospective study | 5 | 9 | 1.00 | 1.80 |
| 202 | 320 | Nitric Oxide Story. | Zapol WM. | Anesthesiology | 2019 | Free | USA | Algology | Review | 10 | 16 | 1.67 | 2.67 |
| 203 | 319 | Cannabinoids. cannabis. and cannabis-based medicine for pain management: a systematic review of randomised controlled trials | Fisher E. | Pain (03043959) | 2020 | Free | UK | Algology | RCT | 92 | 174 | 18.40 | 34.80 |
| 204 | 318 | Comparison of Intraarticular Pulsed Radiofrequency and Intraarticular Corticosteroid Injection for Management of Cervical Facet Joint Pain. | Lim JW. | Pain Physician | 2017 | Free | Republic of Korea | Algology | Observational | 9 | 23 | 1.13 | 2.88 |
| 205 | 317 | Perioperative Guidelines on Antiplatelet and Anticoagulant Agents: 2022 Update | Michael Moster. | Current Anesthesiology Reports | 2022 | Free | Switzerland | Anesthesia practise | Review | 20 | 36 | 6.67 | 12.00 |
| 206 | 316 | Short-term infusion of the mu-opioid agonist remifentanil in humans causes hyperalgesia during withdrawal | Angst MS. | Pain (03043959) | 2003 | No | USA/ Germany | Algology | RCT | 320 | 595 | 14.55 | 27.05 |
| 207 | 314 | Perioperative COVID-19 Defense: An Evidence-Based Approach for Optimization of Infection Control and Operating Room Management | Dexter F. | Anesthesia and analgesia | 2020 | Free | USA | COVID-19 | Review | 150 | 371 | 30.00 | 74.20 |
| 208 | 313 | American Society of Regional Anesthesia and Pain Medicine expert panel recommendations on point-of-care ultrasound education and training for regional anesthesiologists and pain physicians—part I: clinical indications | Haskins SC. | Regional Anesthesia & Pain Medicine | 2021 | Free | USA | Regional anesthesia | Review | 23 | 48 | 5.75 | 12.00 |
| 209 | 312 | Management of haematoma after thyroid surgery: systematic review and multidisciplinary consensus guidelines from the Difficult Airway Society. the British Association of Endocrine and Thyroid Surgeons and the British Association of Otorhinolaryngology. Head and Neck Surgery | Iliff HA. | Anaesthesia | 2022 | Free | UK | Airway Management | Guideline | 23 | 45 | 7.67 | 15.00 |
| 210 | 312 | Causes and Consequences of Inadequate Management of Acute Pain | Sinatra R | Pain Medicine | 2010 | Free | USA | Algology | Review | 345 | 693 | 23.00 | 46.20 |
| 211 | 311 | A tale of 2 ADFs | Cicero TJ. | Pain (03043959) | 2016 | No | USA | Anesthesia practisey | Survey | 25 | 31 | 2.67 | 3.78 |
| 212 | 311 | Development and validation of a multivariable prediction model for early prediction of chronic postsurgical pain in adults: a prospective cohort study | van Driel MEC. | BJA: The British Journal of Anaesthesia | 2022 | Free | The Netherlands | Algology | Observational Study | 24 | 34 | 22.00 | 34.33 |
| 213 | 311 | Intraoperative Use of Remifentanil for TIVA: Postoperative Pain. Acute Tolerance. and Opioid-Induced Hyperalgesia | Angst MS. | Journal of Cardiothoracic and Vascular Anesthesia | 2015 | No | USA | Anesthesia practice | Observational study | 66 | 103 | 2.50 | 3.10 |
| 214 | 310 | Chronic exposure to insufficient sleep alters processes of pain habituation and sensitization | Simpson NS. | Pain (03043959) | 2022 | Free | USA | Algology | RCT | 74 | 124 | 8.33 | 13.33 |
| 215 | 310 | Exposure to General Anesthesia for Cesarean Delivery and Odds of Severe Postpartum Depression Requiring Hospitalization | Guglielminotti J. | Anesthesia and analgesia | 2020 | Free | USA | Anesthesia practice | Retrospective study | 25 | 40 | 14.80 | 24.80 |
| 216 | 310 | Guidelines for the management of glucocorticoids during the peri-operative period for patients with adrenal insufficiency | Woodcock T. | Anaesthesia | 2022 | Free | UK | Anesthesia practise | Review | 73 | 128 | 24.33 | 42.67 |
| 217 | 309 | Associations of nadir haemoglobin level and red blood cell transfusion with mortality and length of stay in surgical specialties: a retrospective cohort study | Trentino KM. | Anaesthesia | 2019 | Free | Australia | Anesthesia practice | Retrospective study | 16 | 30 | 2.67 | 5.00 |
| 218 | 307 | Point-of-Care Ultrasound (POCUS) for the Cardiothoracic Anesthesiologist | Kalagara H. | Journal of Cardiothoracic and Vascular Anesthesia | 2022 | Free | USA | Anesthesia | Review | 21 | 39 | 7.00 | 13.00 |
| 219 |  | Prevalence of Dementia 7.5 Years after Coronary Artery Bypass Graft Surgery | Evered LA. | Anesthesiology | 2016 | Free | Australia | Anesthesia practics | Observational | 105 | 176 | 11.67 | 19.56 |
| 220 | 305 | Use of tranexamic acid in major trauma: a sex-disaggregated analysis of the Clinical Randomisation of an Antifibrinolytic in Significant Haemorrhage (CRASH-2 and CRASH-3) trials and UK trauma registry (Trauma and Audit Research Network) data | Nutbeam T. | BJA: The British Journal of Anaesthesia | 2022 | Free | USA | Trauma | RCT | 16 | 27 | 5.33 | 9.00 |
| 221 | 305 | Simplified Algorithm for Evaluation of Perioperative Hypoxia and Hypotension (SALVATION): A Practical Echo-guided Approach Proposal | Fatima H. | Journal of Cardiothoracic and Vascular Anesthesia | 2021 | Free | USA | Patient safety | Review | 4 | 6 | 1.00 | 1.50 |
| 222 | 305 | Interventions for Individuals With High Levels of Needle Fear | McMurtry CM. | Clinical journal of pain | 2015 | Free | Canada | Algology | Retrospective study | 55 | 91 | 2.00 | 2.20 |
| 223 | 304 | Association between widespread pain and dementia. Alzheimer’s disease and stroke: a cohort study from the Framingham Heart Study | Wang K. | Regional Anesthesia & Pain Medicine | 2021 | Free | China | Algology | Review | 20 | 22 | 13.75 | 22.75 |
| 224 | 304 | The Italian coronavirus disease 2019 outbreak: recommendations from clinical practice | Sorbello M. | Anaesthesia | 2020 | Free | Italy | COVID-19 | Guideline | 236 | 359 | 47.20 | 71.80 |
| 225 | 303 | A classification of chronic pain for ICD-11 | Treede RD. | Pain (03043959) | 2015 | Free | Germany | Algology | Review | 1557 | 3012 | 0.00 | 301.20 |
| 226 | 302 | Opioid complications and side effects. | Benyamin R. | Pain Physician | 2008 | Free | USA | Anesthesia practis | Review | 1467 | 2602 | 86.29 | 153.06 |
| 227 | 302 | The Effect of Deep and Slow Breathing on Pain Perception. Autonomic Activity. and Mood Processing—An Experimental Study | Busch V. | Pain Medicine | 2012 | Free | Germany | Algology | Review | 129 | 346 | 9.92 | 26.62 |
| 228 | 298 | Consensus Guidelines on the Use of Intravenous Ketamine Infusions for Chronic Pain From the American Society of Regional Anesthesia and Pain Medicine. the American Academy of Pain Medicine. and the American Society of Anesthesiologists | Cohen SP. | Regional Anesthesia & Pain Medicine | 2018 | Free | Canada | Ketamine | Review | 166 | 362 | 23.71 | 51.71 |
| 229 | 298 | “Living Well with Chronic Pain”: Integrative Pain Management via Shared Medical Appointments | Znidarsic J. | Pain Medicine | 2021 | Free | USA | Algology | Retrospective study | 11 | 23 | 2.75 | 5.75 |
| 230 | 296 | Modelling the economic constraints and consequences of anaesthesia associate expansion in the UK National Health Service: a narrative review | Hanmer SB. | BJA: The British Journal of Anaesthesia | 2024 | Free | UK | HCWS | Review | N/A | 7 | 7.00 | 7.00 |
| 231 | 296 | How to write a research protocol | Rout CC | Southern African Journal of Anaesthesia and Analgesia | 2016 | Free | South Africa | Others | Review | 4 | 17 | 0.44 | 1.89 |
| 232 | 296 | Opioid-Induced Constipation Survey in Patients with Chronic Noncancer Pain | Rauck RL. | Pain Practice | 2016 | No | USA | Anesthesia practise | Survey | 23 | 38 | 5.33 | 9.56 |
| 233 | 295 | Society of Cardiovascular Anesthesiologists/European Association of Cardiothoracic Anaesthetists Practice Advisory for the Management of Perioperative Atrial Fibrillation in Patients Undergoing Cardiac Surgery | O'Brien B. | Journal of Cardiothoracic and Vascular Anesthesia | 2019 | No | UK | Anesthesia practice | Review | 48 | 86 | 3.83 | 6.33 |
| 234 | 295 | Effect of a repeated verbal reminder of orientation on emergence agitation after general anaesthesia for minimally invasive abdominal surgery: a randomised controlled trial | Lee S. | BJA: The British Journal of Anaesthesia | 2023 | Free | South Korea | Algolnesthesia practice | RCT | 4 | 6 | 8.00 | 13.00 |
| 235 | 294 | Effect of diagnostic labelling on management intentions for non-specific low back pain: A randomized scenario -based experiment | O'Keeffe M. | European Journal of Pain | 2022 | Free | Australia | Algology | RCT | 16 | 26 | 1.33 | 2.00 |
| 236 | 294 | Fibromyalgia predicts increased odds of pain-related addiction exacerbation among individuals with pain and opioid use disorder | Hall OT. | Pain (03043959) | 2023 | Free | USA | Algology | Observational study | 5 | 10 | 2.50 | 5.00 |
| 237 | 291 | Current Concepts in the Management of Postoperative Nausea and Vomiting | Chatterjee S. | Anesthesiology Research & Practice | 2011 | Free | India | Anesthesia practise | Review | 91 | 289 | 6.50 | 20.64 |
| 238 | 290 | Developing a specialty: J.S. Lundy's three major contributions to anesthesiology | Ellis TA 2nd. | Journal of Clinical Anesthesia | 2004 | No | USA | Other | Editorial | 9 | 18 | 0.43 | 0.86 |
| 239 | 289 | Implementing human factors in anaesthesia: guidance for clinicians. departments and hospitals | Kelly FE. | Anaesthesia | 2023 | Free | UK | Patient safty | Guideline | 33 | 46 | 16.50 | 23.00 |
| 240 | 288 | Mortality in patients admitted to intensive care with COVID-19: an updated systematic review and meta?analysis of observational studies | Armstrong RA. | Anaesthesia | 2021 | Free | UK | COVID-19 | Review | 105 | 169 | 7.75 | 10.50 |
| 241 | 288 | Effects of pre-operative isolation on postoperative pulmonary complications after elective surgery: an international prospective cohort study | COVIDSurg Collaborative; GlobalSurg Collaborative. | Anaesthesia | 2021 | Free | UK | Anesthesia practice | Retrospective | 31 | 42 | 26.25 | 42.25 |
| 242 | 288 | Risk Stratification Index 3.0. a Broad Set of Models for Predicting Adverse Events during and after Hospital Admission | Greenwald S. | Anesthesiology | 2022 | Free | USA | Patient Safety | Review | 9 | 14 | 3.00 | 4.67 |
| 243 | 287 | Drug Interactions: Volatile Anesthetics and Opioids | Glass PS. | Journal of Clinical Anesthesia | 1997 | No | USA | Anesthesai Practise | Review | 39 | 110 | 1.39 | 3.93 |
| 244 | 286 | Pocket pain. does location matter: a single-centre retrospective study of patients implanted with a spinal cord stimulator | Baranidharan G. | Regional Anesthesia & Pain Medicine | 2020 | No | UK | Algology | Retrospective | 10 | 17 | 2.00 | 3.40 |
| 245 | 283 | Perioperative Medication Management | Wanderer J. P. | Anesthesiology | 2017 | Free | USA | Anesthesia practice | Editorial | N/A | 5 | 0.00 | 0.63 |
| 246 | 283 | Control of cardiac function | Charlesworth M. | Anaesthesia and Intensive Care Medicine | 2018 | Free | UK | Others | Review | 0 | 0 | 1.86 | 2.86 |
| 247 | 283 | Connected consciousness after tracheal intubation in young adults: an international multicentre cohort study | Lennertz R. | BJA: The British Journal of Anaesthesia | 2022 | Free | USA | Airway management | Observational study | 13 | 20 | 0.00 | 0.00 |
| 248 | 282 | Enhanced mindfulness-based stress reduction in episodic migraine: a randomized clinical trial with magnetic resonance imaging outcomes | Seminowicz DA. | Pain (03043959) | 2020 | Free | USA | Algology | RCT | 59 | 84 | 1.60 | 2.00 |
| 249 | 282 | New. long-term opioid use after lung cancer surgery is associated with reduced 2-year survival: a retrospective population-based cohort study in South Korea | Oh TK. | Regional Anesthesia & Pain Medicine | 2022 | Free | South Korea | Algology | Retrospective study | 8 | 10 | 19.67 | 28.00 |
| 250 | 281 | Fatal Vitamin C-associated Acute Renal Failure | McHugh GJ. | Anaesthesia and Intensive Care | 2008 | Free | New Zealand | Algology | Case rreport | 23 | 48 | 1.35 | 2.82 |
| 251 | 280 | The Pulse Oximeter Is Amazing. but Not Perfect | Bickler P. | Anesthesiology | 2022 | Free | USA | Monitorization | Editorial | 3 | 11 | 1.00 | 3.67 |
| 252 | 280 | Ketamine | Craven R. | Anaesthesia | 2007 | Free | UK | Anethesia Practise | Review | 170 | 291 | 2.28 | 3.67 |
| 253 | 280 | Guidelines for the peri-operative care of people with dementia | White S. | Anaesthesia | 2019 | Free | USA | Anesthesia practice | Guideline | 41 | 66 | 28.33 | 48.50 |
| 254 | 279 | Efficacy of the Buzzy Device for Pain Management During Needle-related Procedures | Ballard A. | Clinical journal of pain | 2019 | Free | Canada | Anesthesai practise | RCT | 40 | 86 | 6.67 | 14.33 |
| 255 | 277 | Recommendations for standards of monitoring during anaesthesia and recovery 2021 | Klein AA. | Anaesthesia | 2021 | Free | UK | Monitorization | Guideline | 145 | 207 | 36.25 | 51.75 |
| 256 | 275 | Perioperative Use of Gabapentinoids for the Management of Postoperative Acute Pain: A Systematic Review and Meta-analysis. | Verret M. | Anesthesiology | 2020 | Free | Canada | Algology | Review | 9 | 305 | 1.80 | 61.00 |
| 257 | 274 | Effect of awake prone positioning in COVID-19 patients- A systematic review | Anand S. | Trends in Anaesthesia and Critical Care | 2020 | Free | India | COVID-19 | Review | 6 | 29 | 1.20 | 5.80 |
| 258 | 274 | Guidelines for developing. translating. and validating a questionnaire in perioperative and pain medicine | Tsang S. | Saudi Journal of Anaesthesia | 2017 | Free | USA | Algology | Review | 721 | 1482 | 90.13 | 185.25 |
| 259 | 273 | Real-World Massage Therapy Produces Meaningful Effectiveness Signal for Primary Care Patients with Chronic Low Back Pain: Results of a Repeated Measures Cohort Study. | Elder WG. | Pain Medicine | 2017 | Free | USA | Algology | Retrospective study | 5 | 34 | 0.63 | 4.25 |
| 260 | 273 | Regional nerve blockade for early analgesic management of elderly patients with hip fracture – a narrative review | Scurrah A. | Anaesthesia | 2017 | Free | Australia | Regional anesthesia | Review | 67 | 111 | 7.63 | 9.63 |
| 261 | 273 | Opioid Oversupply After Joint and Spine Surgery | Bicket MC. | Anesthesia and analgesia | 2019 | Free | USA | Algology | Observational study | 61 | 77 | 11.17 | 18.50 |
| 262 | 272 | The pharmacokinetics. efficacy. and safety of a novel selective?dose cannabis inhaler in patients with chronic pain: A randomized. double?blinded. placebo?controlled trial | Almog S. | European Journal of Pain | 2020 | Free | Israel | Algology | RCT | 41 | 88 | 8.20 | 17.60 |
| 263 | 272 | Hypnotherapy for Procedural Pain and Distress in Children: A Scoping Review Protocol | Geagea D. | Pain Medicine | 2021 | Free | Australia | Anesthesai practise | Other | 4 | 7 | 1.00 | 1.75 |
| 264 | 270 | Is this back pain killing me? All-cause and cardiovascular specific mortality in older Danish twins with spinal pain | Fernandez M. | European Journal of Pain | 2017 | No | Australia | Algology | Retrospective | 18 | 28 | 2.25 | 3.50 |
| 265 | 270 | Acupuncture and electro-acupuncture for people diagnosed with subacromial pain syndrome: A multicentre randomized trial | Lewis J. | European Journal of Pain | 2017 | No | UK | Algology | RCT | 19 | 35 | 2.38 | 4.38 |
| 266 | 270 | American Society for Enhanced Recovery and Perioperative Quality Initiative Joint Consensus Statement on Nutrition Screening and Therapy Within a Surgical Enhanced Recovery Pathway | Wischmeyer PE. | Anesthesia and analgesia | 2018 | Free | USA | Anesthesia practice | Guideline | 219 | 377 | 31.29 | 53.86 |
| 267 | 269 | In Vitro Apixaban Removal By CytoSorb Whole Blood Adsorber: An Experimental Study | Røed-Undlien H. | Journal of Cardiothoracic and Vascular Anesthesia | 2022 | Free | Canada | Anesthesia practise | Experimental study | 11 | 16 | 3.67 | 5.33 |
| 268 | 268 | The evolution of pediatric sedation and anesthesia patient safety: An interview with Dr Charles J. “Charlie” Coté | Firth PG. | Paediatric Anaesthesia | 2020 | No | USA | Other | Editorial | 0 | 1 | 0.00 | 0.20 |
| 269 | 267 | Extended-age Out-of-sample Validation of Risk Stratification Index 3.0 Models Using Commercial All-payer Claims | Greenwald S. | Anesthesiology | 2022 | Free | USA | Patient safety | Retrospective study | 1 | 1 | 8.00 | 11.33 |
| 270 | 267 | The sound of air: point-of-care lung ultrasound in perioperative medicine | Goffi A. | Canadian Journal of Anesthesia/Journal canadien d'anesthésie | 2018 | Free | Canada | Anesthesia practise | Review | 37 | 65 | 0.14 | 0.14 |
| 271 | 267 | Striatal opioid receptor availability is related to acute and chronic pain perception in arthritis | Brown CA. | Pain (03043959) | 2015 | Free | UK | Algology | Observational study | 24 | 34 | 3.70 | 6.50 |
| 272 | 266 | Management of status epilepticus: a narrative review | Migdady I. | Anaesthesia | 2022 | No | UK | Anesthesia practise | Review | 10 | 16 | 26.33 | 50.67 |
| 273 | 266 | The effect of high altitude commercial air travel on oxygen saturation | Humphreys S. | Anaesthesia | 2005 | Free | Irland | Monitorization | Observational study | 79 | 152 | 0.50 | 0.80 |
| 274 | 265 | Whole Blood Adsorber During CPB and Need for Vasoactive Treatment After Valve Surgery in Acute Endocarditis: A Randomized Controlled Study | Holmén A. | Journal of Cardiothoracic and Vascular Anesthesia | 2022 | No | Sweden | Anesthesai practise | RCT | 15 | 20 | 5.00 | 6.67 |
| 275 | 264 | Dietary intake mediates the relationship of body fat to pain | Emery CF. | Pain (03043959) | 2016 | No | USA | Algology | Observational study | 17 | 26 | 1.89 | 2.89 |
| 276 | 264 | Sociodemographic disparities in chronic pain. based on 12-year longitudinal data | Grol-Prokopczyk H. | Pain (03043959) | 2016 | Free | USA | Algology | Retrospective study | 141 | 241 | 15.67 | 26.78 |
| 277 | 263 | Guidelines on suicide amongst anaesthetists 2019 | Shinde S. | Anaesthesia | 2019 | Free | USA | HCWH | Guideline | 15 | 24 | 2.50 | 4.00 |
| 278 | 262 | Central sensitization: Implications for the diagnosis and treatment of pain | Woolf CJ | Pain (03043959) | 2010 | Free | USA | Algology | Review | 2760 | 5002 | 184.00 | 333.47 |
| 279 | 260 | Medication handling: towards a practical. human-centred approach | Marshall SD. | Anaesthesia | 2018 | Free | Australia | Patient safety | Review | 11 | 22 | 1.57 | 3.14 |
| 280 | 259 | Estimates of Probabilities of Successful Development of Pain Medications: An Analysis of Pharmaceutical Clinical Development Programs from 2000 to 2020 | Maher DP. | Anesthesiology | 2022 | Free | USA | Algology | Retrospective study | 9 | 14 | 22.33 | 32.00 |
| 281 | 259 | Updating the definition of pain | Williams ACC. | Pain (03043959) | 2016 | No | Canada | Anelgology | Guideline | 355 | 1039 | 1.00 | 1.56 |
| 282 | 259 | Fear of going under general anesthesia: A cross-sectional study | Ruhaiyem ME. | Saudi Journal of Anaesthesia | 2016 | Free | Saudi Arabia | Anesthesia practise | Survey | 40 | 117 | 3.56 | 115.44 |
| 283 | 259 | Incidence of iatrogenic opioid dependence or abuse in patients with pain who were exposed to opioid analgesic therapy: a systematic review and meta-analysis | Higgins C. | BJA: The British Journal of Anaesthesia | 2018 | Free | UK | Algology | Review | 79 | 123 | 11.29 | 17.57 |
| 284 | 259 | The efficacy of GlideScope® videolaryngoscopy compared with direct laryngoscopy in children who are difficult to intubate: an analysis from the paediatric difficult intubation registry | Park R. | BJA: The British Journal of Anaesthesia | 2017 | Free | USA | Airway manegement | Observational study | 67 | 96 | 5.00 | 14.63 |
| 285 | 257 | Lidocaine swallow analgesia for severe painful prolonged esophageal disorders | Bamgbade OA. | Saudi Journal of Anaesthesia | 2021 | Free | UK | Algology | Editorial | 1 | 2 | 0.25 | 0.50 |
| 286 | 257 | Perioperative care of obstructive sleep apnea patients: A survey of European anesthesiologists | Bamgbade OA. | Saudi Journal of Anaesthesia | 2021 | Free | Canada | Anesthesia practise | Survey | 2 | 3 | 4.50 | 6.50 |
| 287 | 257 | Cognitive decline in the middle-aged after surgery and anaesthesia: results from the Wisconsin Registry for Alzheimer's Prevention cohort | Bratzke LC. | Anaesthesia | 2018 | Free | USA | Anesthesia practice | Retrospective study | 18 | 26 | 0.29 | 0.43 |
| 288 | 256 | Societal Costs of Prescription Opioid Abuse. Dependence. and Misuse in the United States | Birnbaum HG. | Pain Medicine | 2011 | Free | USA | Algology | Retrospective study | 484 | 864 | 34.57 | 61.71 |
| 289 | 256 | Simple Psychological Interventions for Reducing Pain From Common Needle Procedures in Adults | Boerner KE. | Clinical journal of pain | 2015 | Free | Canada | Algology | Review | N/A | 54 | 3.30 | 5.20 |
| 290 | 256 | Burst Spinal Cord Stimulation: A Clinical Review | Kirketeig T. | Pain Medicine | 2019 | Free | Sweden | Algology | Review | 33 | 52 | 0.00 | 9.00 |
| 291 | 255 | Transnasal Humidified Rapid-Insufflation Ventilatory Exchange (THRIVE): a physiological method of increasing apnoea time in patients with difficult airways | Patel A. | Anaesthesia | 2014 | Free | UK | Airway management | Observational study | 470 | 833 | 8.55 | 18.36 |
| 292 | 255 | Elevation of pain threshold by vaginal stimulation in women | Whipple B. | Pain (03043959) | 1985 | No | USA | Algology | Observational study | 94 | 202 | 11.75 | 20.83 |
| 293 | 254 | International consensus statement on the use of uterotonic agents during caesarean section | Heesen M. | Anaesthesia | 2019 | Free | Switzerland | Anesthesia practice | Guideline | 73 | 137 | 12.17 | 22.83 |
| 294 | 253 | Opioid-free Anesthesia: Time to Regain Our Balance. | Kharasch ED. | Anesthesiology | 2021 | Free | USA | Aneshesia practice | Editorial35 | 35 | 56 | 8.75 | 14.00 |
| 295 | 253 | STR-324. a Stable Analog of Opiorphin. Causes Analgesia in Postoperative Pain by Activating Endogenous Opioid Receptor–dependent Pathways | Sitbon P. | Anesthesiology | 2016 | Free | France | Algology | Experimental study | 17 | 24 | 1.89 | 2.67 |
| 296 | 252 | Back Pain in Outer Space | Penchev R. | Anesthesiology | 2021 | Free | USA | Algology | Review | 8 | 12 | 2.00 | 3.00 |
| 297 | 251 | The myth of the difficult airway: airway management revisited | Huitink JM. | Anaesthesia | 2014 | Free | Netherlands | Airway management | Editorial | 32 | 75 | 2.91 | 6.82 |
| 298 | 250 | Dilemma of Addiction and Respiratory Depression in the Treatment of Pain: A Prototypical Endomorphin as a New Approach. | Webster L. | Pain Medicine | 2019 | Free | USA | Anesthesia practise | Review | 11 | 12 | 2.83 | 7.00 |
| 299 | 250 | Are Invasive Procedures Effective for Chronic Pain? A Systematic Review | Jonas WB. | Pain Medicine | 2018 | Free | USA | Algology | Review | 17 | 42 | 1.57 | 1.71 |
| 300 | 249 | Opioid-induced Hallucinations | Sivanesan E. | Anesthesia and analgesia | 2016 | Free | USA | Anesthesia practise | Review | 25 | 41 | 0.89 | 1.00 |
| 301 | 249 | Exovent: a study of a new negative-pressure ventilatory support device in healthy adults | Members ofthe Exovent DevelopmentGroup | Anaesthesia | 2021 | Free | UK | Intensive Care | Observational study | 8 | 9 | 6.25 | 10.25 |
| 302 | 248 | Personal protective equipment during the coronavirus disease (COVID) 2019 pandemic – a narrative review | Cook TM | Anaesthesia | 2020 | Free | UK | COVID-19 | Review | 406 | 872 | 81.20 | 174.40 |
| 303 | 247 | Complications in obstetric anaesthesia | Maronge L. | Anaesthesia | 2018 | Free | UK | PeriAnesthsia practise | Review | 27 | 72 | 1.71 | 4.14 |
| 304 | 247 | Durable chronic low back pain reductions up to 24 months after treatment for an accessible. 8-week. in-home behavioral skills–based virtual reality program: a randomized controlled trial | Maddox T. | Pain Medicine | 2023 | Free | USA | Algology | RCT | 1 | 6 | 0.50 | 3.00 |
| 305 | 247 | Hallucinations after propofol | Nelson VM. | Anaesthesia | 2007 | Free | UK | Anesthesia practice | Case report | 12 | 29 | .67 | 1.61 |
| 306 | 247 | Movement. posture and low back pain. How do they relate? A replicated single-case design in 12 people with persistent. disabling low back pain | Wernli K. | European Journal of Pain | 2020 | No | Australia | Algology | Observational study | 22 | 43 | 4.40 | 8.60 |
| 307 | 246 | What do patients value learning about pain? A mixed-methods survey on the relevance of target concepts after pain science education | Leake HB. | Pain (03043959) | 2021 | No | Australia | Algology | Survey | 41 | 72 | 10.25 | 18.00 |
| 308 | 245 | A new continuous noninvasive finger cuff device (Vitalstream) for cardiac output that communicates wirelessly via bluetooth or Wi-Fi | Gratz I. | BMC Anesthesiology | 2023 | Free | USA | Monitorizatiom | Observational study | 1 | 2 | 0.50 | 1.00 |
| 309 | 244 | A Randomized Controlled Trial of Vapocoolant for Pediatric Immunization Distress Relief | Cohen LL. | Clinical journal of pain | 2009 | No | USA | Anesthesai practise | RCT | 18 | 42 | 1.13 | 2.63 |
| 310 | 244 | Human factors in preventing complications in anaesthesia: a systematic review | Jones CPL. | Anaesthesia | 2018 | Free | UK | APatient safety | Review | 67 | 125 | 1.29 | 3.00 |
| 311 | 244 | Pain and Suicide: The Other Side of the Opioid Story | Webster LR. | Pain Medicine | 2014 | Free | USA | Algology | Review | 9 | 21 | 6.09 | 11.36 |
| 312 | 243 | Prospective Evaluation of a Multibeat Analysis Cardiac Index Estimation in Patients With Cardiogenic Shock | Kee A. | Journal of Cardiothoracic and Vascular Anesthesia | 2023 | Free | USA | Monitorization | Observational study | 0 | 0 | 4.50 | 7.00 |
| 313 | 243 | Classification-based cognitive functional therapy | Vibe Fersum K. | European Journal of Pain | 2012 | Free | Norway | Algology | RCT | 310 | 566 | 0.00 | 0.00 |
| 314 | 243 | Impaired systemic oxygen extraction long after mild COVID-19: potential perioperative implications | Heerdt PM. | BJA: The British Journal of Anaesthesia | 2021 | Free | USA | COVID-19 | Editorial | 9 | 14 | 77.50 | 141.50 |
| 315 | 243 | Patient–ventilator dyssynchrony in the intensive care unit: A practical approach to diagnosis and management | Oto B. | Anaesthesia and Intensive Care | 2021 | Free | USA | İntensive Care | Review | 5 | 11 | 1.25 | 2.75 |
| 316 | 242 | Survey of chronic pain in Europe: Prevalence. impact on daily life. and treatment | Breivik H. | European Journal of Pain | 2012 | No | Norway | Algology | Survey | 3447 | 7807 | 23.31 | 44.38 |
| 317 | 242 | The Roles of Pain Catastrophizing and Anxiety in the Prediction of Postoperative Pain Intensity | Granot M. | Clinical journal of pain | 2005 | No | Israel | Algology | RCT | 303 | 577 | 172.35 | 390.35 |
| 318 | 241 | Organ transplantation: historical perspective and current practice | Watson CJ. | BJA: The British Journal of Anaesthesia | 2012 | Free | UK | Anesthesia practise | Review | 161 | 414 | 10.69 | 62.77 |
| 319 | 241 | International consensus statement on the peri-operative management of anaemia and iron deficiency | Muñoz M. | Anaesthesia | 2016 | Free | Spain | Anesthesia practice | Guideline | 139 | 816 | 0.44 | 0.89 |
| 320 | 241 | Postoperative delirium. | Whitlock EL. | Minerva Anestesiologica | 2011 | Free | USA | Anesthesia practise | Review | 147 | 303 | 11.50 | 29.57 |
| 321 | 241 | Chronic pain among U.S. sexual minority adults who identify as gay. lesbian. bisexual. or “something else” | Zajacova A. | Pain (03043959) | 2023 | Free | Canada | Algology | Survey | 4 | 8 | 73.50 | 151.50 |
| 322 | 240 | Neuropsychological and Behavioral Outcomes after Exposure of Young Children to Procedures Requiring General Anesthesia | Warner DO. | Anesthesiology | 2018 | Free | USA | Anesthesia practice | Observational study | 306 | 440 | 43.71 | 62.86 |
| 323 | 239 | Practical recommendations for critical care and anesthesiology teams caring for novel coronavirus (2019-nCoV) patients | Wax RS. | Canadian Journal of Anesthesia/Journal canadien d'anesthésie | 2020 | Free | Canada | COVID-19 | Guideline | 572 | 1446 | 18.00 | 25.60 |
| 324 | 239 | An international multidisciplinary consensus statement on the prevention of opioid-related harm in adult surgical patients | Levy N. | Anaesthesia | 2020 | Free | UK | Anesthesia practice | Guideline | 90 | 128 | 114.40 | 289.20 |
| 325 | 238 | Association between “Balance Billing” Legislation and Anesthesia Payments in California: A Retrospective Analysis | Dixit AA. | Anesthesiology | 2023 | Free | USA | HCWS | Review | 1 | 2 | 89.50 | 1.00 |
| 326 | 238 | A systematic review of adverse events in placebo groups of anti-migraine clinical trials | Amanzio M. | Pain (03043959) | 2009 | No | Italy | Algology | Review | 177 | 284 | 11.06 | 17.75 |
| 327 | 236 | Realigning Incentives for Novel Pain Therapeutics | Sinha MS. | Anesthesiology | 2022 | Free | USA | Algology | Editorial | 0 | 0 | 0.00 | 0.00 |
| 328 | 236 | Withholding versus Continuing Angiotensin-converting Enzyme Inhibitors or Angiotensin II Receptor Blockers before Noncardiac Surgery | Roshanov PS. | Anesthesiology | 2017 | Free | UK | Anesthesia practice | Observational study | 182 | 308 | 22.75 | 38.50 |
| 329 | 236 | A national survey of out-of-hours working and fatigue in consultants in anaesthesia and paediatric intensive care in the UK and Ireland | McClelland L. | Anaesthesia | 2019 | Free | UK | HCWS | Retrospective study | 24 | 46 | 4.00 | 7.67 |
| 330 | 235 | In Response. | Guglielminotti J. | Anesthesia and analgesia | 2020 | Free | USA | ARegional anesthesia | Editorial | 0 | 0 | 0.00 | 0.00 |
| 331 | 235 | Pharmacokinetics of intramuscular tranexamic acid in bleeding trauma patients: a clinical trial | Grassin-Delyle S. | BJA: The British Journal of Anaesthesia | 2020 | Free | UK | Trauma | Review | 29 | 51 | 5.80 | 10.20 |
| 332 | 234 | Ultrasound assessment of the inferior vena cava for fluid responsiveness: easy. fun. but unlikely to be helpful | Millington SJ | Canadian Journal of Anesthesia/Journal canadien d'anesthésie | 2019 | Free | Canada | Monitorization | Review | 33 | 71 | 5.50 | 11.83 |
| 333 | 233 | Efficacy of THC/CBD spray in peripheral neuropathic pain | Serpell M. | European Journal of Pain | 2014 | Free | UK | Algology | RCT | 119 | 238 | 10.82 | 21.64 |
| 334 | 233 | Effect of skin tone on the accuracy of the estimation of arterial oxygen saturation by pulse oximetry: a systematic review | Martin D. | BJA: The British Journal of Anaesthesia | 2024 | Free | UK | Monitorisation | Review | N/A | 4 | 0.00 | 4.00 |
| 335 | 232 | The Lancet Series call to action to reduce low value care for low back pain: an update | Buchbinder R. | Pain (03043959) | 2020 | Free | Australia | Algology | Review | 112 | 201 | 1.60 | 2.00 |
| 336 | 232 | Glottic impersonation | Kovacs G. | Canadian Journal of Anesthesia/Journal canadien d'anesthésie | 2016 | Free | Canada | Airway management | Editorial | 8 | 10 | 10.44 | 34.89 |
| 337 | 232 | A prospective. randomised. controlled study examining binaural beat audio and pre-operative anxiety in patients undergoing general anaesthesia for day case surgery* | Padmanabhan R. | Anaesthesia | 2005 | Free | UK | Anesthesi practice | RCT | 94 | 314 | 5.60 | 10.05 |
| 338 | 230 | Substance use disorder in the anaesthetist | Misra U. | Anaesthesia | 2022 | Free | UK | HCWS | Guideline | 2 | 6 | 0.67 | 2.00 |
| 339 | 228 | Anesthesia and Neurodevelopment in Children | Warner DO. | Anesthesiology | 2018 | Free | USA | Anesthesia practice | Editorial | 13 | 29 | 1.86 | 4.14 |
| 340 | 227 | Principles of environmentally sustainable anaesthesia: a global consensus statement from the World Federation of Societies of Anaesthesiologists | White SM. | Anaesthesia | 2021 | Free | UK | Anesthesia practice | Guideline | 63 | 95 | 15.75 | 23.75 |
| 341 | 226 | Cannabis Significantly Reduces the Use of Prescription Opioids and Improves Quality of Life in Authorized Patients: Results of a Large Prospective Study. | Lucas P. | Pain Medicine | 2020 | Free | Canada | Algology | Observational study | 39 | 58 | 7.80 | 11.60 |
| 342 | 226 | Stepwise Ventilator Waveform Assessment to Diagnose Pulmonary Pathophysiology | Flynn BC. | Anesthesiology | 2022 | Free | USA | İntensive Care | Review | 0 | 4 | 0.00 | 1.33 |
| 343 | 225 | A quantitative evaluation of aerosol generation during supraglottic airway insertion and removal | Shrimpton AJ. | Anaesthesia | 2021 | Free | UK | HCWS | Observational study | 6 | 28 | 1.50 | 7.00 |
| 344 | 224 | SARS-CoV-2 infection. COVID-19 and timing of elective surgery | El-Boghdadly K. | Anaesthesia | 2021 | Free | UK | COVID-19 | Observational study | 45 | 149 | 11.25 | 37.25 |
| 345 | 223 | Transorbital endotracheal intubation: a nonstandard approach to a difficult airway | Waldron NH. | Journal of Clinical Anesthesia | 2016 | No | USA | Airway management | Case report | 1 | 7 | 0.11 | 0.78 |
| 346 | 223 | Co-occurrence of chronic pain and anxiety/depression symptoms in U.S. adults: prevalence. functional impacts. and opportunities | De La Rosa JS. | Pain (03043959) | 2023 | Free | USA | Algology | Survey | 5 | 15 | 7.00 | 8.50 |
| 347 | 223 | Implications of nocebo in anaesthesia care | Arrow K. | Anaesthesia | 2022 | No | Australia | Algology | Review | 14 | 17 | 1.67 | 5.00 |
| 348 | 222 | A systematic review and meta-analysis of the prevalence of chronic widespread pain in the general population | Mansfield KE. | Pain (03043959) | 2015 | Free | UK | Algology | Review | 242 | 384 | 7.30 | 14.00 |
| 349 | 222 | No evidence for contraindications to the use of propofol in adults allergic to egg. soy or peanut. | Asserhøj LL. | BJA: The British Journal of Anaesthesia | 2016 | Free | Denmark | Anesthesia practice | Retrospective study | 73 | 140 | 38.00 | 70.00 |
| 350 | 222 | Monitoring depth of anaesthesia in a randomized trial decreases the rate of postoperative delirium but not postoperative cognitive dysfunction | Radtke FM. | BJA: The British Journal of Anaesthesia | 2013 | Free | Germany | Monitorization | RCT | 342 | 630 | 20.17 | 32.25 |
| 351 | 221 | The Unequal Burden of Pain: Confronting Racial and Ethnic Disparities in Pain | Green CR. | Pain Medicine | 2003 | Free | USA | Algology | Review | 852 | 1430 | 11.00 | 21.00 |
| 352 | 221 | Consensus Guidelines on the Use of Intravenous Ketamine Infusions for Acute Pain Management From the American Society of Regional Anesthesia and Pain Medicine. the American Academy of Pain Medicine. and the American Society of Anesthesiologists | Schwenk ES. | Regional Anesthesia & Pain Medicine | 2018 | Free | USA | Algology | Guideline | 242 | 462 | 11.86 | 20.71 |
| 353 | 221 | Clinical guideline and recommendations on pre-operative exercise training in patients awaiting major non-cardiac surgery | Tew GA. | Anaesthesia | 2018 | Free | UK | Anesthesia practice | Guideline | 83 | 145 | 121.71 | 204.29 |
| 354 | 220 | The impact of general anesthesia on child development and school performance: a population-based study | Schneuer FJ. | Paediatric Anaesthesia | 2018 | No | Australia | Anesthesia practice | Retrospective | 64 | 112 | 0.86 | 1.00 |
| 355 | 220 | An experimental randomized study on the analgesic effects of pharmaceutical-grade cannabis in chronic pain patients with fibromyalgia | van de Donk T. | Pain (03043959) | 2018 | Free | Netherlands | Algology | RCT | 140 | 260 | 9.14 | 16.00 |
| 356 | 220 | Handling injectable medications in anaesthesia | Kinsella SM. | Anaesthesia | 2023 | Free | UK | Anesthesia practice | Guideline | 6 | 7 | 70.00 | 130.00 |
| 357 | 219 | Emergency treatment of peri-operative anaphylaxis: Resuscitation Council UK algorithm for anaesthetists | Dodd A. | Anaesthesia | 2024 | Free | UK | Anesthesia practice | Guideline | 2 | 8 | 23.00 | 45.00 |
| 358 | 219 | Brain white matter structural properties predict transition to chronic pain | Mansour AR. | Pain (03043959) | 2013 | Free | USA | Algology | Observational study | 178 | 290 | 0.17 | 0.67 |
| 359 | 219 | Guidelines for safe transfer of the brain-injured patient: trauma and stroke. 2019 | Nathanson MH. | Anaesthesia | 2019 | Free | UK | Trauma | Guideline | 23 | 45 | 29.67 | 48.33 |
| 360 | 218 | What Percentage of Chronic Nonmalignant Pain Patients Exposed to Chronic Opioid Analgesic Therapy Develop Abuse/Addiction and/or Aberrant Drug-Related Behaviors? A Structured Evidence-Based Review | Fishbain DA. | Pain Medicine | 2008 | Free | USA | Algology | Review | 392 | 707 | 23.06 | 41.59 |
| 361 | 218 | Trends and predictors of opioid use after total knee and total hip arthroplasty | Goesling J. | Pain (03043959) | 2016 | Free | USA | Algology | Observational study | 346 | 464 | 0.44 | 0.56 |
| 362 | 218 | Human factors in anaesthesia: a narrative review | Kelly FE. | Anaesthesia | 2023 | No | UK | Patient safety | Review | 19 | 27 | 173.00 | 232.00 |
| 363 | 216 | Measuring Success of Patient Safety Initiatives: The 2023 American Society of Anesthesiologists Practice Guidelines for Monitoring and Antagonism of Neuromuscular Blockade | Brull SJ. | Anesthesiology | 2023 | Free | USA | Patient safety | Editorial | 4 | 5 | 9.50 | 13.50 |
| 364 | 215 | Do Anaesthetists Need to Wear Surgical Masks in the Operating Theatre? A Literature Review with Evidence-Based Recommendations | Skinner MW. | Anaesthesia and Intensive Care | 2019 | Free | Tasmania | Patient safety | Review | 18 | 30 | 3.00 | 5.00 |
| 365 | 215 | Incident and long-term opioid therapy among patients with psychiatric conditions and medications | Sellers D. | Pain (03043959) | 2017 | Free | USA | Algology | Retrospective study | 87 | 124 | 10.88 | 15.50 |
| 366 | 215 | Respiratory complications of anaesthesia | Mills GH. | Anaesthesia | 2018 | Free | UK | AAnesthesia practise | Review | 35 | 76 | 5.00 | 10.86 |
| 367 | 214 | Cardiovascular complications after non-cardiac surgery | Sellers D. | Anaesthesia | 2018 | Free | Canada | Anesthesia practise | Review | 53 | 110 | 7.57 | 15.71 |
| 368 | 213 | ECMO in COVID-19 Patients: A Systematic Review and Meta-analysis | Bertini P. | Journal of Cardiothoracic and Vascular Anesthesia | 2021 | Free | Italy | COVID-19 | Review | 38 | 92 | 9.50 | 23.00 |
| 369 | 213 | Neuroinflammation and Central Sensitization in Chronic and Widespread Pain | Ji RR. | Anesthesiology | 2018 | Free | USA | Algology | Review | 674 | 1048 | 96.29 | 149.71 |
| 370 | 212 | Spinal cord injury arising in anaesthesia practice | Hewson DW. | Anaesthesia | 2018 | Free | UK | Anesthesia practise | Review | 42 | 80 | 6.00 | 11.43 |
| 371 | 212 | Pain sensitivity is inversely related to regional grey matter density in the brain | Emerson NM. | Pain (03043959) | 2013 | Free | Israel | Algology | Review | 83 | 133 | 1.75 | 2.33 |
| 372 | 212 | Human induced pluripotent stem cell-derived GABAergic interneuron transplants attenuate neuropathic pain. | Manion J. | Pain (03043959) | 2020 | No | Australia | Algology | Experimental study | 21 | 28 | 16.60 | 26.60 |
| 373 | 212 | Taming the Ketamine Tiger | Domino EF. | Anesthesiology | 2010 | Free | USA | AlAnesthesia practise | Review | 337 | 602 | 3.07 | 9.07 |
| 374 | 212 | A Systematic Review and Meta-analysis of Yoga for Low Back Pain | Cramer H. | Clinical journal of pain | 2013 | No | Germany | Algology | Review | 221 | 555 | 18.42 | 46.25 |
| 375 | 211 | Butterbur root extract and music therapy in the prevention of childhood migraine: An explorative study | Oelkers-Ax R. | European Journal of Pain | 2012 | No | Germany | Anesthesai practise | RCT | 46 | 136 | 25.92 | 46.31 |
| 376 | 211 | The mental health of staff working on intensive care units over the COVID-19 winter surge of 2020 in England: a cross sectional survey | Hall CE. | BJA: The British Journal of Anaesthesia | 2022 | Free | UK | COVID-19 | Observational study | 17 | 37 | 4.67 | 10.67 |
| 377 | 211 | Renal impairment and its impact on clinical outcomes in patients who are critically ill with COVID-19: a multicentre observational study | Gasparini M. | Anaesthesia | 2020 | Free | UK | COVID-19 | Observational study | 25 | 41 | 2.60 | 23.00 |
| 378 | 211 | Risk to health from COVID?19 for anaesthetists and intensivists – a narrative review | Cook TM. | Anaesthesia | 2020 | Free | UK | COVID-19 | Review | 26 | 56 | 5.00 | 8.20 |
| 379 | 211 | Incidence of Connected Consciousness after Tracheal Intubation | Sanders RD. | Anesthesiology | 2017 | Free | USA | Airway management | Observational study | 13 | 115 | 3.25 | 7.00 |
| 380 | 210 | Tribute to Dr. Takuo Aoyagi. inventor of pulse oximetry | Miyasaka K. | Journal of Anesthesia | 2021 | Free | Japan | Other | Editorial | 14 | 32 | 4.25 | 9.25 |
| 381 | 210 | The Association Between Headaches and Temporomandibular Disorders is Confounded by Bruxism and Somatic Symptoms | van der Meer HA. | Clinical journal of pain | 2017 | No | Netherlands | Algology | Retrospective | 29 | 65 | 3.63 | 8.13 |
| 382 | 210 | Evidence-based treatment recommendations for neck and low back pain across Europe: A systematic review of guidelines | Corp N. | European Journal of Pain | 2020 | Free | UK | Algology | Guideline | 154 | 182 | 30.80 | 36.40 |
| 383 | 209 | Continuous hemoglobin measurement during frontal advancement operations can improve patient outcomes | Saracoglu A | Journal of Clinical Monitoring and Computing | 2022 | No | Turkey | Monitorisation | Retrospective | 1 | 11 | 6.33 | 12.67 |
| 384 | 209 | Incidence of accidental awareness during general anaesthesia in obstetrics: a multicentre. prospective cohort study | Odor PM. | Anaesthesia | 2021 | Free | UK | Anesthesia practice | Observational study | 19 | 38 | 0.25 | 2.75 |
| 385 | 208 | Pain as a reward: Changing the meaning of pain from negative to positive co-activates opioid and cannabinoid systems | Benedetti F. | Pain (03043959) | 2012 | No | Italy | Algology | RCT | 77 | 137 | 5.92 | 10.54 |
| 386 | 206 | Processed Electroencephalogram Monitoring and Postoperative Delirium | MacKenzie KK. | Anesthesiology | 2018 | Free | USA | Anesthesia practise | Review | 67 | 123 | 1.57 | 2.57 |
| 387 | 206 | Pain in Malignant Pleural Mesothelioma: A Prospective Characterization Study. | MacLeod N. | Pain Medicine | 2016 | Free | UK | Algology | Observational study | 11 | 18 | 7.67 | 18.00 |
| 388 | 206 | The Use of Ginger (Zingiber officinale) for the Treatment of Pain: A Systematic Review of Clinical Trials | Terry R. | Pain Medicine | 2011 | Free | UK | Algology | Review | 69 | 162 | 4.79 | 8.79 |
| 389 | 205 | Oxycodone Ingestion Patterns in Acute Fracture Pain With Digital Pills | Chai PR. | Anesthesia and analgesia | 2017 | Free | USA | Algology | Observational study | 30 | 41 | 3.75 | 5.13 |
| 390 | 205 | Chronic pain: a review of its epidemiology and associated factors in population-based studies | Mills SEE. | BJA: The British Journal of Anaesthesia | 2019 | Free | UK | Algology | Review | 677 | 1415 | 112.83 | 235.83 |
| 391 | 204 | Sexual hallucinations during and after sedation and anaesthesia | Balasubramaniam B. | Anaesthesia | 2003 | Free | UK | Anesthsia practise | Review | 35 | 67 | 1.59 | 3.05 |
| 392 | 203 | The measurement of adult blood pressure and management of hypertension before elective surgery | Hartle A. | Anaesthesia | 2016 | Free | UK | Anesthesia practice | Guideline | 91 | 155 | 10.11 | 17.22 |
| 393 | 203 | Surgical activity in England and Wales during the COVID-19 pandemic: a nationwide observational cohort study | Dobbs TD. | BJA: The British Journal of Anaesthesia | 2021 | Free | UK | COVID -19 | Retrospective study | 57 | 90 | 14.25 | 22.50 |
| 394 | 202 | Are weather conditions associated with chronic musculoskeletal pain- Review of results and methodologies. | Beukenhorst AL. | Pain (03043959) | 2019 | Free | UK | Algology | Review | 22 | 31 | 0.67 | 1.00 |
| 395 | 202 | Inches. Centimeters. and Yards | Dasgupta N. | Clinical journal of pain | 2021 | Free | USA | Algology | Review | 13 | 19 | 5.50 | 7.75 |
| 396 | 202 | The association between age at menarche and chronic pain outcomes in women: the Tromsø Study. 2007 to 2016 | Lund CI. | Pain (03043959) | 2022 | Free | Norway | Algoloy | Retrospective study | 4 | 6 | 4.33 | 6.33 |
| 397 | 201 | Frequency of surgical treatment and related hospital procedures in the UK: a national ecological study using hospital episode statistics | Abbott TEF. | BJA: The British Journal of Anaesthesia | 2017 | Free | UK | HCWS | Retrospective study | 136 | 225 | 17.00 | 28.13 |
| 398 | 201 | Videolaryngoscopy vs. fibreoptic bronchoscopy for awake tracheal intubation: a systematic review and meta-analysis | Alhomary M. | Anaesthesia | 2018 | Free | New Zealand | Airway management | Review | 84 | 184 | 12.00 | 26.29 |
| 399 | 200 | Influence of age and gender on autonomic regulation of heart | Abhishekh HA. | Journal of Clinical Monitoring and Computing | 2013 | No | India | Other | Observational study | 137 | 285 | 11.42 | 23.75 |
| 400 | 200 | Pain Management: A Fundamental Human Right | Brennan F. | Anesthesia and analgesia | 2007 | Free | Australia | Algology | Review | 614 | 1535 | 3.33 | 5.67 |
| 401 | 200 | Renal complications of anaesthesia | McKinlay J. | Anaesthesia | 2018 | Free | UK | Anesthesia practice | Review | 24 | 43 | 87.71 | 219.29 |
| 402 | 200 | Pain Psychology: A Global Needs Assessment and National Call to Action | Darnall BD. | Pain Medicine | 2016 | Free | USA | Algology | Observational study | 60 | 102 | 2.67 | 4.78 |
| 403 | 198 | Sham Surgery in Orthopedics: A Systematic Review of the Literature | Louw A. | Pain Medicine | 2016 | Free | USA | Algology | Review | 35 | 67 | 24.33 | 60.56 |
| 404 | 198 | Perioperative Pulmonary Atelectasis: Part I. Biology and Mechanisms | Zeng C. | Anesthesiology | 2021 | Free | USA | Anesthesia practise | Review | 41 | 62 | 8.75 | 16.75 |
| 405 | 198 | Outbreak of a new coronavirus: what anaesthetists should know | Peng PWH. | BJA: The British Journal of Anaesthesia | 2020 | Free | Canada | COVID-19 | Editorial | 219 | 545 | 8.20 | 12.40 |
| 406 | 197 | The prevalence and years lived with disability caused by low back pain in China. 1990 to 2016 | Wu A. | Pain (03043959) | 2018 | Free | China | Algology | Review | 58 | 90 | 8.29 | 12.86 |
| 407 | 197 | Evidence for compromised data integrity in studies of liberal peri-operative inspired oxygen | Myles PS. | Anaesthesia | 2019 | Free | Australia | Monitorisation | Review | 40 | 55 | 6.67 | 9.17 |
| 408 | 196 | The impact of COVID-19 on anaesthesia and critical care services in the UK: a serial service evaluation* | Kursumovic E. | Anaesthesia | 2021 | Free | UK | COVID-19 | Retrospective study | 25 | 40 | 24.75 | 40.50 |
| 409 | 196 | Strategies for the prevention of airway complications – a narrative review | Cook T. M. | Anaesthesia | 2017 | Free | UK | Airway management | Review | 99 | 162 | 3.13 | 5.00 |
| 410 | 195 | Risk of Major Complications After Perioperative Norepinephrine Infusion Through Peripheral Intravenous Lines in a Multicenter Study. | Pancaro C. | Anesthesia and analgesia | 2019 | Free | USA | Anesthsia practice | Retrospective study | 47 | 79 | 7.83 | 13.17 |
| 411 | 195 | Towards a neurophysiological signature for fibromyalgia | López-Solà M. | Pain (03043959) | 2016 | Free | USA | Algology | Observational study | 162 | 280 | 18.00 | 31.11 |
| 412 | 194 | Noninvasive neuromodulation of subregions of the human insula differentially affect pain processing and heart-rate variability: a within-subjects pseudo-randomized trial. | Legon W. | Pain (03043959) | 2024 | Free | USA | Algology | RCT | 0 | 8 | 0.00 | 8.00 |
| 413 | 194 | Automated preclinical detection of mechanical pain hypersensitivity and analgesia | Zhang Z. | Pain (03043959) | 2022 | Free | USA | Algology | Experimental study | 11 | 22 | 3.67 | 7.33 |
| 414 | 193 | Pain or nociception? Subjective experience mediates the effects of acute noxious heat on autonomic responses | Mischkowski D. | Pain (03043959) | 2017 | Free | USA | Algology | Observational study | 5 | 80 | 0.00 | 0.00 |
| 415 | 193 | Ipsilateral Intravenous Catheter Placement in Breast Cancer Surgery Patients | Naranjo J. | Anesthesia and analgesia | 2021 | Free | USA | Algology | Retrospective study | 12 | 7 | 3.00 | 1.75 |
| 416 | 193 | Images from the Frontlines of the COVID-19 Pandemic |  | Anesthesiology | 2020 | Free | USA | COVID-19 | Editorial | 0 | 0 | 1.00 | 16.00 |
| 417 | 192 | Sodium Bicarbonate in Different Critically Ill Conditions: From Physiology to Clinical Practice | Coppola S. | Anesthesiology | 2021 | Free | Italy | Others | Review | N/A | 19 | 326.25 | 562.75 |
| 418 | 192 | A model to predict difficult airway alerts after videolaryngoscopy in adults with anticipated difficult airways – the VIDIAC score | Kohse EK. | Anaesthesia | 2022 | Free | Germany | Airway management | Observational study | 13 | 18 | 21.67 | 37.67 |
| 419 | 192 | Early oral protein-containing diets following elective lower gastrointestinal tract surgery in adults: a meta-analysis of randomized clinical trials | Pu H. | Perioperative Medicine | 2021 | Free | Australia | Anesthesia practice | Review | 5 | 12 | 3.25 | 4.50 |
| 420 | 192 | Percutaneous peripheral nerve stimulation for the treatment of chronic neuropathic postamputation pain: a multicenter. randomized. placebo-controlled trial. | Gilmore C. | Regional Anesthesia & Pain Medicine | 2019 | Free | USA | Algology | Observational study | 65 | 113 | 0.00 | 3.17 |
| 421 | 192 | Difficult Airway Society 2015 guidelines for management of unanticipated difficult intubation in adults . | Frerk C. | BJA: The British Journal of Anaesthesia | 2015 | Free | UK | Airway managemet | Guideline | 1305 | 2251 | 0.50 | 1.20 |
| 422 | 191 | Cryoneurolysis and Peripheral Nerve Stimulation: Reply | Ilfeld BM. | Anesthesiology | 2021 | Free | USA | Algology | Editorial | 1 | 1 | 0.25 | 0.25 |
| 423 | 191 | Efficacy of Ginger for Alleviating the Symptoms of Primary Dysmenorrhea: A Systematic Review and Meta-analysis of Randomized Clinical Trials | Daily JW. | Pain Medicine | 2015 | Free | USA | Algology | Review | 46 | 139 | 3.00 | 5.90 |
| 424 | 191 | Prevention and management of intra-operative pain during caesarean section under neuraxial anaesthesia: a technical and interpersonal approach | Plaat F. | Anaesthesia | 2022 | Free | UK | Anesthesia practise | Guideline | 30 | 59 | 41.33 | 62.33 |
| 425 | 191 | Dos terapias conocidas podrían ser efectivas como adyuvantes en el paciente crítico infectado por COVID-19 | Hernández A. . | Revista española de anestesiología y reanimación | 2020 | Free | Espana | PrCOVID-19 | Review | 50 | 104 | 10.00 | 10.00 |
| 426 | 191 | Anaesthetic depth and delirium after major surgery: a randomised clinical trial | Evered LA. | BJA: The British Journal of Anaesthesia | 2021 | Free | Australia | Anesthesia practice | RCT | 124 | 187 | 11.50 | 34.75 |
| 427 | 190 | Primary Care Physicians’ Knowledge And Attitudes Regarding Prescription Opioid Abuse and Diversion | Hwang CS. | Clinical journal of pain | 2016 | No | USA | Anesthesia practise | Survey | 30 | 49 | 0.22 | 0.56 |
| 428 | 190 | The Myth of Baby “Anaesthesia” | Defalque RJ. | Anesthesiology | 2009 | Free | UK | Other | Editorial | 2 | 5 | 1.88 | 3.06 |
| 429 | 189 | One night of total sleep deprivation promotes a state of generalized hyperalgesia: A surrogate pain model to study the relationship of insomnia and pain | Schuh-Hofer S. | Pain (03043959) | 2013 | No | Germany | Algology | Observational study | 206 | 322 | 17.17 | 26.83 |
| 430 | 187 | Neuroimaging of Pain | Martucci KT. | Anesthesiology | 2018 | Free | USA | Algology | Review | 95 | 155 | 6.71 | 9.57 |
| 431 | 187 | Cuffed vs. uncuffed tracheal tubes in children: a randomised controlled trial comparing leak. tidal volume and complications | Chambers NA. | Anaesthesia | 2017 | Free | Australia | Anesthesia practice | RCT | 47 | 67 | 11.88 | 19.38 |
| 432 | 185 | Educational disparities in joint pain within and across US states: do macro sociopolitical contexts matter? | Huang R. | Pain (03043959) | 2023 | Free | USA | Algology | Survey | 0 | 3 | 9.00 | 38.00 |
| 433 | 185 | Consensus practice guidelines on interventions for cervical spine (facet) joint pain from a multispecialty international working group | Hurley RW. | Regional Anesthesia & Pain Medicine | 2021 | Free | USA | Algology | Guideline | 18 | 76 | 17.50 | 26.25 |
| 434 | 185 | Oxygen Reserve Index | Szmuk P. | Anesthesiology | 2016 | Free | Netherlands | Patient safty | Editorial | 70 | 105 | 0.00 | 0.33 |
| 435 | 184 | How systems engineering can improve care in the ICU | Lecamwasam H. | Journal of Clinical Anesthesia | 2020 | Free | USA | Intensive care | Editorial | 0 | 1 | 0.00 | 0.20 |
| 436 | 184 | Physical activity behavior predicts endogenous pain modulation in older adults | Naugle KM. | Pain (03043959) | 2017 | No | USA | Algology | Observational study | 93 | 146 | 11.63 | 18.25 |
| 437 | 184 | Surgery for chronic musculoskeletal pain: the question of evidence. | Harris IA. | Pain (03043959) | 2020 | No | Australia | Algology | Review | 10 | 23 | 2.00 | 4.60 |
| 438 | 183 | Management of traumatic brain injury: a narrative review of current evidence | Wiles MD | Anaesthesia | 2022 | No | UK | Trauma | Review | 26 | 43 | 50.33 | 87.67 |
| 439 | 183 | Effectiveness of small daily amounts of progressive resistance training for frequent neck/shoulder pain: Randomised controlled trial | Andersen LL. | Pain (03043959) | 2010 | No | Denmark | Algology | Review | 151 | 263 | 1.73 | 2.87 |
| 440 | 183 | Patient characteristics. anaesthetic workload and techniques in the UK: an analysis from the 7th National Audit Project (NAP7) activity survey | Kane AD. | Anaesthesia | 2023 | Free | UK | Anesthesia practise | Survey | 36 | 54 | 30.50 | 47.50 |
| 441 | 183 | Communicating with children about ‘everyday’ pain and injury: A Delphi study | Wallwork SB. | European Journal of Pain | 2022 | Free | AUstralia | Anesthesia practise | Survey | 6 | 11 | 37.67 | 58.67 |
| 442 | 183 | Guideline for the management of hip fractures 2020 | Griffiths R. | Anaesthesia | 2020 | Free | UK | Anesthesia practise | Guideline | 113 | 176 | 1.20 | 2.20 |
| 443 | 183 | An international multidisciplinary consensus statement on fasting before procedural sedation in adults and children | Green SM. | Anaesthesia | 2019 | Free | USA | Anesthesia practice | Guideline | 61 | 95 | 6.00 | 9.00 |
| 444 | 182 | Unusual difficult airway due to the presence of a large facial foreign body | Dalela S. | Canadian Journal of Anesthesia/Journal canadien d'anesthésie | 2014 | Free | USA | Airway management | Case report | 0 | 1 | 0.00 | 0.09 |
| 445 | 182 | Preliminary validation of the Michigan Body Map | Brummett CM. | Pain (03043959) | 2016 | Free | USA | Algology | Observational study | 101 | 125 | 2.67 | 5.67 |
| 446 | 182 | Guideline on anaesthesia and sedation in breastfeeding women 2020 | Mitchell J. | Anaesthesia | 2020 | Free | UK | Anesthesia practice | Guideline | 24 | 51 | 0.00 | 25.00 |
| 447 | 181 | Fourth Consensus Guidelines for the Management of Postoperative Nausea and Vomiting. | Gan TJ. | Anesthesia and analgesia | 2020 | Free | USA | Anesthesia practise | Guideline | 453 | 808 | 8.40 | 17.20 |
| 448 | 181 | Airway management in patients with suspected or confirmed traumatic spinal cord injury: a narrative review of current evidence | Whiles MD | Anaesthesia | 2022 | Free | UK | Airway Management | Review | 7 | 16 | 0.00 | 269.33 |
| 449 | 181 | Oleum menthae piperitae (Pfefferminzöl) in der Akuttherapie des Kopfschmerzes vom Spannungstyp | ZGöbel H. | Der Schmerz | 2016 | No | Germany | Algology | Review | 9 | 10 | 1.00 | 1.11 |
| 450 | 181 | Psychological factors not strength deficits are associated with severity of gluteal tendinopathy: A cross-sectional study | Plinsinga ML. | European Journal of Pain | 2018 | No | Australia | Algology | Observational study | 31 | 46 | 1.00 | 2.29 |
| 451 | 181 | Malignant hyperthermia 2020 | Hopkins PM. | Anaesthesia | 2021 | Free | UK | Anesthesia practice | Guideline | 42 | 86 | 7.75 | 11.50 |
| 452 | 180 | Chronic Pain is Associated with a Brain Aging Biomarker in Community-Dwelling Older Adults | Cruz-Almeida Y. | Pain (03043959) | 2019 | Free | USA | Algology | Survey | 60 | 75 | 3.17 | 6.00 |
| 453 | 180 | A Decision Tree Approach to Airway Management Pathways in the 2022 Difficult Airway Algorithm of the American Society of Anesthesiologists | Rosenblatt WH. | Anesthesia and analgesia | 2022 | Free | USA | Airway management | Review | 19 | 36 | 20.00 | 25.00 |
| 454 | 180 | Nutrition Status Optimization for Improved Perioperative Outcomes | Williams. D.G.A. | Current Anesthesiology Reports | 2022 | Free | USA | Anesthesia pracice | Review | 2 | 6 | 0.67 | 2.00 |
| 455 | 179 | Simulation Study of Rested Versus Sleep-deprived Anesthesiologists | Howard SK. | Anesthesiology | 2003 | Free | USA | HCWS | Other | 120 | 279 | 5.45 | 12.68 |
| 456 | 178 | Effectiveness of Traditional Chinese “Gua Sha” Therapy in Patients with Chronic Neck Pain: A Randomized Controlled Trial | Braun M. | Pain Medicine | 2011 | Free | Germany | Algology | RCT | 44 | 131 | 3.14 | 9.36 |
| 457 | 178 | Race differences in pain and pain-related risk factors among former professional American-style football players | Edwards RR. | Pain (03043959) | 2023 | Free | USA | Algology | Survey | 0 | 1 | 183.00 | 333.00 |
| 458 | 178 | Venous Function and Central Venous Pressure | Gelman S | Anesthesiology | 2008 | Free | USA | Others | Review | 366 | 666 | 0.00 | 0.06 |
| 459 | 177 | Clinical tests for confirming tracheal intubation or excluding oesophageal intubation: a diagnostic test accuracy systematic review and meta?analysis | Hansel J. | Anaesthesia | 2023 | Free | UK | Airway management | Review | 13 | 18 | 0.00 | 0.00 |
| 460 | 177 | A Letter to Medical Students from an Anesthesiologist | Ok S.M (Michale Sung-jin Ok) | Anesthesiology | 2021 | Free | USA | Other | Editorial | 0 | 0 | 6.75 | 12.75 |
| 461 | 177 | Pharmacological interventions for chronic pain in children: an overview of systematic reviews | Eccleston C. | Pain (03043959) | 2019 | No | UK | Algology | Review | 56 | 88 | 9.33 | 14.67 |
| 462 | 177 | Factors influencing the selection of hydrocodone and oxycodone as primary opioids in substance abusers seeking treatment in the United States | Cicero TJ. | Pain (03043959) | 2013 | No | USA | Algology | Review | 33 | 61 | 2.83 | 4.42 |
| 463 | 177 | Appropriate Clinical Use of Lactate Measurements. | Pino RM. | Anesthesiology | 2020 | Free | USA | POthers | Review | 27 | 51 | 2.60 | 3.60 |
| 464 | 177 | Perioperative Point-of-Care Ultrasound: From Concept to Application. | Ramsingh D. | Anesthesiology | 2020 | Free | USA | Monitorization | Review | 34 | 53 | 6.60 | 12.20 |
| 465 | 176 | The contemporary pulmonary artery catheter. Part 1: placement and waveform analysis | Bootsma IT. | Journal of Clinical Monitoring and Computing | 2021 | Free | Netherlands | Monitorization | Review | 18 | 36 | 0.25 | 0.25 |
| 466 | 176 | Needle tip tracking for ultrasound-guided peripheral nerve block procedures—An observer blinded. randomised. controlled. crossover study on a phantom model | Kåsine T. | Acta Anaesthesiologica Scandinavica | 2019 | Free | Norway | Regional anesthesia | RCT | 17 | 22 | 2.83 | 3.67 |
| 467 | 176 | Human factors and the safety of surgical and anaesthetic care | Marshall SD. | Anaesthesia | 2020 | Free | Australia | Patient safety | Review | 14 | 32 | 5.80 | 16.80 |
| 468 | 176 | Intracranial Nasogastric Tube Placement in a Nontrauma Patient | Patel GP. | Anesthesiology | 2023 | No | Georgia | Anesthesia practice | Case report | 1 | 1 | 7.00 | 16.00 |
| 469 | 176 | Ingestion of a THC-Rich Cannabis Oil in People with Fibromyalgia: A Randomized. Double-Blind. Placebo-Controlled Clinical Trial | Chaves C. | Pain Medicine | 2020 | Free | Brasil | Algology | RCT | 29 | 84 | 3.60 | 7.20 |
| 470 | 175 | Tranexamic acid for safer surgery: the time is now | UK Royal Colleges Tranexamic Acid in Surgery Implementation Group. | BJA: The British Journal of Anaesthesia | 2022 | Free | UK | Anesthesia practise | Review | 5 | 20 | 1.67 | 6.67 |
| 471 | 175 | An observational feasibility study of a new anaesthesia drug storage tray | Almghairbi DS. | Anaesthesia | 2018 | Free | UK | Anesthesia practice | Observational study | 12 | 23 | 1.71 | 3.29 |
| 472 | 174 | Efficacy of multidisciplinary pain treatment centers: a meta-analytic review | Flor H. | Pain (03043959) | 1992 | No | USA | Algology | Review | 806 | 1831 | 7.61 | 14.82 |
| 473 | 174 | Clinical course of non-specific low back pain | Itz CJ. | European Journal of Pain | 2012 | No | Netherlands | Algology | Review | 251 | 489 | 62.00 | 140.85 |
| 474 | 173 | Right ventricular dysfunction in patients with COVID-19 pneumonitis whose lungs are mechanically ventilated: a multicentre prospective cohort study | McCall PJ. | Anaesthesia | 2022 | Free | UK | COVID-19 | Observational study | 11 | 15 | 3.67 | 5.00 |
| 475 | 173 | Neurological complications of surgery and anaesthesia | Mashour GA. | BJA: The British Journal of Anaesthesia | 2014 | Free | USA | AirAnesthesia practise | Review | 97 | 179 | 8.82 | 16.27 |
| 476 | 172 | Advanced Monitoring Is Associated with Fewer Alarm Events During Planned Moderate Procedure-Related Sedation | Applegate RL 2nd. | Anesthesia and analgesia | 2016 | Free | USA | Regional | RCT | 11 | 21 | 12.11 | 37.33 |
| 477 | 172 | Pre-optimisation of patients undergoing emergency laparotomy: a review of best practice | Poulton T. | Anaesthesia | 2019 | Free | England | Anesthesia practise | Review | 23 | 45 | 1.83 | 3.50 |
| 478 | 172 | Quantification of changes in myofascial trigger point sensitivity with the pressure algometer following passive stretch | Jaeger B. | Pain (03043959) | 1986 | No | USA | Algology | Observational study | 106 | 336 | 0.59 | 1.15 |
| 479 | 171 | Low back pain and the social determinants of health: a systematic review and narrative synthesis. | Karran EL. | Pain (03043959) | 2020 | No | Australia | Algology | Review | 93 | 161 | 2.60 | 3.40 |
| 480 | 171 | Clinical significance of angiotensin-converting enzyme 2 receptors for severe acute respiratory syndrome coronavirus 2 (COVID-19) on peripheral small-fiber sensory neurons is unknown today. | Oaklander AL. | Pain (03043959) | 2020 | Free | USA | COVID-19 | Editorial | 13 | 17 | 18.60 | 32.20 |
| 481 | 170 | Sevoflurane requirements during electroencephalogram (EEG)-guided vs standard anesthesia Care in Children: A randomized controlled trial | Long MHY. | Journal of Clinical Anesthesia | 2022 | No | Republic of Singapore | Anesthesai practise | RCT | 8 | 13 | 8.33 | 11.67 |
| 482 | 170 | Effects of open-label placebo on pain. functional disability. and spine mobility in patients with chronic back pain: a randomized controlled trial. | Ramalingam G. | Pain (03043959) | 2019 | No | Germany | Algology | RCT | 67 | 115 | 13.17 | 21.50 |
| 483 | 170 | Pain as a risk factor for common mental disorders. Results from the Netherlands Mental Health Survey and Incidence Study-2 | de Heer EW. | Pain (03043959) | 2017 | No | Netherlands | Algology | Observational study | 49 | 82 | 6.13 | 10.25 |
| 484 | 170 | Persistent pain after motor vehicle collision | Beaudoin FL. | Pain (03043959) | 2016 | Free | USA | Algology | Observational study | 25 | 35 | 7.44 | 12.78 |
| 485 | 170 | Sleep problems and pain | Bonvanie IJ. | Pain (03043959) | 2016 | No | Netherlands | Algology | Observational study | 79 | 129 | 0.00 | 1.56 |
| 486 | 169 | Health care providers' judgments in chronic pain | Schäfer G. | Pain (03043959) | 2016 | No | Canada | Algology | Survey | 91 | 156 | 10.11 | 17.33 |
| 487 | 168 | Prediction of fluid responsiveness in mechanically ventilated patients in surgical intensive care unit by pleth variability index and inferior vena cava diameter | Aboelnile DBMK. | Ain-Shams Journal of Anesthesiology | 2020 | Free | EGYPT | Intevsive care | Observational study | 0 | 8 | 8.20 | 10.00 |
| 488 | 168 | Complications related to peri-operative transoesophageal echocardiography – a one-year prospective national audit by the Association of Cardiothoracic Anaesthesia and Critical Care | Ramalingam G. | Anaesthesia | 2019 | Free | UK. Hong Kong | Anesthesia practice | Observational study | 41 | 50 | 0.00 | 1.33 |
| 489 | 167 | Reduced postoperative pain using Nociception Level-guided fentanyl dosing during sevoflurane anaesthesia: a randomised controlled trial | Meijer F. | BJA: The British Journal of Anaesthesia | 2020 | Free | Netherlands | Algology | RCT | 57 | 93 | 3.00 | 4.80 |
| 490 | 167 | Advancing women in academic medicine: ten strategies to use every day | Geagea A. | Canadian Journal of Anesthesia/Journal canadien d'anesthésie | 2019 | Free | Canada | Other | Editorial | 15 | 24 | 9.50 | 15.50 |
| 491 | 166 | 'Green-gional' anesthesia: the non-polluting benefits of regional anesthesia to decrease greenhouse gases and attenuate climate change | Kuvadia M. | Regional Anesthesia & Pain Medicine | 2020 | No | USA | Anesthesai practise | Other | 25 | 34 | 0.20 | 1.00 |
| 492 | 166 | Symptom perception. placebo effects. and the Bayesian brain | Ongaro G. | Pain (03043959) | 2018 | Free | USA | Others | Review | 115 | 85 | 3.57 | 4.86 |
| 493 | 166 | Future directions in regional anaesthesia: not just for the cognoscenti | Selak T. | Anaesthesia | 2019 | Free | Australia | Regional anesthesia | Editorial | 1 | 5 | 2.17 | 3.50 |
| 494 | 166 | A genetic polymorphism that is associated with mitochondrial energy metabolism increases risk of fibromyalgia. | van Tilburg MAL. | Pain (03043959) | 2020 | Free | USA | Algology | Observational study | 13 | 21 | 0.60 | 8.80 |
| 495 | 166 | Tanezumab for chronic low back pain: a randomized. double-blind. placebo- and active-controlled. phase 3 study of efficacy and safety | Markman JD. | Pain (03043959) | 2020 | Free | USA | Algology | RCT | 3 | 44 | 23.00 | 37.00 |
| 496 | 165 | “Listen to me. learn from me”: a priority setting partnership for shaping interdisciplinary pain training to strengthen chronic pain care | Slater H. | Pain (03043959) | 2022 | Free | Australia | Algology | Survey | 12 | 24 | 4.00 | 8.00 |
| 497 | 165 | Practice Advisory for Preanesthesia Evaluation | Committee on Standards and Practice Parameters. | Anesthesiology | 2012 | Free | USA | Anesthesia practice | Guideline | 203 | 464 | 15.62 | 35.69 |
| 498 | 164 | ATLS: Archaic Trauma Life Support? | Wiles MD. | Anaesthesia | 2015 | Free | UK | Trauma | Editorial | 13 | 27 | 1.30 | 2.70 |
| 499 | 164 | Chronic pain after COVID-19: implications for rehabilitation | Kemp HI. | BJA: The British Journal of Anaesthesia | 2020 | Free | UK | Algology | Editorial | 93 | 212 | 18.60 | 42.40 |
| 500 | 164 | The Role of Opioid Prescription in Incident Opioid Abuse and Dependence Among Individuals With Chronic Noncancer Pain | Edlund MJ. | Clinical journal of pain | 2014 | Free | USA | Algology | Retrospective | 344 | 576 | 31.27 | 52.36 |
| AAS: Altmetric Atention Score. OA: Open Access. RCT: Randomised Control Trial. HCWS: Healtcare Worker Safety, **WoS: Web of Science** | | | | | | | | | | | |  |  |

| Appendix 3: Detailed Analysis of Altmetric Attention Scores (AAS) for the Top 500 Publications | | | | | | | | | | | | | | | | | | | | |
| --- | --- | --- | --- | --- | --- | --- | --- | --- | --- | --- | --- | --- | --- | --- | --- | --- | --- | --- | --- | --- |
| **Rank** | **AAS** | **News mentions** | **Blog mentions** | **Policy mentions** | **Patent mentions** | **X mentions** | **Peer review mentions** | **Weibo mentions** | **Facebook mentions** | **Wikipedia mentions** | **Google+ mentions** | **LinkedIn mentions** | **Reddit mentions** | **Pinterest mentions** | **F1000 mentions** | **Q&A mentions** | **Video mentions** | **Syllabi mentions** | **Number of Mendeley readers** | **Number of Dimensions citations** |
| 1 | 2731 | 225 | 12 | 2 | 0 | 2845 | 0 | 0 | 6 | 3 | 0 | 0 | 0 | 0 | 0 | 0 | 1 | 0 | 599 | 379 |
| 2 | 2407 | 335 | 15 | 0 | 1 | 1198 | 0 | 0 | 12 | 17 | 0 | 0 | 1 | 0 | 1 | 0 | 0 | 0 | 354 | 86 |
| 3 | 1967 | 217 | 20 | 0 | 0 | 1294 | 0 | 0 | 23 | 2 | 5 | 0 | 0 | 0 | 0 | 0 | 1 | 0 | 354 | 319 |
| 4 | 1966 | 211 | 7 | 0 | 0 | 1435 | 0 | 0 | 11 | 0 | 0 | 0 | 6 | 0 | 0 | 0 | 2 | 0 | 265 | 270 |
| 5 | 1720 | 233 | 1 | 0 | 0 | 81 | 0 | 0 | 0 | 0 | 0 | 0 | 0 | 0 | 0 | 0 | 1 | 0 | 31 | 19 |
| 6 | 1674 | 233 | 0 | 0 | 0 | 3 | 0 | 0 | 0 | 0 | 0 | 0 | 0 | 0 | 0 | 0 | 0 | 0 | 31 | 40 |
| 7 | 1654 | 218 | 1 | 0 | 0 | 52 | 0 | 0 | 0 | 0 | 0 | 0 | 0 | 0 | 0 | 0 | 0 | 0 | 42 | 40 |
| 8 | 1502 | 330 | 4 | 0 | 0 | 225 | 0 | 0 | 1 | 0 | 0 | 0 | 0 | 0 | 0 | 0 | 0 | 0 | 42 | 4 |
| 9 | 1428 | 135 | 11 | 0 | 0 | 839 | 0 | 0 | 7 | 0 | 0 | 0 | 3 | 0 | 0 | 0 | 0 | 0 | 367 | 339 |
| 10 | 1358 | 63 | 11 | 0 | 0 | 2010 | 0 | 0 | 8 | 2 | 0 | 0 | 1 | 0 | 1 | 0 | 0 | 0 | 201 | 167 |
| 11 | 1343 | 39 | 13 | 0 | 0 | 1821 | 0 | 0 | 13 | 0 | 0 | 0 | 1 | 0 | 0 | 0 | 0 | 0 | 112 | 48 |
| 12 | 1327 | 63 | 6 | 1 | 0 | 1839 | 0 | 0 | 0 | 3 | 0 | 0 | 3 | 0 | 0 | 0 | 0 | 0 | 108 | 102 |
| 13 | 1259 | 347 | 6 | 0 | 7 | 200 | 0 | 0 | 13 | 1 | 3 | 0 | 0 | 0 | 0 | 0 | 13 | 0 | 490 | 215 |
| 14 | 1218 | 157 | 0 | 0 | 0 | 1 | 0 | 0 | 1 | 0 | 0 | 0 | 0 | 0 | 0 | 0 | 0 | 0 | 12 | 1 |
| 15 | 1170 | 219 | 0 | 0 | 0 | 253 | 0 | 0 | 1 | 0 | 0 | 0 | 0 | 0 | 0 | 0 | 0 | 0 | 41 | 4 |
| 16 | 1086 | 84 | 22 | 0 | 0 | 741 | 3 | 0 | 3 | 0 | 0 | 0 | 2 | 0 | 2 | 1 | 1 | 0 | 216 | 171 |
| 17 | 1054 | 105 | 1 | 0 | 0 | 331 | 0 | 0 | 1 | 0 | 0 | 0 | 0 | 0 | 0 | 0 | 0 | 0 | 104 | 8 |
| 18 | 1023 | 113 | 6 | 0 | 0 | 191 | 0 | 0 | 2 | 1 | 0 | 0 | 0 | 0 | 0 | 0 | 0 | 0 | 53 | 11 |
| 19 | 1008 | 216 | 9 | 0 | 0 | 137 | 0 | 0 | 15 | 0 | 0 | 0 | 0 | 0 | 0 | 0 | 4 | 0 | 464 | 247 |
| 20 | 971 | 117 | 0 | 0 | 0 | 144 | 0 | 0 | 0 | 0 | 0 | 0 | 0 | 0 | 0 | 0 | 0 | 0 | 35 | 10 |
| 21 | 955 | 49 | 4 | 2 | 7 | 1317 | 0 | 0 | 26 | 5 | 0 | 0 | 0 | 0 | 0 | 0 | 3 | 0 | 4527 | 2045 |
| 22 | 950 | 106 | 12 | 0 | 3 | 383 | 0 | 0 | 2 | 0 | 0 | 0 | 1 | 0 | 2 | 0 | 1 | 0 | 151 | 129 |
| 23 | 923 | 119 | 8 | 0 | 0 | 58 | 0 | 0 | 1 | 0 | 0 | 0 | 2 | 0 | 0 | 0 | 0 | 0 | 35 | 13 |
| 24 | 905 | 150 | 4 | 0 | 0 | 21 | 0 | 0 | 0 | 0 | 0 | 0 | 0 | 0 | 0 | 0 | 1 | 0 | 334 | 332 |
| 25 | 887 | 131 | 10 | 0 | 0 | 106 | 0 | 0 | 4 | 0 | 0 | 0 | 0 | 0 | 0 | 0 | 3 | 0 | 42 | 14 |
| 26 | 867 | 2 | 3 | 0 | 0 | 1558 | 0 | 0 | 0 | 0 | 0 | 0 | 0 | 0 | 0 | 0 | 0 | 0 | 35 | 21 |
| 27 | 862 | 85 | 12 | 8 | 1 | 192 | 0 | 0 | 8 | 0 | 1 | 0 | 2 | 0 | 0 | 0 | 0 | 0 | 643 | 967 |
| 28 | 829 | 35 | 4 | 3 | 0 | 1347 | 0 | 0 | 1 | 1 | 0 | 0 | 2 | 0 | 0 | 0 | 0 | 0 | 119 | 97 |
| 29 | 798 | 61 | 5 | 0 | 0 | 603 | 0 | 0 | 10 | 0 | 0 | 0 | 0 | 0 | 0 | 0 | 2 | 0 | 95 | 48 |
| 30 | 788 | 1 | 0 | 0 | 1 | 1263 | 0 | 0 | 3 | 0 | 0 | 0 | 0 | 0 | 0 | 0 | 0 | 0 | 203 | 79 |
| 31 | 743 | 91 | 4 | 0 | 2 | 64 | 0 | 0 | 7 | 0 | 0 | 0 | 0 | 0 | 1 | 0 | 3 | 0 | 127 | 75 |
| 32 | 736 | 24 | 6 | 0 | 0 | 1037 | 0 | 0 | 8 | 0 | 0 | 0 | 0 | 0 | 0 | 0 | 0 | 0 | 586 | 281 |
| 33 | 731 | 189 | 3 | 5 | 0 | 105 | 0 | 0 | 63 | 0 | 4 | 0 | 0 | 0 | 0 | 0 | 1 | 0 | 288 | 79 |
| 34 | 720 | 71 | 5 | 5 | 1 | 252 | 0 | 0 | 17 | 2 | 1 | 0 | 1 | 0 | 2 | 0 | 1 | 0 | 2232 | 1614 |
| 35 | 706 | 15 | 3 | 1 | 0 | 1097 | 0 | 0 | 0 | 0 | 0 | 0 | 2 | 0 | 0 | 0 | 1 | 0 | 157 | 66 |
| 36 | 695 | 1 | 2 | 0 | 0 | 1024 | 0 | 0 | 4 | 0 | 0 | 0 | 0 | 0 | 0 | 0 | 0 | 0 | 693 | 376 |
| 37 | 692 | 6 | 6 | 2 | 0 | 1330 | 0 | 0 | 4 | 2 | 0 | 0 | 1 | 0 | 0 | 0 | 0 | 0 | 288 | 227 |
| 38 | 681 | 75 | 1 | 0 | 0 | 169 | 0 | 0 | 2 | 0 | 0 | 0 | 0 | 0 | 0 | 0 | 0 | 0 | 61 | 33 |
| 39 | 674 | 38 | 2 | 1 | 0 | 595 | 0 | 0 | 24 | 0 | 0 | 0 | 0 | 0 | 0 | 0 | 4 | 0 | 177 | 37 |
| 40 | 672 | 4 | 1 | 0 | 0 | 1449 | 0 | 0 | 7 | 0 | 0 | 0 | 0 | 0 | 0 | 0 | 0 | 0 | 432 | 129 |
| 41 | 667 | 0 | 0 | 0 | 0 | 1930 | 0 | 0 | 2 | 0 | 0 | 0 | 0 | 0 | 0 | 0 | 0 | 0 | 3 | 1 |
| 42 | 658 | 74 | 0 | 0 | 0 | 117 | 0 | 0 | 6 | 0 | 0 | 0 | 0 | 0 | 0 | 0 | 0 | 0 | 153 | 150 |
| 43 | 649 | 66 | 5 | 0 | 0 | 293 | 0 | 0 | 0 | 0 | 0 | 0 | 0 | 0 | 0 | 0 | 0 | 0 | 63 | 24 |
| 44 | 644 | 74 | 0 | 6 | 0 | 6 | 0 | 0 | 2 | 7 | 0 | 0 | 0 | 0 | 0 | 0 | 0 | 0 | 175 | 113 |
| 45 | 643 | 71 | 1 | 0 | 1 | 2 | 0 | 0 | 0 | 0 | 0 | 0 | 0 | 0 | 0 | 0 | 0 | 0 | 130 | 74 |
| 46 | 641 | 109 | 1 | 0 | 0 | 8 | 0 | 0 | 0 | 0 | 0 | 0 | 0 | 0 | 0 | 0 | 0 | 0 | 77 | 29 |
| 47 | 637 | 70 | 0 | 0 | 0 | 34 | 0 | 0 | 36 | 0 | 0 | 0 | 0 | 0 | 0 | 0 | 0 | 0 | 159 | 76 |
| 48 | 635 | 75 | 0 | 0 | 0 | 5 | 0 | 0 | 0 | 0 | 0 | 0 | 0 | 0 | 0 | 0 | 0 | 0 | 25 | 21 |
| 49 | 632 | 97 | 1 | 0 | 0 | 40 | 0 | 0 | 1 | 0 | 0 | 0 | 0 | 0 | 0 | 0 | 0 | 0 | 15 | 7 |
| 50 | 627 | 51 | 3 | 0 | 0 | 263 | 0 | 0 | 2 | 0 | 0 | 0 | 0 | 0 | 0 | 0 | 0 | 0 | 55 | 25 |
| 51 | 622 | 60 | 6 | 0 | 0 | 235 | 0 | 0 | 0 | 0 | 0 | 0 | 0 | 0 | 0 | 0 | 0 | 0 | 45 | 76 |
| 52 | 620 | 37 | 1 | 1 | 0 | 435 | 0 | 0 | 4 | 0 | 0 | 0 | 0 | 0 | 0 | 0 | 1 | 0 | 113 | 149 |
| 53 | 618 | 7 | 3 | 2 | 0 | 1201 | 0 | 0 | 2 | 0 | 0 | 0 | 0 | 0 | 0 | 0 | 0 | 0 | 584 | 271 |
| 54 | 617 | 63 | 3 | 1 | 0 | 54 | 0 | 0 | 4 | 0 | 1 | 0 | 0 | 0 | 0 | 0 | 2 | 0 | 359 | 151 |
| 55 | 595 | 63 | 4 | 3 | 0 | 26 | 0 | 0 | 0 | 2 | 0 | 0 | 0 | 0 | 0 | 0 | 0 | 0 | 1064 | 526 |
| 56 | 580 | 1 | 4 | 1 | 0 | 955 | 0 | 0 | 5 | 0 | 0 | 0 | 0 | 0 | 0 | 0 | 0 | 0 | 903 | 773 |
| 57 | 569 | 56 | 7 | 6 | 0 | 581 | 0 | 0 | 2 | 0 | 0 | 0 | 0 | 0 | 0 | 0 | 0 | 0 | 192 | 281 |
| 58 | 559 | 20 | 1 | 2 | 0 | 785 | 0 | 0 | 2 | 2 | 0 | 0 | 0 | 0 | 0 | 0 | 0 | 0 | 181 | 123 |
| 59 | 555 | 64 | 0 | 0 | 0 | 7 | 0 | 0 | 0 | 0 | 0 | 0 | 0 | 0 | 0 | 0 | 0 | 0 | 122 | 33 |
| 60 | 551 | 11 | 5 | 0 | 0 | 865 | 0 | 0 | 3 | 0 | 0 | 0 | 0 | 0 | 0 | 0 | 0 | 0 | 36 | 15 |
| 61 | 551 | 75 | 0 | 0 | 0 | 8 | 0 | 0 | 0 | 0 | 0 | 0 | 0 | 0 | 0 | 0 | 0 | 0 | 67 | 27 |
| 62 | 549 | 3 | 0 | 0 | 0 | 1195 | 0 | 0 | 9 | 0 | 0 | 0 | 0 | 0 | 0 | 0 | 0 | 0 | 449 | 294 |
| 63 | 548 | 63 | 0 | 0 | 0 | 12 | 0 | 0 | 0 | 0 | 0 | 0 | 0 | 0 | 0 | 0 | 0 | 0 | 74 | 5 |
| 64 | 548 | 59 | 0 | 1 | 0 | 55 | 0 | 0 | 10 | 0 | 0 | 0 | 0 | 0 | 0 | 0 | 0 | 0 | 375 | 452 |
| 65 | 535 | 66 | 0 | 0 | 0 | 4 | 0 | 0 | 0 | 5 | 0 | 0 | 0 | 0 | 0 | 0 | 1 | 0 | 295 | 282 |
| 66 | 526 | 4 | 6 | 0 | 0 | 1637 | 0 | 0 | 1 | 0 | 0 | 0 | 0 | 0 | 1 | 0 | 0 | 0 | 95 | 90 |
| 67 | 526 | 77 | 6 | 0 | 0 | 20 | 0 | 0 | 0 | 0 | 0 | 0 | 0 | 0 | 0 | 0 | 2 | 0 | 842 | 279 |
| 68 | 522 | 65 | 0 | 0 | 0 | 10 | 0 | 0 | 0 | 0 | 0 | 0 | 0 | 0 | 0 | 0 | 0 | 0 | 5 | 20 |
| 69 | 522 | 61 | 0 | 0 | 0 | 42 | 0 | 0 | 3 | 0 | 0 | 0 | 0 | 0 | 0 | 0 | 0 | 0 | 71 | 68 |
| 70 | 517 | 70 | 0 | 0 | 0 | 0 | 0 | 0 | 0 | 0 | 0 | 0 | 0 | 0 | 0 | 0 | 0 | 0 | 4 | 6 |
| 71 | 517 | 0 | 0 | 0 | 0 | 722 | 0 | 0 | 6 | 0 | 0 | 0 | 0 | 0 | 0 | 0 | 0 | 0 | 10 | 17 |
| 72 | 515 | 317 | 0 | 0 | 0 | 5 | 0 | 0 | 3 | 0 | 0 | 0 | 0 | 0 | 0 | 0 | 0 | 0 | 38 | 11 |
| 73 | 515 | 104 | 0 | 0 | 0 | 117 | 0 | 0 | 0 | 0 | 0 | 0 | 0 | 0 | 0 | 0 | 0 | 0 | 13 | 16 |
| 74 | 513 | 65 | 6 | 0 | 0 | 17 | 0 | 0 | 3 | 0 | 0 | 0 | 0 | 0 | 0 | 0 | 3 | 0 | 226 | 240 |
| 75 | 508 | 66 | 1 | 0 | 0 | 2 | 0 | 0 | 0 | 0 | 0 | 0 | 0 | 0 | 0 | 0 | 0 | 0 | 57 | 20 |
| 76 | 505 | 121 | 0 | 0 | 0 | 4 | 0 | 0 | 1 | 0 | 0 | 0 | 0 | 0 | 0 | 0 | 0 | 0 | 20 | 8 |
| 77 | 504 | 61 | 0 | 0 | 1 | 6 | 0 | 0 | 1 | 0 | 0 | 0 | 0 | 0 | 0 | 0 | 0 | 0 | 71 | 59 |
| 78 | 502 | 5 | 1 | 1 | 0 | 1188 | 0 | 0 | 3 | 0 | 1 | 0 | 2 | 0 | 0 | 0 | 0 | 0 | 162 | 79 |
| 79 | 502 | 65 | 0 | 0 | 0 | 10 | 0 | 0 | 0 | 0 | 0 | 0 | 0 | 0 | 0 | 0 | 0 | 0 | 53 | 30 |
| 80 | 501 | 11 | 3 | 0 | 0 | 857 | 0 | 0 | 7 | 0 | 0 | 0 | 0 | 0 | 0 | 0 | 0 | 0 | 223 | 37 |
| 81 | 501 | 2 | 0 | 0 | 0 | 991 | 0 | 0 | 0 | 0 | 0 | 0 | 0 | 0 | 0 | 0 | 0 | 0 | 17 | 4 |
| 82 | 497 | 2 | 1 | 0 | 0 | 1063 | 0 | 0 | 6 | 0 | 0 | 0 | 0 | 0 | 0 | 0 | 0 | 0 | 239 | 101 |
| 83 | 497 | 57 | 0 | 0 | 0 | 8 | 0 | 0 | 1 | 0 | 0 | 0 | 0 | 0 | 0 | 0 | 0 | 0 | 139 | 10 |
| 84 | 495 | 0 | 1 | 0 | 0 | 903 | 0 | 0 | 14 | 2 | 0 | 0 | 0 | 0 | 0 | 0 | 0 | 0 | 71 | 14 |
| 85 | 495 | 55 | 1 | 1 | 0 | 4 | 0 | 0 | 0 | 0 | 0 | 0 | 0 | 0 | 0 | 0 | 0 | 0 | 149 | 119 |
| 86 | 488 | 0 | 3 | 0 | 0 | 1045 | 0 | 0 | 20 | 0 | 0 | 0 | 0 | 0 | 4 | 0 | 0 | 0 | 453 | 281 |
| 87 | 488 | 57 | 2 | 1 | 0 | 76 | 0 | 0 | 3 | 0 | 0 | 0 | 1 | 0 | 0 | 0 | 0 | 0 | 121 | 78 |
| 88 | 488 | 3 | 6 | 0 | 0 | 1054 | 0 | 0 | 1 | 0 | 0 | 0 | 0 | 0 | 0 | 0 | 1 | 0 | 134 | 105 |
| 89 | 479 | 55 | 0 | 0 | 0 | 3 | 0 | 0 | 1 | 0 | 0 | 0 | 0 | 0 | 0 | 0 | 0 | 0 | 66 | 26 |
| 90 | 479 | 52 | 0 | 8 | 0 | 51 | 0 | 0 | 0 | 0 | 0 | 0 | 0 | 0 | 0 | 0 | 0 | 0 | 39 | 24 |
| 91 | 477 | 44 | 15 | 0 | 2 | 76 | 0 | 0 | 6 | 5 | 0 | 0 | 1 | 0 | 0 | 1 | 2 | 0 | 242 | 199 |
| 92 | 471 | 49 | 2 | 0 | 0 | 36 | 0 | 0 | 1 | 0 | 0 | 0 | 0 | 0 | 0 | 0 | 0 | 0 | 202 | 37 |
| 93 | 471 | 41 | 8 | 0 | 0 | 253 | 0 | 0 | 19 | 0 | 0 | 0 | 1 | 0 | 1 | 0 | 2 | 0 | 282 | 129 |
| 94 | 470 | 52 | 0 | 0 | 2 | 12 | 0 | 0 | 2 | 0 | 0 | 0 | 0 | 0 | 0 | 0 | 0 | 0 | 56 | 48 |
| 95 | 468 | 45 | 1 | 0 | 0 | 222 | 0 | 0 | 1 | 0 | 0 | 0 | 0 | 0 | 0 | 0 | 0 | 0 | 29 | 12 |
| 96 | 465 | 1 | 3 | 0 | 0 | 691 | 0 | 0 | 13 | 3 | 0 | 0 | 0 | 0 | 1 | 0 | 0 | 0 | 886 | 440 |
| 97 | 465 | 111 | 0 | 0 | 2 | 3 | 0 | 0 | 0 | 0 | 0 | 0 | 0 | 0 | 0 | 0 | 0 | 0 | 111 | 53 |
| 98 | 460 | 58 | 1 | 0 | 2 | 2 | 0 | 0 | 0 | 0 | 0 | 0 | 0 | 0 | 0 | 0 | 0 | 0 | 66 | 44 |
| 99 | 459 | 129 | 0 | 0 | 0 | 37 | 0 | 0 | 3 | 0 | 0 | 0 | 0 | 0 | 0 | 0 | 0 | 0 | 261 | 172 |
| 100 | 456 | 59 | 0 | 0 | 0 | 4 | 0 | 0 | 0 | 0 | 0 | 0 | 0 | 0 | 0 | 0 | 0 | 0 | 127 | 84 |
| 101 | 455 | 53 | 0 | 0 | 0 | 0 | 0 | 0 | 0 | 0 | 0 | 0 | 0 | 0 | 0 | 0 | 0 | 0 | 60 | 56 |
| 102 | 455 | 0 | 13 | 1 | 0 | 715 | 0 | 0 | 16 | 0 | 1 | 0 | 0 | 0 | 1 | 0 | 1 | 0 | 1064 | 590 |
| 103 | 452 | 49 | 1 | 0 | 0 | 125 | 0 | 0 | 0 | 0 | 0 | 0 | 0 | 0 | 0 | 0 | 0 | 0 | 150 | 92 |
| 104 | 452 | 52 | 0 | 0 | 0 | 0 | 0 | 0 | 0 | 0 | 0 | 0 | 0 | 0 | 0 | 0 | 0 | 0 | 0 | 1 |
| 105 | 447 | 52 | 0 | 0 | 0 | 0 | 0 | 0 | 0 | 0 | 0 | 0 | 0 | 0 | 0 | 0 | 0 | 0 | 0 | 0 |
| 106 | 447 | 51 | 0 | 0 | 0 | 6 | 0 | 0 | 0 | 0 | 0 | 0 | 0 | 0 | 0 | 0 | 0 | 0 | 25 | 6 |
| 107 | 444 | 37 | 1 | 0 | 0 | 243 | 0 | 0 | 4 | 0 | 0 | 0 | 0 | 0 | 1 | 0 | 0 | 0 | 127 | 139 |
| 108 | 443 | 46 | 4 | 0 | 0 | 83 | 0 | 0 | 0 | 0 | 0 | 0 | 0 | 0 | 0 | 0 | 0 | 0 | 66 | 19 |
| 109 | 443 | 0 | 1 | 0 | 0 | 952 | 0 | 0 | 5 | 0 | 0 | 0 | 0 | 0 | 0 | 0 | 0 | 0 | 108 | 42 |
| 110 | 442 | 29 | 2 | 1 | 0 | 450 | 0 | 0 | 18 | 0 | 0 | 0 | 0 | 0 | 0 | 0 | 0 | 0 | 177 | 97 |
| 111 | 442 | 50 | 0 | 0 | 0 | 3 | 0 | 0 | 0 | 0 | 0 | 0 | 0 | 0 | 0 | 0 | 0 | 0 | 42 | 28 |
| 112 | 440 | 50 | 0 | 0 | 0 | 1 | 0 | 0 | 0 | 0 | 0 | 0 | 0 | 0 | 0 | 0 | 0 | 0 | 57 | 67 |
| 113 | 438 | 50 | 0 | 0 | 0 | 0 | 0 | 0 | 0 | 0 | 0 | 0 | 0 | 0 | 0 | 0 | 0 | 0 | 59 | 33 |
| 114 | 434 | 19 | 1 | 2 | 0 | 314 | 0 | 0 | 26 | 0 | 4 | 0 | 6 | 0 | 0 | 0 | 2 | 0 | 348 | 190 |
| 115 | 431 | 20 | 4 | 0 | 1 | 506 | 0 | 0 | 4 | 0 | 0 | 0 | 1 | 0 | 0 | 0 | 0 | 0 | 262 | 200 |
| 116 | 430 | 1 | 2 | 0 | 0 | 539 | 0 | 0 | 118 | 1 | 1 | 0 | 0 | 0 | 0 | 0 | 10 | 0 | 370 | 74 |
| 117 | 428 | 71 | 0 | 0 | 0 | 8 | 0 | 0 | 0 | 0 | 0 | 0 | 0 | 0 | 0 | 0 | 0 | 0 | 7 | 6 |
| 118 | 427 | 49 | 0 | 0 | 0 | 0 | 0 | 0 | 0 | 0 | 0 | 0 | 0 | 0 | 0 | 0 | 0 | 0 | 23 | 9 |
| 119 | 426 | 47 | 0 | 1 | 0 | 1 | 0 | 0 | 2 | 0 | 0 | 0 | 0 | 0 | 1 | 0 | 0 | 0 | 99 | 102 |
| 120 | 426 | 3 | 1 | 1 | 0 | 811 | 0 | 0 | 4 | 0 | 0 | 0 | 0 | 0 | 0 | 0 | 0 | 0 | 307 | 159 |
| 121 | 426 | 17 | 5 | 3 | 0 | 490 | 0 | 0 | 19 | 3 | 0 | 0 | 0 | 0 | 2 | 0 | 0 | 0 | 411 | 331 |
| 122 | 424 | 49 | 0 | 0 | 0 | 4 | 0 | 0 | 0 | 0 | 0 | 0 | 0 | 0 | 0 | 0 | 0 | 0 | 10 | 5 |
| 123 | 422 | 42 | 2 | 2 | 0 | 32 | 0 | 0 | 2 | 0 | 0 | 0 | 0 | 0 | 0 | 0 | 0 | 0 | 128 | 76 |
| 124 | 419 | 15 | 3 | 0 | 0 | 538 | 0 | 0 | 2 | 0 | 0 | 0 | 1 | 0 | 1 | 0 | 0 | 0 | 242 | 90 |
| 125 | 418 | 36 | 4 | 0 | 0 | 177 | 0 | 0 | 6 | 0 | 0 | 0 | 0 | 0 | 0 | 0 | 0 | 0 | 106 | 81 |
| 126 | 416 | 30 | 0 | 1 | 0 | 314 | 0 | 0 | 4 | 1 | 0 | 0 | 0 | 0 | 0 | 0 | 0 | 0 | 251 | 89 |
| 127 | 413 | 49 | 3 | 0 | 3 | 129 | 0 | 0 | 14 | 0 | 1 | 0 | 0 | 0 | 0 | 0 | 7 | 0 | 367 | 183 |
| 128 | **409** | 22 | 5 | 1 | 0 | 414 | 0 | 0 | 0 | 0 | 0 | 0 | 0 | 0 | 0 | 0 | 0 | 0 | 43 | 65 |
| 129 | **409** | 65 | 3 | 0 | 0 | 25 | 0 | 0 | 1 | 0 | 0 | 0 | 1 | 0 | 0 | 0 | 0 | 0 | 7 | 2 |
| 130 | **408** | 44 | 3 | 0 | 0 | 6 | 0 | 0 | 0 | 0 | 1 | 0 | 0 | 0 | 0 | 0 | 0 | 0 | 68 | 24 |
| 131 | **405** | 31 | 1 | 8 | 0 | 161 | 0 | 0 | 6 | 0 | 0 | 0 | 0 | 0 | 0 | 1 | 1 | 0 | 976 | 502 |
| 132 | **404** | 49 | 0 | 0 | 0 | 9 | 0 | 0 | 0 | 0 | 0 | 0 | 0 | 0 | 0 | 0 | 0 | 0 | 0 | 1 |
| 133 | **404** | 74 | 1 | 0 | 0 | 13 | 0 | 0 | 0 | 0 | 0 | 0 | 0 | 0 | 0 | 0 | 0 | 0 | 3 | 2 |
| 134 | **403** | 41 | 3 | 0 | 0 | 29 | 0 | 0 | 0 | 1 | 0 | 0 | 0 | 0 | 0 | 0 | 0 | 0 | 186 | 163 |
| 135 | **402** | 46 | 3 | 0 | 0 | 23 | 0 | 0 | 0 | 0 | 0 | 0 | 6 | 0 | 0 | 0 | 0 | 0 | 31 | 4 |
| 136 | **399** | 0 | 1 | 0 | 0 | 492 | 0 | 0 | 2 | 0 | 0 | 0 | 0 | 0 | 0 | 0 | 0 | 0 | 10 | 5 |
| 137 | **399** | 45 | 0 | 0 | 0 | 3 | 0 | 0 | 0 | 0 | 0 | 0 | 0 | 0 | 0 | 0 | 0 | 0 | 19 | 13 |
| 138 | **399** | 42 | 3 | 0 | 0 | 56 | 0 | 0 | 0 | 0 | 0 | 0 | 0 | 0 | 0 | 0 | 0 | 0 | 77 | 45 |
| 139 | **397** | 45 | 2 | 0 | 0 | 51 | 0 | 0 | 27 | 0 | 1 | 0 | 0 | 0 | 0 | 0 | 1 | 0 | 79 | 11 |
| 140 | **396** | 301 | 0 | 0 | 0 | 4 | 0 | 0 | 0 | 2 | 0 | 0 | 0 | 0 | 0 | 0 | 0 | 0 | 31 | 7 |
| 141 | **396** | 51 | 1 | 0 | 0 | 167 | 0 | 0 | 0 | 0 | 0 | 0 | 0 | 0 | 0 | 0 | 0 | 0 | 140 | 20 |
| 142 | **393** | 16 | 1 | 0 | 0 | 500 | 0 | 0 | 9 | 0 | 0 | 0 | 0 | 0 | 1 | 0 | 0 | 0 | 575 | 464 |
| 143 | **391** | 44 | 0 | 0 | 0 | 0 | 0 | 0 | 0 | 0 | 0 | 0 | 0 | 0 | 0 | 0 | 0 | 0 | 70 | 27 |
| 144 | **389** | 13 | 2 | 0 | 0 | 524 | 0 | 0 | 0 | 0 | 0 | 0 | 0 | 0 | 0 | 0 | 0 | 0 | 142 | 95 |
| 145 | **389** | 45 | 5 | 1 | 0 | 154 | 0 | 0 | 27 | 0 | 4 | 0 | 0 | 0 | 0 | 0 | 0 | 0 | 294 | 230 |
| 146 | **388** | 0 | 1 | 0 | 0 | 821 | 0 | 0 | 8 | 0 | 0 | 0 | 0 | 0 | 0 | 0 | 0 | 0 | 154 | 40 |
| 147 | **386** | 43 | 1 | 0 | 0 | 103 | 0 | 0 | 8 | 0 | 0 | 0 | 0 | 0 | 0 | 0 | 1 | 0 | 132 | 58 |
| 148 | **385** | 42 | 2 | 0 | 0 | 56 | 0 | 0 | 8 | 0 | 0 | 0 | 0 | 0 | 1 | 0 | 0 | 0 | 26 | 31 |
| 149 | 384 | 0 | 1 | 1 | 0 | 770 | 0 | 0 | 9 | 5 | 0 | 0 | 0 | 0 | 0 | 0 | 0 | 0 | 347 | 180 |
| 150 | 382 | 48 | 2 | 0 | 0 | 3 | 0 | 0 | 0 | 0 | 0 | 0 | 0 | 0 | 0 | 0 | 1 | 0 | 62 | 52 |
| 151 | 381 | 27 | 4 | 0 | 0 | 481 | 0 | 0 | 0 | 0 | 0 | 0 | 0 | 0 | 0 | 0 | 0 | 0 | 16 | 8 |
| 152 | 380 | 46 | 0 | 0 | 0 | 28 | 0 | 0 | 0 | 0 | 0 | 0 | 0 | 0 | 0 | 0 | 0 | 0 | 5 | 3 |
| 153 | 378 | 45 | 1 | 0 | 0 | 8 | 0 | 0 | 0 | 0 | 0 | 0 | 0 | 0 | 0 | 0 | 0 | 0 | 37 | 5 |
| 154 | 378 | 36 | 1 | 0 | 0 | 130 | 0 | 0 | 9 | 0 | 0 | 0 | 0 | 0 | 0 | 0 | 0 | 0 | 40 | 24 |
| 155 | 377 | 40 | 1 | 1 | 0 | 60 | 0 | 0 | 7 | 0 | 1 | 0 | 0 | 0 | 1 | 0 | 0 | 0 | 708 | 300 |
| 156 | 375 | 32 | 2 | 0 | 0 | 206 | 0 | 0 | 2 | 0 | 0 | 0 | 0 | 0 | 0 | 0 | 0 | 0 | 144 | 78 |
| 157 | 374 | 45 | 2 | 0 | 0 | 12 | 0 | 0 | 0 | 0 | 0 | 0 | 0 | 0 | 0 | 0 | 0 | 0 | 42 | 5 |
| 158 | 373 | 0 | 1 | 0 | 0 | 785 | 0 | 0 | 5 | 0 | 0 | 0 | 0 | 0 | 0 | 0 | 0 | 0 | 227 | 85 |
| 159 | 372 | 39 | 2 | 1 | 0 | 148 | 0 | 0 | 0 | 0 | 0 | 0 | 1 | 0 | 0 | 0 | 0 | 0 | 24 | 34 |
| 160 | 371 | 45 | 1 | 1 | 0 | 145 | 0 | 0 | 0 | 0 | 0 | 0 | 0 | 0 | 0 | 0 | 0 | 0 | 148 | 154 |
| 161 | 370 | 41 | 0 | 0 | 0 | 9 | 0 | 0 | 4 | 0 | 2 | 0 | 0 | 0 | 0 | 0 | 0 | 0 | 26 | 24 |
| 162 | 369 | 35 | 7 | 0 | 0 | 22 | 0 | 0 | 0 | 0 | 0 | 0 | 0 | 0 | 0 | 0 | 1 | 0 | 53 | 24 |
| 163 | 367 | 38 | 3 | 0 | 0 | 66 | 0 | 0 | 3 | 1 | 0 | 0 | 1 | 0 | 0 | 0 | 0 | 0 | 224 | 106 |
| 164 | 367 | 44 | 0 | 0 | 0 | 0 | 0 | 0 | 0 | 0 | 0 | 0 | 0 | 0 | 0 | 0 | 0 | 0 | 14 | 5 |
| 165 | 367 | 40 | 3 | 0 | 0 | 155 | 0 | 0 | 0 | 0 | 0 | 0 | 1 | 0 | 0 | 0 | 0 | 0 | 158 | 119 |
| 166 | 366 | 41 | 2 | 0 | 0 | 28 | 0 | 0 | 2 | 0 | 0 | 0 | 0 | 0 | 0 | 0 | 0 | 0 | 30 | 12 |
| 167 | 366 | 2 | 0 | 0 | 0 | 817 | 0 | 0 | 2 | 0 | 0 | 0 | 0 | 0 | 0 | 0 | 0 | 0 | 31 | 13 |
| 168 | 363 | 0 | 1 | 0 | 0 | 545 | 0 | 0 | 1 | 0 | 0 | 0 | 0 | 0 | 0 | 0 | 0 | 0 | 120 | 27 |
| 169 | 361 | 43 | 2 | 1 | 1 | 56 | 0 | 0 | 1 | 0 | 0 | 0 | 0 | 0 | 0 | 0 | 0 | 0 | 596 | 231 |
| 170 | 361 | 0 | 0 | 0 | 0 | 694 | 0 | 0 | 0 | 0 | 0 | 0 | 0 | 0 | 0 | 0 | 0 | 0 | 82 | 29 |
| 171 | 361 | 37 | 2 | 0 | 0 | 0 | 0 | 0 | 0 | 0 | 0 | 0 | 0 | 0 | 0 | 0 | 0 | 0 | 24 | 22 |
| 172 | 360 | 42 | 1 | 0 | 0 | 16 | 0 | 0 | 0 | 0 | 0 | 0 | 0 | 0 | 0 | 0 | 0 | 0 | 30 | 27 |
| 173 | 359 | 0 | 3 | 0 | 0 | 857 | 0 | 0 | 2 | 0 | 0 | 0 | 0 | 0 | 2 | 0 | 0 | 0 | 85 | 25 |
| 174 | 355 | 37 | 2 | 0 | 0 | 1 | 0 | 0 | 0 | 0 | 0 | 0 | 0 | 0 | 0 | 0 | 0 | 0 | 84 | 83 |
| 175 | 354 | 5 | 1 | 0 | 0 | 494 | 0 | 0 | 40 | 0 | 0 | 0 | 0 | 0 | 0 | 0 | 0 | 0 | 186 | 88 |
| 176 | 354 | 0 | 2 | 0 | 0 | 604 | 0 | 0 | 11 | 0 | 0 | 0 | 0 | 0 | 0 | 0 | 0 | 0 | 166 | 104 |
| 177 | 353 | 12 | 1 | 0 | 0 | 454 | 0 | 0 | 3 | 0 | 0 | 0 | 0 | 0 | 2 | 0 | 0 | 0 | 45 | 16 |
| 178 | 353 | 36 | 2 | 0 | 0 | 50 | 0 | 0 | 3 | 0 | 0 | 0 | 0 | 0 | 0 | 0 | 0 | 0 | 73 | 29 |
| 179 | 350 | 0 | 0 | 0 | 0 | 539 | 0 | 0 | 1 | 0 | 0 | 0 | 0 | 0 | 0 | 0 | 0 | 0 | 42 | 4 |
| 180 | 350 | 35 | 3 | 0 | 0 | 24 | 0 | 0 | 11 | 0 | 1 | 0 | 0 | 0 | 0 | 0 | 0 | 0 | 140 | 131 |
| 181 | 349 | 0 | 0 | 0 | 0 | 658 | 0 | 0 | 1 | 0 | 0 | 0 | 0 | 0 | 0 | 0 | 0 | 0 | 81 | 30 |
| 182 | 347 | 40 | 2 | 0 | 0 | 42 | 0 | 0 | 0 | 0 | 0 | 0 | 0 | 0 | 0 | 0 | 0 | 0 | 19 | 21 |
| 183 | 347 | 20 | 3 | 0 | 0 | 284 | 0 | 0 | 0 | 0 | 0 | 0 | 0 | 0 | 0 | 0 | 0 | 0 | 45 | 20 |
| 184 | 345 | 35 | 2 | 0 | 0 | 23 | 0 | 0 | 3 | 0 | 0 | 0 | 0 | 0 | 0 | 0 | 2 | 0 | 143 | 64 |
| 185 | 344 | 5 | 3 | 0 | 0 | 504 | 0 | 0 | 0 | 0 | 0 | 0 | 1 | 0 | 0 | 0 | 0 | 0 | 578 | 289 |
| 186 | 343 | 13 | 3 | 8 | 5 | 291 | 0 | 0 | 3 | 13 | 0 | 0 | 0 | 0 | 0 | 0 | 0 | 0 | 2532 | 1814 |
| 187 | 340 | 32 | 2 | 0 | 1 | 141 | 0 | 0 | 8 | 4 | 0 | 0 | 0 | 0 | 0 | 0 | 0 | 0 | 384 | 243 |
| 188 | 340 | 39 | 0 | 0 | 0 | 2 | 0 | 0 | 7 | 0 | 0 | 0 | 0 | 0 | 0 | 0 | 0 | 0 | 108 | 10 |
| 189 | 340 | 0 | 0 | 0 | 0 | 705 | 0 | 0 | 0 | 0 | 0 | 0 | 0 | 0 | 0 | 0 | 0 | 0 | 12 | 5 |
| 190 | 340 | 0 | 0 | 0 | 0 | 547 | 0 | 0 | 0 | 0 | 0 | 0 | 0 | 0 | 0 | 0 | 0 | 0 | 192 | 6 |
| 191 | 338 | 39 | 0 | 0 | 0 | 2 | 0 | 0 | 0 | 0 | 0 | 0 | 0 | 0 | 0 | 0 | 0 | 0 | 54 | 26 |
| 192 | 338 | 36 | 0 | 0 | 0 | 62 | 0 | 0 | 0 | 0 | 0 | 0 | 0 | 0 | 0 | 0 | 0 | 0 | 81 | 54 |
| 193 | 336 | 0 | 2 | 0 | 0 | 738 | 0 | 0 | 6 | 0 | 0 | 0 | 0 | 0 | 0 | 0 | 0 | 0 | 79 | 18 |
| 194 | 334 | 34 | 1 | 0 | 0 | 71 | 0 | 0 | 1 | 0 | 0 | 0 | 0 | 0 | 0 | 0 | 0 | 0 | 28 | 19 |
| 195 | 333 | 38 | 2 | 0 | 0 | 39 | 0 | 0 | 0 | 0 | 0 | 0 | 1 | 0 | 0 | 0 | 0 | 0 | 19 | 8 |
| 196 | 331 | 27 | 5 | 0 | 0 | 110 | 0 | 0 | 5 | 0 | 0 | 0 | 0 | 0 | 0 | 0 | 0 | 0 | 193 | 127 |
| 197 | 330 | 5 | 5 | 0 | 0 | 647 | 0 | 0 | 0 | 0 | 0 | 0 | 0 | 0 | 0 | 0 | 1 | 0 | 255 | 43 |
| 198 | 327 | 37 | 0 | 0 | 0 | 26 | 0 | 0 | 4 | 0 | 0 | 0 | 0 | 0 | 0 | 0 | 0 | 0 | 21 | 12 |
| 199 | 327 | 38 | 0 | 0 | 0 | 12 | 0 | 0 | 0 | 0 | 0 | 0 | 0 | 0 | 0 | 0 | 0 | 0 | 13 | 11 |
| 200 | 323 | 2 | 4 | 0 | 0 | 670 | 0 | 0 | 3 | 0 | 0 | 0 | 0 | 0 | 0 | 0 | 0 | 0 | 52 | 32 |
| 201 | 320 | 0 | 0 | 0 | 0 | 430 | 0 | 0 | 9 | 0 | 0 | 0 | 0 | 0 | 0 | 0 | 0 | 0 | 7 | 4 |
| 202 | 320 | 29 | 1 | 0 | 0 | 106 | 0 | 0 | 5 | 0 | 0 | 0 | 0 | 0 | 0 | 0 | 0 | 0 | 136 | 70 |
| 203 | 319 | 36 | 0 | 0 | 0 | 21 | 0 | 0 | 0 | 0 | 0 | 0 | 0 | 0 | 0 | 0 | 0 | 0 | 9 | 10 |
| 204 | 318 | 38 | 1 | 0 | 0 | 1 | 0 | 0 | 0 | 0 | 0 | 0 | 0 | 0 | 0 | 0 | 0 | 0 | 9 | 6 |
| 205 | 317 | 0 | 0 | 0 | 0 | 603 | 0 | 0 | 6 | 0 | 0 | 0 | 0 | 0 | 0 | 0 | 0 | 0 | 18 | 0 |
| 206 | 316 | 0 | 1 | 0 | 0 | 549 | 0 | 0 | 6 | 0 | 0 | 0 | 0 | 0 | 0 | 0 | 0 | 0 | 92 | 54 |
| 207 | 314 | 34 | 2 | 2 | 0 | 41 | 0 | 0 | 2 | 0 | 0 | 0 | 0 | 0 | 0 | 0 | 0 | 0 | 94 | 48 |
| 208 | 313 | 3 | 0 | 1 | 0 | 469 | 0 | 0 | 8 | 0 | 0 | 0 | 0 | 0 | 1 | 0 | 0 | 0 | 191 | 292 |
| 209 | 312 | 34 | 1 | 0 | 0 | 4 | 0 | 0 | 1 | 1 | 0 | 0 | 0 | 0 | 0 | 0 | 0 | 0 | 127 | 19 |
| 210 | 312 | 1 | 1 | 3 | 0 | 662 | 0 | 0 | 0 | 0 | 0 | 0 | 0 | 0 | 0 | 0 | 0 | 0 | 2350 | 871 |
| 211 | 311 | 0 | 3 | 0 | 0 | 577 | 0 | 0 | 7 | 0 | 0 | 0 | 0 | 0 | 0 | 0 | 0 | 0 | 173 | 165 |
| 212 | 311 | 43 | 0 | 0 | 0 | 29 | 0 | 0 | 15 | 0 | 1 | 0 | 0 | 0 | 0 | 0 | 0 | 0 | 156 | 9 |
| 213 | 311 | 0 | 2 | 0 | 0 | 489 | 0 | 0 | 1 | 0 | 2 | 0 | 0 | 0 | 0 | 0 | 0 | 0 | 225 | 83 |
| 214 | 310 | 35 | 1 | 0 | 0 | 25 | 0 | 0 | 2 | 0 | 0 | 0 | 0 | 0 | 0 | 0 | 0 | 0 | 51 | 65 |
| 215 | 310 | 0 | 0 | 0 | 0 | 382 | 0 | 0 | 1 | 0 | 0 | 0 | 0 | 0 | 0 | 0 | 0 | 0 | 33 | 5 |
| 216 | 310 | 32 | 2 | 1 | 2 | 126 | 0 | 0 | 10 | 0 | 0 | 0 | 1 | 0 | 0 | 0 | 0 | 0 | 220 | 57 |
| 217 | 309 | 34 | 2 | 1 | 0 | 50 | 0 | 0 | 19 | 1 | 0 | 0 | 0 | 0 | 0 | 0 | 0 | 0 | 59 | 23 |
| 218 | 307 | 1 | 2 | 0 | 0 | 443 | 0 | 0 | 24 | 0 | 0 | 0 | 0 | 0 | 0 | 0 | 0 | 0 | 162 | 21 |
| 219 |  | 35 | 0 | 0 | 0 | 2 | 0 | 0 | 0 | 0 | 0 | 0 | 0 | 0 | 0 | 0 | 0 | 0 | 13 | 13 |
| 220 | 305 | 37 | 0 | 0 | 0 | 0 | 0 | 0 | 0 | 0 | 0 | 0 | 0 | 0 | 0 | 0 | 0 | 0 | 9 | 0 |
| 221 | 305 | 0 | 0 | 0 | 0 | 517 | 0 | 0 | 0 | 0 | 0 | 0 | 0 | 0 | 0 | 0 | 0 | 0 | 10 | 5 |
| 222 | 305 | 37 | 0 | 0 | 0 | 6 | 0 | 0 | 2 | 0 | 0 | 0 | 0 | 0 | 0 | 0 | 0 | 0 | 2 | 1 |
| 223 | 304 | 32 | 3 | 0 | 0 | 19 | 0 | 0 | 5 | 0 | 0 | 0 | 0 | 0 | 0 | 0 | 0 | 0 | 89 | 33 |
| 224 | 304 | 0 | 0 | 0 | 0 | 561 | 0 | 0 | 2 | 0 | 0 | 0 | 0 | 0 | 0 | 0 | 1 | 0 | 111 | 46 |
| 225 | 303 | 35 | 0 | 0 | 0 | 0 | 0 | 0 | 0 | 0 | 0 | 0 | 0 | 0 | 0 | 0 | 0 | 0 | 20 | 23 |
| 3012226 | 302 | 0 | 3 | 0 | 0 | 495 | 0 | 0 | 4 | 1 | 0 | 0 | 0 | 0 | 0 | 0 | 0 | 0 | 107 | 10 |
| 227 | 302 | 38 | 0 | 0 | 0 | 11 | 0 | 0 | 4 | 0 | 0 | 0 | 0 | 0 | 0 | 0 | 0 | 0 | 76 | 20 |
| 228 | 298 | 38 | 4 | 0 | 0 | 11 | 0 | 0 | 1 | 0 | 0 | 0 | 0 | 0 | 0 | 0 | 0 | 0 | 154 | 171 |
| 229 | 298 | 0 | 1 | 0 | 0 | 489 | 0 | 0 | 5 | 0 | 0 | 0 | 0 | 0 | 0 | 0 | 0 | 0 | 73 | 22 |
| 230 | 296 | 36 | 2 | 0 | 0 | 15 | 0 | 0 | 4 | 0 | 0 | 0 | 0 | 0 | 0 | 0 | 0 | 0 | 7 | 13 |
| 231 | 296 | 0 | 4 | 0 | 0 | 405 | 0 | 0 | 19 | 0 | 0 | 0 | 0 | 0 | 0 | 0 | 0 | 0 | 1319 | 541 |
| 232 | 296 | 33 | 1 | 0 | 0 | 1 | 0 | 0 | 0 | 0 | 0 | 0 | 0 | 0 | 0 | 0 | 1 | 0 | 108 | 59 |
| 233 | 295 | 51 | 2 | 3 | 0 | 84 | 0 | 0 | 0 | 6 | 0 | 0 | 0 | 0 | 0 | 0 | 0 | 0 | 132 | 94 |
| 234 | 295 | 32 | 0 | 0 | 0 | 45 | 0 | 0 | 2 | 0 | 0 | 0 | 0 | 0 | 0 | 0 | 0 | 0 | 80 | 79 |
| 235 | 294 | 0 | 3 | 0 | 0 | 536 | 0 | 0 | 3 | 0 | 0 | 0 | 0 | 0 | 0 | 0 | 0 | 0 | 25 | 15 |
| 236 | 294 | 68 | 0 | 0 | 0 | 0 | 0 | 0 | 0 | 0 | 0 | 0 | 0 | 0 | 0 | 0 | 0 | 0 | 2 | 1 |
| 237 | 291 | 68 | 0 | 0 | 0 | 0 | 0 | 0 | 0 | 0 | 0 | 0 | 0 | 0 | 0 | 0 | 0 | 0 | 32 | 1 |
| 238 | 290 | 22 | 2 | 0 | 0 | 181 | 0 | 0 | 4 | 0 | 0 | 0 | 0 | 0 | 0 | 0 | 0 | 0 | 48 | 18 |
| 239 | 289 | 31 | 0 | 0 | 0 | 26 | 0 | 0 | 4 | 0 | 0 | 0 | 0 | 0 | 0 | 0 | 0 | 0 | 103 | 29 |
| 240 | 288 | 34 | 0 | 0 | 0 | 1 | 0 | 0 | 0 | 0 | 0 | 0 | 0 | 0 | 0 | 0 | 0 | 0 | 82 | 37 |
| 241 | 288 | 4 | 0 | 0 | 0 | 311 | 0 | 0 | 3 | 2 | 1 | 0 | 0 | 0 | 0 | 0 | 0 | 0 | 26 | 19 |
| 242 | 288 | 4 | 16 | 1 | 0 | 375 | 0 | 0 | 7 | 0 | 3 | 0 | 0 | 0 | 1 | 0 | 0 | 0 | 414 | 638 |
| 243 | 287 | 0 | 4 | 0 | 0 | 466 | 0 | 0 | 8 | 0 | 0 | 0 | 0 | 0 | 0 | 0 | 0 | 0 | 249 | 89 |
| 244 | 286 | 0 | 0 | 0 | 0 | 505 | 0 | 0 | 3 | 0 | 0 | 0 | 0 | 0 | 0 | 0 | 0 | 0 | 58 | 39 |
| 245 | 283 | 21 | 2 | 0 | 0 | 253 | 0 | 0 | 17 | 0 | 1 | 0 | 0 | 0 | 0 | 0 | 0 | 0 | 154 | 27 |
| 246 | 283 | 30 | 1 | 0 | 0 | 51 | 0 | 0 | 6 | 0 | 0 | 0 | 0 | 0 | 0 | 0 | 0 | 0 | 52 | 10 |
| 247 | 283 | 33 | 0 | 0 | 0 | 9 | 0 | 0 | 1 | 0 | 0 | 0 | 2 | 0 | 0 | 0 | 0 | 0 | 16 | 13 |
| 248 | 282 | 0 | 1 | 0 | 0 | 762 | 0 | 0 | 10 | 0 | 5 | 0 | 0 | 0 | 0 | 0 | 0 | 0 | 79 | 38 |
| 249 | 282 | 30 | 1 | 0 | 1 | 16 | 0 | 0 | 0 | 3 | 0 | 0 | 0 | 0 | 0 | 0 | 0 | 0 | 63 | 28 |
| 250 | 281 | 19 | 3 | 0 | 0 | 262 | 0 | 0 | 1 | 0 | 0 | 0 | 0 | 0 | 0 | 1 | 0 | 0 | 19 | 7 |
| 251 | 280 | 0 | 2 | 0 | 0 | 424 | 0 | 0 | 14 | 0 | 0 | 0 | 0 | 0 | 0 | 0 | 0 | 0 | 162 | 37 |
| 252 | 280 | 56 | 0 | 0 | 0 | 2 | 0 | 0 | 0 | 1 | 0 | 0 | 0 | 0 | 0 | 0 | 0 | 0 | 24 | 4 |
| 253 | 280 | 5 | 1 | 3 | 0 | 414 | 0 | 0 | 4 | 3 | 0 | 0 | 0 | 0 | 0 | 0 | 0 | 0 | 1053 | 529 |
| 254 | 279 | 23 | 1 | 0 | 0 | 178 | 0 | 0 | 3 | 0 | 0 | 0 | 0 | 0 | 0 | 0 | 1 | 0 | 122 | 23 |
| 255 | 277 | 66 | 0 | 0 | 0 | 1 | 0 | 0 | 0 | 0 | 0 | 0 | 0 | 0 | 0 | 0 | 0 | 0 | 5 | 2 |
| 256 | 275 | 18 | 2 | 0 | 0 | 239 | 0 | 0 | 5 | 0 | 0 | 0 | 0 | 0 | 0 | 0 | 1 | 0 | 131 | 50 |
| 257 | 274 | 0 | 2 | 0 | 0 | 425 | 0 | 0 | 4 | 0 | 0 | 0 | 0 | 0 | 0 | 0 | 0 | 0 | 187 | 80 |
| 258 | 274 | 33 | 0 | 0 | 0 | 0 | 0 | 0 | 0 | 0 | 0 | 0 | 0 | 0 | 0 | 0 | 0 | 0 | 0 | 0 |
| 259 | 273 | 0 | 0 | 0 | 0 | 425 | 0 | 0 | 0 | 0 | 0 | 0 | 0 | 0 | 0 | 0 | 0 | 0 | 42 | 8 |
| 260 | 273 | 0 | 1 | 0 | 0 | 455 | 0 | 0 | 1 | 0 | 0 | 0 | 1 | 0 | 0 | 0 | 0 | 0 | 44 | 10 |
| 261 | 273 | 16 | 5 | 0 | 0 | 171 | 0 | 0 | 1 | 0 | 0 | 0 | 0 | 0 | 0 | 0 | 0 | 0 | 30 | 12 |
| 262 | 272 | 1 | 1 | 2 | 2 | 500 | 0 | 0 | 9 | 1 | 0 | 0 | 0 | 0 | 0 | 0 | 0 | 0 | 595 | 601 |
| 263 | 272 | 30 | 1 | 0 | 0 | 26 | 0 | 0 | 1 | 0 | 0 | 0 | 0 | 0 | 0 | 0 | 0 | 0 | 7 | 7 |
| 264 | 270 | 3 | 2 | 1 | 1 | 426 | 1 | 0 | 5 | 0 | 0 | 0 | 0 | 0 | 0 | 0 | 0 | 0 | 1377 | 804 |
| 265 | 270 | 30 | 2 | 1 | 0 | 70 | 0 | 0 | 6 | 0 | 0 | 0 | 0 | 0 | 1 | 0 | 0 | 0 | 197 | 373 |
| 266 | 270 | 31 | 0 | 0 | 0 | 14 | 0 | 0 | 3 | 0 | 0 | 0 | 0 | 0 | 0 | 0 | 0 | 0 | 2 | 1 |
| 267 | 269 | 4 | 4 | 1 | 0 | 656 | 0 | 0 | 2 | 0 | 0 | 0 | 0 | 0 | 0 | 0 | 0 | 0 | 112 | 112 |
| 268 | 268 | 30 | 2 | 0 | 0 | 20 | 0 | 0 | 1 | 0 | 0 | 0 | 0 | 0 | 0 | 0 | 0 | 0 | 1 | 0 |
| 269 | 267 | 25 | 3 | 2 | 0 | 132 | 0 | 0 | 7 | 0 | 1 | 0 | 0 | 0 | 1 | 0 | 0 | 0 | 131 | 244 |
| 270 | 267 | 31 | 0 | 0 | 0 | 0 | 0 | 0 | 0 | 0 | 0 | 0 | 0 | 0 | 0 | 0 | 0 | 0 | 4 | 0 |
| 271 | 267 | 5 | 2 | 0 | 0 | 415 | 0 | 0 | 4 | 0 | 0 | 0 | 0 | 0 | 0 | 0 | 0 | 0 | 84 | 36 |
| 272 | 266 | 6 | 2 | 0 | 0 | 324 | 0 | 0 | 1 | 0 | 0 | 0 | 0 | 0 | 0 | 0 | 0 | 0 | 88 | 38 |
| 273 | 266 | 0 | 0 | 0 | 0 | 475 | 0 | 0 | 4 | 0 | 0 | 0 | 0 | 0 | 0 | 0 | 0 | 0 | 159 | 44 |
| 274 | 265 | 23 | 1 | 1 | 1 | 31 | 0 | 0 | 35 | 0 | 2 | 0 | 1 | 0 | 0 | 0 | 1 | 0 | 413 | 145 |
| 275 | 264 | 1 | 0 | 1 | 0 | 424 | 0 | 0 | 10 | 1 | 0 | 0 | 0 | 0 | 0 | 0 | 1 | 0 | 267 | 131 |
| 276 | 264 | 0 | 0 | 0 | 0 | 376 | 0 | 0 | 2 | 0 | 0 | 0 | 0 | 0 | 0 | 0 | 0 | 0 | 10 | 8 |
| 277 | 263 | 18 | 3 | 0 | 0 | 193 | 0 | 0 | 0 | 0 | 0 | 0 | 0 | 0 | 0 | 0 | 0 | 0 | 13 | 4 |
| 278 | 262 | 32 | 0 | 0 | 0 | 11 | 0 | 0 | 1 | 0 | 0 | 0 | 0 | 0 | 0 | 0 | 0 | 0 | 12 | 25 |
| 279 | 260 | 12 | 0 | 0 | 0 | 293 | 0 | 0 | 3 | 1 | 0 | 0 | 0 | 0 | 0 | 0 | 0 | 0 | 82 | 49 |
| 280 | 259 | 5 | 6 | 1 | 0 | 404 | 0 | 0 | 2 | 0 | 0 | 0 | 0 | 0 | 0 | 0 | 0 | 0 | 139 | 74 |
| 281 | 259 | 0 | 0 | 0 | 0 | 427 | 0 | 0 | 6 | 0 | 0 | 0 | 0 | 0 | 0 | 0 | 0 | 0 | 59 | 1 |
| 282 | 259 | 0 | 0 | 0 | 0 | 290 | 0 | 0 | 0 | 0 | 0 | 0 | 0 | 0 | 0 | 0 | 0 | 0 | 28 | 4 |
| 283 | 259 | 4 | 3 | 0 | 0 | 410 | 0 | 0 | 0 | 0 | 0 | 0 | 0 | 0 | 0 | 0 | 0 | 0 | 27 | 24 |
| 284 | 259 | 0 | 3 | 0 | 0 | 419 | 0 | 0 | 0 | 0 | 0 | 0 | 0 | 0 | 0 | 0 | 0 | 0 | 40 | 16 |
| 285 | 257 | 27 | 3 | 0 | 0 | 97 | 0 | 0 | 4 | 0 | 0 | 0 | 0 | 0 | 0 | 0 | 0 | 0 | 352 | 265 |
| 286 | 257 | 1 | 4 | 1 | 0 | 397 | 0 | 0 | 3 | 0 | 0 | 0 | 0 | 0 | 0 | 0 | 0 | 0 | 159 | 106 |
| 287 | 257 | 27 | 1 | 0 | 0 | 33 | 0 | 0 | 1 | 0 | 0 | 0 | 0 | 0 | 0 | 0 | 0 | 0 | 20 | 4 |
| 288 | 256 | 0 | 0 | 1 | 0 | 363 | 0 | 0 | 9 | 0 | 2 | 0 | 0 | 0 | 0 | 0 | 0 | 0 | 167 | 102 |
| 289 | 256 | 7 | 1 | 1 | 0 | 308 | 0 | 0 | 21 | 6 | 0 | 0 | 0 | 0 | 0 | 0 | 0 | 0 | 541 | 315 |
| 290 | 256 | 26 | 1 | 0 | 0 | 25 | 0 | 0 | 3 | 0 | 0 | 0 | 2 | 0 | 0 | 0 | 0 | 0 | 102 | 81 |
| 291 | 255 | 4 | 1 | 0 | 0 | 364 | 0 | 0 | 0 | 0 | 0 | 0 | 0 | 0 | 0 | 0 | 0 | 0 | 243 | 107 |
| 292 | 255 | 7 | 2 | 2 | 0 | 225 | 0 | 0 | 12 | 0 | 0 | 0 | 0 | 0 | 0 | 0 | 2 | 0 | 540 | 159 |
| 293 | 254 | 17 | 3 | 0 | 0 | 195 | 0 | 0 | 1 | 0 | 0 | 0 | 0 | 0 | 0 | 0 | 0 | 0 | 12 | 8 |
| 294 | 253 | 1 | 2 | 0 | 0 | 440 | 0 | 0 | 5 | 0 | 0 | 0 | 0 | 0 | 0 | 0 | 0 | 0 | 86 | 35 |
| 295 | 253 | 30 | 0 | 0 | 0 | 35 | 0 | 0 | 3 | 0 | 0 | 0 | 0 | 0 | 1 | 0 | 0 | 0 | 297 | 383 |
| 296 | 252 | 1 | 2 | 1 | 0 | 460 | 0 | 0 | 0 | 0 | 0 | 0 | 0 | 0 | 0 | 0 | 0 | 0 | 49 | 21 |
| 297 | 251 | 3 | 1 | 0 | 0 | 413 | 0 | 0 | 0 | 0 | 0 | 0 | 0 | 0 | 0 | 0 | 0 | 0 | 53 | 22 |
| 298 | 250 | 25 | 5 | 0 | 0 | 45 | 0 | 0 | 4 | 0 | 0 | 0 | 0 | 0 | 0 | 0 | 0 | 0 | 153 | 96 |
| 299 | 250 | 0 | 1 | 0 | 0 | 380 | 0 | 0 | 4 | 0 | 0 | 0 | 0 | 0 | 0 | 0 | 0 | 0 | 213 | 79 |
| 300 | 249 | 25 | 1 | 0 | 0 | 42 | 0 | 0 | 3 | 0 | 0 | 0 | 0 | 0 | 0 | 0 | 0 | 0 | 7 | 4 |
| 301 | 249 | 0 | 1 | 0 | 0 | 379 | 0 | 0 | 8 | 0 | 0 | 0 | 0 | 0 | 0 | 0 | 0 | 0 | 177 | 45 |
| 302 | 248 | 14 | 1 | 0 | 1 | 163 | 0 | 0 | 8 | 1 | 0 | 0 | 0 | 0 | 1 | 0 | 1 | 0 | 924 | 799 |
| 303 | 247 | 19 | 6 | 0 | 0 | 83 | 0 | 0 | 1 | 0 | 0 | 0 | 0 | 0 | 0 | 0 | 0 | 0 | 37 | 24 |
| 304 | 247 | 3 | 0 | 0 | 0 | 744 | 0 | 0 | 0 | 0 | 0 | 0 | 0 | 0 | 0 | 0 | 0 | 0 | 91 | 63 |
| 305 | 247 | 0 | 1 | 0 | 0 | 371 | 0 | 0 | 5 | 0 | 2 | 0 | 0 | 0 | 0 | 0 | 0 | 0 | 149 | 52 |
| 306 | 247 | 23 | 3 | 0 | 0 | 135 | 0 | 0 | 0 | 0 | 0 | 0 | 0 | 0 | 0 | 0 | 0 | 0 | 113 | 24 |
| 307 | 246 | 26 | 1 | 0 | 0 | 33 | 0 | 0 | 2 | 0 | 0 | 0 | 0 | 0 | 0 | 0 | 0 | 0 | 68 | 91 |
| 308 | 245 | 8 | 3 | 0 | 0 | 276 | 0 | 0 | 4 | 0 | 0 | 0 | 0 | 0 | 0 | 0 | 0 | 0 | 806 | 206 |
| 309 | 244 | 42 | 0 | 0 | 0 | 11 | 0 | 0 | 3 | 0 | 0 | 0 | 0 | 0 | 0 | 0 | 0 | 0 | 55 | 22 |
| 310 | 244 | 2 | 5 | 0 | 0 | 483 | 0 | 0 | 1 | 0 | 0 | 0 | 0 | 0 | 0 | 0 | 0 | 0 | 144 | 37 |
| 311 | 244 | 25 | 0 | 0 | 0 | 10 | 0 | 0 | 0 | 0 | 0 | 0 | 0 | 0 | 0 | 0 | 0 | 0 | 129 | 33 |
| 312 | 243 | 97 | 0 | 0 | 0 | 0 | 0 | 0 | 0 | 0 | 0 | 0 | 0 | 0 | 0 | 0 | 0 | 0 | 10 | 2 |
| 313 | 243 | 10 | 0 | 0 | 0 | 272 | 0 | 0 | 0 | 0 | 0 | 0 | 5 | 0 | 0 | 0 | 0 | 0 | 102 | 23 |
| 314 | 243 | 9 | 4 | 0 | 0 | 315 | 0 | 0 | 1 | 0 | 0 | 0 | 0 | 0 | 1 | 0 | 0 | 0 | 73 | 26 |
| 315 | 243 | 93 | 0 | 0 | 0 | 20 | 0 | 0 | 1 | 0 | 0 | 0 | 0 | 0 | 0 | 0 | 0 | 0 | 112 | 88 |
| 316 | 242 | 26 | 0 | 0 | 0 | 1 | 0 | 0 | 0 | 0 | 0 | 0 | 0 | 0 | 0 | 0 | 0 | 0 | 28 | 14 |
| 317 | 242 | 26 | 3 | 3 | 0 | 40 | 0 | 0 | 3 | 0 | 0 | 0 | 0 | 0 | 0 | 0 | 1 | 0 | 70 | 44 |
| 318 | 241 | 29 | 1 | 4 | 0 | 58 | 0 | 0 | 3 | 0 | 0 | 0 | 0 | 0 | 1 | 0 | 2 | 0 | 1922 | 890 |
| 319 | 241 | 2 | 2 | 1 | 0 | 398 | 0 | 0 | 8 | 0 | 1 | 0 | 0 | 0 | 0 | 0 | 0 | 0 | 151 | 151 |
| 320 | 241 | 21 | 0 | 0 | 0 | 90 | 0 | 0 | 3 | 1 | 0 | 0 | 0 | 0 | 0 | 0 | 0 | 0 | 63 | 26 |
| 321 | 241 | 15 | 0 | 1 | 0 | 319 | 0 | 0 | 0 | 0 | 0 | 0 | 0 | 0 | 0 | 0 | 0 | 0 | 20 | 15 |
| 322 | 240 | 22 | 0 | 0 | 0 | 87 | 0 | 0 | 3 | 0 | 0 | 0 | 0 | 0 | 0 | 0 | 0 | 0 | 18 | 4 |
| 323 | 239 | 29 | 1 | 0 | 0 | 8 | 0 | 0 | 1 | 0 | 0 | 0 | 0 | 0 | 0 | 0 | 0 | 0 | 16 | 6 |
| 324 | 239 | 20 | 1 | 0 | 0 | 131 | 0 | 0 | 0 | 0 | 0 | 0 | 0 | 0 | 0 | 0 | 0 | 0 | 218 | 166 |
| 325 | 238 | 0 | 1 | 0 | 0 | 389 | 0 | 0 | 1 | 0 | 0 | 0 | 0 | 0 | 0 | 0 | 0 | 0 | 186 | 133 |
| 326 | 238 | 15 | 1 | 2 | 0 | 210 | 0 | 0 | 0 | 0 | 0 | 0 | 0 | 0 | 0 | 0 | 0 | 0 | 89 | 62 |
| 327 | 236 | 0 | 1 | 0 | 0 | 348 | 0 | 0 | 4 | 0 | 0 | 0 | 0 | 0 | 0 | 0 | 0 | 0 | 117 | 27 |
| 328 | 236 | 10 | 2 | 1 | 0 | 287 | 0 | 0 | 3 | 1 | 0 | 0 | 0 | 0 | 0 | 0 | 0 | 0 | 189 | 78 |
| 329 | 236 | 0 | 0 | 0 | 0 | 405 | 0 | 0 | 10 | 0 | 0 | 0 | 0 | 0 | 0 | 0 | 0 | 0 | 108 | 50 |
| 330 | 235 | 8 | 4 | 0 | 0 | 174 | 0 | 0 | 65 | 3 | 5 | 0 | 2 | 0 | 0 | 0 | 4 | 0 | 233 | 49 |
| 331 | 235 | 0 | 0 | 25 | 0 | 310 | 0 | 0 | 6 | 0 | 0 | 0 | 0 | 0 | 0 | 0 | 0 | 0 | 831 | 274 |
| 332 | 234 | 24 | 0 | 0 | 0 | 1 | 0 | 0 | 0 | 0 | 0 | 0 | 0 | 0 | 0 | 0 | 0 | 0 | 113 | 70 |
| 333 | 233 | 2 | 2 | 1 | 0 | 438 | 0 | 0 | 4 | 0 | 0 | 0 | 0 | 0 | 0 | 0 | 1 | 0 | 62 | 44 |
| 334 | 233 | 11 | 5 | 0 | 0 | 362 | 0 | 0 | 0 | 0 | 0 | 0 | 0 | 0 | 0 | 0 | 0 | 0 | 41 | 31 |
| 335 | 232 | 0 | 2 | 0 | 0 | 408 | 0 | 0 | 3 | 0 | 0 | 0 | 0 | 0 | 1 | 0 | 0 | 0 | 208 | 122 |
| 336 | 232 | 0 | 3 | 0 | 0 | 247 | 0 | 0 | 0 | 0 | 0 | 0 | 0 | 0 | 2 | 0 | 0 | 0 | 31 | 4 |
| 337 | 232 | 22 | 6 | 0 | 1 | 41 | 0 | 0 | 12 | 0 | 0 | 0 | 1 | 0 | 0 | 0 | 0 | 0 | 325 | 193 |
| 338 | 230 | 0 | 7 | 0 | 0 | 279 | 0 | 0 | 1 | 0 | 0 | 0 | 0 | 0 | 0 | 0 | 0 | 0 | 135 | 53 |
| 339 | 228 | 24 | 0 | 0 | 0 | 19 | 0 | 0 | 1 | 0 | 0 | 0 | 0 | 0 | 0 | 0 | 0 | 0 | 32 | 11 |
| 340 | 227 | 0 | 0 | 0 | 0 | 274 | 0 | 0 | 6 | 0 | 0 | 0 | 0 | 0 | 0 | 0 | 0 | 0 | 22 | 0 |
| 341 | 226 | 14 | 0 | 0 | 0 | 156 | 0 | 0 | 4 | 0 | 0 | 0 | 0 | 0 | 0 | 0 | 0 | 0 | 132 | 50 |
| 342 | 226 | 0 | 1 | 0 | 0 | 347 | 0 | 0 | 0 | 0 | 0 | 0 | 0 | 0 | 0 | 0 | 0 | 0 | 19 | 13 |
| 343 | 225 | 0 | 0 | 0 | 0 | 557 | 0 | 0 | 3 | 0 | 0 | 0 | 0 | 0 | 0 | 0 | 0 | 0 | 67 | 14 |
| 344 | 224 | 22 | 0 | 0 | 0 | 66 | 0 | 0 | 0 | 0 | 0 | 0 | 0 | 0 | 0 | 0 | 0 | 0 | 165 | 93 |
| 345 | 223 | 24 | 2 | 0 | 3 | 24 | 0 | 0 | 8 | 4 | 1 | 0 | 0 | 0 | 0 | 0 | 6 | 0 | 260 | 66 |
| 346 | 223 | 28 | 0 | 0 | 0 | 0 | 0 | 0 | 0 | 0 | 0 | 0 | 0 | 0 | 0 | 0 | 0 | 0 | 4 | 1 |
| 347 | 223 | 0 | 19 | 3 | 0 | 262 | 0 | 0 | 17 | 0 | 0 | 0 | 1 | 0 | 1 | 0 | 0 | 0 | 1623 | 1616 |
| 348 | 222 | 4 | 0 | 0 | 0 | 269 | 0 | 0 | 1 | 0 | 0 | 0 | 0 | 0 | 0 | 0 | 2 | 0 | 327 | 59 |
| 349 | 222 | 1 | 1 | 0 | 0 | 352 | 0 | 0 | 2 | 0 | 0 | 0 | 0 | 0 | 3 | 0 | 0 | 0 | 151 | 139 |
| 350 | 222 | 0 | 0 | 0 | 0 | 381 | 0 | 0 | 1 | 0 | 0 | 0 | 0 | 0 | 0 | 0 | 0 | 0 | 33 | 8 |
| 351 | 221 | 23 | 2 | 4 | 0 | 16 | 0 | 0 | 3 | 1 | 0 | 0 | 0 | 0 | 0 | 0 | 0 | 0 | 105 | 30 |
| 352 | 221 | 1 | 4 | 0 | 0 | 425 | 0 | 0 | 0 | 0 | 0 | 0 | 0 | 0 | 0 | 0 | 0 | 0 | 60 | 35 |
| 353 | 221 | 0 | 1 | 0 | 0 | 337 | 0 | 0 | 5 | 0 | 0 | 0 | 0 | 0 | 0 | 0 | 0 | 0 | 110 | 57 |
| 354 | 220 | 0 | 1 | 0 | 0 | 317 | 0 | 0 | 9 | 0 | 1 | 0 | 0 | 0 | 1 | 0 | 0 | 0 | 175 | 120 |
| 355 | 220 | 7 | 3 | 0 | 1 | 236 | 0 | 0 | 2 | 0 | 0 | 0 | 0 | 0 | 0 | 0 | 2 | 0 | 108 | 38 |
| 356 | 220 | 25 | 1 | 0 | 0 | 50 | 0 | 0 | 2 | 0 | 0 | 0 | 0 | 0 | 0 | 0 | 0 | 0 | 6 | 1 |
| 357 | 219 | 69 | 0 | 0 | 0 | 48 | 0 | 0 | 24 | 0 | 0 | 0 | 0 | 0 | 0 | 0 | 0 | 0 | 30 | 85 |
| 358 | 219 | 23 | 0 | 0 | 0 | 2 | 0 | 0 | 0 | 0 | 0 | 0 | 0 | 0 | 0 | 0 | 0 | 0 | 13 | 0 |
| 359 | 219 | 13 | 1 | 0 | 0 | 141 | 0 | 0 | 20 | 0 | 0 | 0 | 0 | 0 | 1 | 0 | 0 | 0 | 194 | 112 |
| 360 | 218 | 13 | 0 | 0 | 0 | 155 | 0 | 0 | 8 | 0 | 0 | 0 | 0 | 0 | 0 | 0 | 0 | 0 | 68 | 12 |
| 361 | 218 | 4 | 1 | 0 | 0 | 452 | 0 | 0 | 1 | 0 | 0 | 0 | 0 | 0 | 0 | 0 | 0 | 0 | 27 | 39 |
| 362 | 218 | 25 | 0 | 0 | 0 | 36 | 0 | 0 | 0 | 0 | 0 | 0 | 0 | 0 | 0 | 0 | 0 | 0 | 35 | 8 |
| 363 | 216 | 0 | 3 | 0 | 0 | 328 | 0 | 0 | 0 | 0 | 0 | 0 | 0 | 0 | 0 | 0 | 0 | 0 | 104 | 29 |
| 364 | 215 | 0 | 0 | 0 | 0 | 248 | 0 | 0 | 2 | 0 | 0 | 0 | 0 | 0 | 0 | 0 | 0 | 0 | 10 | 1 |
| 365 | 215 | 25 | 0 | 0 | 0 | 18 | 0 | 0 | 0 | 0 | 0 | 0 | 0 | 0 | 0 | 0 | 0 | 0 | 115 | 103 |
| 366 | 215 | 1 | 5 | 0 | 0 | 315 | 0 | 0 | 9 | 0 | 0 | 0 | 0 | 0 | 0 | 0 | 0 | 0 | 215 | 94 |
| 367 | 214 | 0 | 1 | 0 | 0 | 356 | 0 | 0 | 1 | 0 | 0 | 0 | 0 | 0 | 0 | 0 | 0 | 0 | 269 | 138 |
| 368 | 213 | 0 | 4 | 0 | 0 | 305 | 0 | 0 | 1 | 0 | 0 | 0 | 0 | 0 | 0 | 0 | 0 | 0 | 31 | 9 |
| 369 | 213 | 42 | 0 | 0 | 0 | 13 | 0 | 0 | 11 | 0 | 2 | 0 | 0 | 0 | 0 | 0 | 2 | 0 | 31 | 16 |
| 370 | 212 | 3 | 3 | 0 | 0 | 297 | 0 | 0 | 13 | 0 | 1 | 0 | 0 | 0 | 0 | 0 | 1 | 0 | 196 | 33 |
| 371 | 212 | 16 | 0 | 0 | 0 | 222 | 0 | 0 | 14 | 0 | 0 | 0 | 0 | 0 | 0 | 0 | 0 | 0 | 117 | 38 |
| 372 | 212 | 3 | 0 | 1 | 0 | 259 | 0 | 0 | 6 | 0 | 0 | 0 | 0 | 0 | 0 | 0 | 1 | 0 | 848 | 560 |
| 373 | 212 | 1 | 2 | 0 | 0 | 331 | 0 | 0 | 1 | 0 | 0 | 0 | 0 | 0 | 0 | 0 | 0 | 0 | 120 | 51 |
| 374 | 212 | 1 | 0 | 0 | 0 | 261 | 0 | 0 | 0 | 0 | 0 | 0 | 0 | 0 | 0 | 0 | 0 | 0 | 131 | 66 |
| 375 | 211 | 0 | 0 | 0 | 0 | 298 | 0 | 0 | 0 | 0 | 0 | 0 | 0 | 0 | 0 | 0 | 0 | 0 | 12 | 4 |
| 376 | 211 | 1 | 1 | 0 | 0 | 310 | 0 | 0 | 2 | 0 | 0 | 0 | 0 | 0 | 0 | 0 | 0 | 0 | 52 | 28 |
| 377 | 211 | 23 | 1 | 0 | 0 | 18 | 0 | 0 | 0 | 0 | 0 | 0 | 0 | 0 | 0 | 0 | 0 | 0 | 11 | 0 |
| 378 | 211 | 15 | 2 | 4 | 0 | 183 | 0 | 0 | 9 | 0 | 0 | 0 | 0 | 0 | 0 | 0 | 0 | 0 | 89 | 50 |
| 379 | 211 | 0 | 0 | 0 | 0 | 306 | 0 | 0 | 3 | 0 | 0 | 0 | 0 | 0 | 0 | 0 | 0 | 0 | 98 | 28 |
| 380 | 210 | 0 | 2 | 0 | 0 | 436 | 0 | 0 | 1 | 0 | 0 | 0 | 0 | 0 | 0 | 0 | 0 | 0 | 15 | 14 |
| 381 | 210 | 0 | 1 | 0 | 1 | 327 | 0 | 0 | 1 | 0 | 0 | 0 | 0 | 0 | 0 | 0 | 0 | 0 | 145 | 28 |
| 382 | 210 | 25 | 0 | 0 | 0 | 20 | 0 | 0 | 0 | 0 | 0 | 0 | 0 | 0 | 0 | 0 | 0 | 0 | 43 | 16 |
| 383 | 209 | 0 | 0 | 0 | 0 | 290 | 0 | 0 | 1 | 0 | 0 | 0 | 0 | 0 | 0 | 0 | 0 | 0 | 4 | 0 |
| 384 | 209 | 2 | 1 | 0 | 0 | 295 | 0 | 0 | 3 | 0 | 0 | 0 | 0 | 0 | 0 | 0 | 0 | 0 | 29 | 15 |
| 385 | 208 | 1 | 0 | 0 | 0 | 297 | 0 | 0 | 9 | 0 | 0 | 0 | 0 | 0 | 0 | 0 | 0 | 0 | 61 | 39 |
| 386 | 206 | 8 | 0 | 0 | 0 | 242 | 0 | 0 | 5 | 0 | 0 | 0 | 0 | 0 | 0 | 0 | 1 | 0 | 221 | 35 |
| 387 | 206 | 26 | 0 | 0 | 0 | 205 | 0 | 0 | 1 | 0 | 0 | 0 | 0 | 0 | 0 | 0 | 0 | 0 | 25 | 10 |
| 388 | 206 | 0 | 2 | 0 | 0 | 313 | 0 | 0 | 2 | 0 | 0 | 0 | 0 | 0 | 0 | 0 | 0 | 0 | 66 | 23 |
| 389 | 205 | 61 | 0 | 0 | 0 | 4 | 0 | 0 | 1 | 0 | 0 | 0 | 0 | 0 | 0 | 0 | 0 | 0 | 191 | 116 |
| 390 | 205 | 14 | 2 | 0 | 0 | 155 | 0 | 0 | 0 | 0 | 0 | 0 | 0 | 0 | 0 | 0 | 0 | 0 | 19 | 11 |
| 391 | 204 | 44 | 0 | 0 | 0 | 2 | 0 | 0 | 0 | 0 | 0 | 0 | 0 | 0 | 0 | 0 | 0 | 0 | 64 | 13 |
| 392 | 203 | 0 | 1 | 0 | 0 | 346 | 0 | 0 | 3 | 0 | 0 | 0 | 0 | 0 | 0 | 0 | 0 | 0 | 129 | 33 |
| 393 | 203 | 2 | 0 | 1 | 0 | 287 | 0 | 0 | 5 | 0 | 0 | 0 | 0 | 0 | 0 | 0 | 0 | 0 | 192 | 107 |
| 394 | 202 | 21 | 0 | 0 | 0 | 5 | 0 | 0 | 0 | 0 | 0 | 0 | 0 | 0 | 0 | 0 | 0 | 0 | 35 | 14 |
| 395 | 202 | 32 | 0 | 0 | 0 | 5 | 0 | 0 | 0 | 0 | 0 | 0 | 0 | 0 | 0 | 0 | 0 | 0 | 30 | 9 |
| 396 | 202 | 10 | 2 | 0 | 0 | 152 | 0 | 0 | 1 | 0 | 0 | 0 | 0 | 0 | 0 | 0 | 2 | 0 | 154 | 82 |
| 397 | 201 | 2 | 0 | 0 | 0 | 262 | 0 | 0 | 0 | 0 | 0 | 0 | 0 | 0 | 0 | 0 | 0 | 0 | 95 | 59 |
| 398 | 201 | 19 | 3 | 0 | 0 | 11 | 0 | 0 | 3 | 0 | 0 | 0 | 0 | 0 | 0 | 0 | 0 | 0 | 71 | 27 |
| 399 | 200 | 20 | 2 | 1 | 0 | 44 | 0 | 0 | 3 | 0 | 0 | 0 | 0 | 0 | 0 | 0 | 0 | 0 | 165 | 104 |
| 400 | 200 | 16 | 7 | 0 | 0 | 4 | 0 | 0 | 0 | 0 | 0 | 0 | 0 | 0 | 0 | 0 | 0 | 0 | 161 | 110 |
| 401 | 200 | 34 | 0 | 0 | 0 | 0 | 0 | 0 | 0 | 0 | 0 | 0 | 0 | 0 | 0 | 0 | 0 | 0 | 8 | 1 |
| 402 | 200 | 0 | 2 | 0 | 0 | 341 | 0 | 0 | 8 | 1 | 0 | 0 | 0 | 0 | 0 | 0 | 0 | 0 | 54 | 58 |
| 403 | 198 | 47 | 0 | 0 | 0 | 19 | 0 | 0 | 0 | 0 | 0 | 0 | 0 | 0 | 0 | 0 | 0 | 0 | 128 | 67 |
| 404 | 198 | 0 | 1 | 0 | 0 | 619 | 0 | 0 | 1 | 0 | 0 | 0 | 0 | 0 | 0 | 0 | 0 | 0 | 82 | 91 |
| 405 | 198 | 0 | 0 | 0 | 0 | 270 | 0 | 0 | 5 | 0 | 0 | 0 | 0 | 0 | 0 | 0 | 0 | 0 | 40 | 17 |
| 406 | 197 | 16 | 9 | 0 | 0 | 62 | 0 | 0 | 2 | 0 | 0 | 0 | 0 | 0 | 0 | 0 | 0 | 0 | 32 | 26 |
| 407 | 197 | 0 | 0 | 0 | 0 | 323 | 0 | 0 | 1 | 0 | 0 | 0 | 0 | 0 | 0 | 0 | 0 | 0 | 30 | 16 |
| 408 | 196 | 4 | 0 | 0 | 0 | 265 | 0 | 0 | 9 | 0 | 0 | 0 | 0 | 0 | 1 | 0 | 0 | 0 | 251 | 137 |
| 409 | 196 | 22 | 2 | 1 | 0 | 24 | 0 | 0 | 0 | 0 | 0 | 0 | 0 | 0 | 0 | 0 | 0 | 0 | 67 | 36 |
| 410 | 195 | 15 | 1 | 1 | 0 | 121 | 0 | 0 | 1 | 0 | 0 | 0 | 0 | 0 | 0 | 0 | 0 | 0 | 42 | 17 |
| 411 | 195 | 12 | 1 | 10 | 0 | 108 | 0 | 0 | 0 | 0 | 0 | 0 | 0 | 0 | 0 | 0 | 0 | 0 | 309 | 402 |
| 412 | 194 | 0 | 4 | 0 | 0 | 243 | 0 | 0 | 7 | 0 | 3 | 0 | 0 | 0 | 0 | 0 | 0 | 0 | 69 | 16 |
| 413 | 194 | 7 | 1 | 0 | 0 | 190 | 0 | 0 | 7 | 6 | 1 | 0 | 0 | 0 | 0 | 0 | 0 | 0 | 70 | 41 |
| 414 | 193 | 4 | 0 | 1 | 0 | 199 | 0 | 0 | 2 | 0 | 0 | 0 | 0 | 0 | 0 | 0 | 1 | 0 | 342 | 134 |
| 415 | 193 | 0 | 3 | 0 | 0 | 235 | 0 | 0 | 1 | 0 | 0 | 0 | 0 | 0 | 0 | 0 | 0 | 0 | 127 | 67 |
| 416 | 193 | 1 | 13 | 0 | 0 | 181 | 0 | 0 | 11 | 0 | 1 | 0 | 0 | 0 | 0 | 0 | 0 | 0 | 184 | 72 |
| 417 | 192 | 0 | 3 | 0 | 0 | 278 | 0 | 0 | 6 | 0 | 0 | 0 | 0 | 0 | 0 | 0 | 0 | 0 | 14 | 0 |
| 418 | 192 | 21 | 1 | 0 | 0 | 36 | 0 | 0 | 1 | 0 | 0 | 0 | 0 | 0 | 2 | 0 | 0 | 0 | 228 | 157 |
| 419 | 192 | 20 | 1 | 0 | 0 | 60 | 0 | 0 | 4 | 0 | 0 | 0 | 0 | 0 | 0 | 0 | 2 | 0 | 48 | 27 |
| 420 | 192 | 0 | 1 | 3 | 0 | 302 | 0 | 0 | 8 | 0 | 0 | 0 | 0 | 0 | 0 | 0 | 0 | 0 | 203 | 201 |
| 421 | 192 | 15 | 4 | 1 | 0 | 79 | 0 | 0 | 1 | 0 | 0 | 0 | 0 | 0 | 0 | 0 | 0 | 0 | 15 | 14 |
| 422 | 191 | 0 | 0 | 0 | 0 | 262 | 0 | 0 | 11 | 0 | 0 | 0 | 0 | 0 | 0 | 0 | 0 | 0 | 1 | 2 |
| 423 | 191 | 3 | 2 | 1 | 0 | 232 | 0 | 0 | 2 | 0 | 0 | 0 | 0 | 0 | 0 | 0 | 0 | 0 | 311 | 141 |
| 424 | 191 | 16 | 0 | 0 | 0 | 83 | 0 | 0 | 1 | 0 | 0 | 0 | 0 | 0 | 0 | 0 | 3 | 0 | 59 | 32 |
| 425 | 191 | 1 | 0 | 0 | 0 | 253 | 0 | 0 | 0 | 0 | 0 | 0 | 0 | 0 | 0 | 0 | 0 | 0 | 5 | 2 |
| 426 | 191 | 13 | 2 | 0 | 0 | 134 | 0 | 0 | 3 | 0 | 0 | 0 | 0 | 0 | 0 | 0 | 0 | 0 | 80 | 57 |
| 427 | 190 | 0 | 0 | 0 | 0 | 324 | 0 | 0 | 5 | 0 | 0 | 0 | 0 | 0 | 0 | 0 | 0 | 0 | 168 | 47 |
| 428 | 190 | 1 | 0 | 0 | 0 | 290 | 0 | 0 | 0 | 0 | 0 | 0 | 0 | 0 | 0 | 0 | 0 | 0 | 77 | 17 |
| 429 | 189 | 3 | 3 | 0 | 0 | 239 | 0 | 0 | 6 | 0 | 0 | 0 | 0 | 0 | 0 | 0 | 0 | 0 | 340 | 213 |
| 430 | 187 | 0 | 0 | 0 | 0 | 310 | 0 | 0 | 0 | 0 | 0 | 0 | 0 | 0 | 0 | 0 | 0 | 0 | 33 | 14 |
| 431 | 187 | 1 | 1 | 0 | 0 | 279 | 0 | 0 | 5 | 0 | 0 | 0 | 0 | 0 | 1 | 0 | 0 | 0 | 139 | 96 |
| 432 | 185 | 5 | 6 | 1 | 0 | 255 | 0 | 0 | 0 | 0 | 0 | 0 | 0 | 0 | 0 | 0 | 0 | 0 | 57 | 50 |
| 433 | 185 | 22 | 0 | 0 | 0 | 15 | 0 | 0 | 1 | 0 | 0 | 0 | 0 | 0 | 0 | 0 | 0 | 0 | 101 | 73 |
| 434 | 185 | 15 | 4 | 0 | 0 | 99 | 0 | 0 | 1 | 0 | 0 | 0 | 0 | 0 | 0 | 1 | 4 | 0 | 174 | 142 |
| 435 | 184 | 17 | 1 | 0 | 2 | 168 | 0 | 0 | 2 | 0 | 0 | 0 | 0 | 0 | 0 | 0 | 0 | 0 | 209 | 175 |
| 436 | 184 | 19 | 0 | 0 | 0 | 2 | 0 | 0 | 0 | 0 | 0 | 0 | 0 | 0 | 0 | 0 | 0 | 0 | 152 | 82 |
| 437 | 184 | 0 | 2 | 0 | 0 | 306 | 0 | 0 | 11 | 0 | 0 | 0 | 0 | 0 | 0 | 0 | 0 | 0 | 104 | 87 |
| 438 | 183 | 0 | 1 | 0 | 0 | 308 | 0 | 0 | 6 | 0 | 0 | 0 | 0 | 0 | 0 | 0 | 0 | 0 | 93 | 48 |
| 439 | 183 | 10 | 3 | 0 | 0 | 190 | 0 | 0 | 1 | 0 | 0 | 0 | 0 | 0 | 0 | 0 | 0 | 0 | 188 | 70 |
| 440 | 183 | 20 | 0 | 0 | 0 | 17 | 0 | 0 | 1 | 0 | 0 | 0 | 1 | 0 | 0 | 0 | 0 | 0 | 22 | 18 |
| 441 | 183 | 1 | 0 | 0 | 0 | 252 | 0 | 0 | 6 | 0 | 0 | 0 | 0 | 0 | 0 | 0 | 3 | 0 | 227 | 39 |
| 442 | 183 | 0 | 2 | 0 | 0 | 271 | 0 | 0 | 4 | 0 | 0 | 0 | 0 | 0 | 0 | 0 | 2 | 0 | 230 | 98 |
| 443 | 183 | 0 | 1 | 0 | 0 | 241 | 0 | 0 | 1 | 0 | 0 | 0 | 0 | 0 | 0 | 0 | 1 | 0 | 143 | 117 |
| 444 | 182 | 0 | 1 | 0 | 0 | 263 | 0 | 0 | 5 | 0 | 0 | 0 | 1 | 0 | 0 | 0 | 0 | 0 | 32 | 30 |
| 445 | 182 | 0 | 2 | 1 | 0 | 323 | 0 | 0 | 2 | 0 | 0 | 0 | 0 | 0 | 0 | 0 | 0 | 0 | 62 | 14 |
| 446 | 182 | 1 | 0 | 0 | 0 | 240 | 0 | 0 | 2 | 0 | 0 | 0 | 0 | 0 | 0 | 0 | 0 | 0 | 81 | 46 |
| 447 | 181 | 20 | 0 | 0 | 0 | 0 | 0 | 0 | 0 | 0 | 0 | 0 | 0 | 0 | 0 | 0 | 0 | 0 | 78 | 35 |
| 448 | 181 | 5 | 3 | 0 | 0 | 225 | 0 | 0 | 3 | 0 | 0 | 0 | 1 | 0 | 0 | 0 | 0 | 0 | 786 | 412 |
| 449 | 181 | 0 | 0 | 0 | 0 | 249 | 0 | 0 | 4 | 0 | 0 | 0 | 0 | 0 | 0 | 0 | 0 | 0 | 69 | 5 |
| 450 | 181 | 2 | 5 | 0 | 0 | 228 | 0 | 0 | 0 | 0 | 0 | 0 | 0 | 0 | 0 | 0 | 0 | 0 | 23 | 32 |
| 451 | 181 | 9 | 4 | 0 | 0 | 149 | 0 | 0 | 4 | 0 | 0 | 0 | 0 | 0 | 2 | 0 | 0 | 0 | 80 | 74 |
| 452 | 180 | 0 | 0 | 0 | 0 | 261 | 0 | 0 | 6 | 0 | 0 | 0 | 0 | 0 | 0 | 0 | 0 | 0 | 1 | 0 |
| 453 | 180 | 0 | 1 | 0 | 0 | 270 | 0 | 0 | 2 | 0 | 0 | 0 | 0 | 0 | 0 | 0 | 0 | 0 | 46 | 40 |
| 454 | 180 | 14 | 4 | 0 | 0 | 70 | 0 | 0 | 1 | 0 | 0 | 0 | 3 | 0 | 0 | 0 | 1 | 0 | 194 | 58 |
| 455 | 179 | 20 | 0 | 4 | 0 | 79 | 0 | 0 | 0 | 0 | 0 | 0 | 0 | 0 | 0 | 0 | 0 | 0 | 427 | 148 |
| 456 | 178 | 0 | 0 | 0 | 0 | 534 | 0 | 0 | 1 | 0 | 0 | 0 | 0 | 0 | 0 | 0 | 0 | 0 | 42 | 13 |
| 457 | 178 | 0 | 2 | 0 | 0 | 311 | 0 | 0 | 5 | 0 | 0 | 0 | 0 | 0 | 0 | 0 | 0 | 0 | 183 | 47 |
| 458 | 178 | 17 | 2 | 0 | 0 | 13 | 0 | 0 | 0 | 0 | 0 | 0 | 0 | 0 | 0 | 0 | 0 | 0 | 42 | 12 |
| 459 | 177 | 0 | 0 | 0 | 0 | 267 | 0 | 0 | 4 | 0 | 0 | 0 | 0 | 0 | 0 | 0 | 0 | 0 | 2 | 0 |
| 460 | 177 | 9 | 1 | 0 | 0 | 246 | 0 | 0 | 0 | 0 | 0 | 0 | 0 | 0 | 0 | 0 | 0 | 0 | 5 | 12 |
| 461 | 177 | 8 | 0 | 0 | 0 | 178 | 0 | 0 | 1 | 0 | 0 | 0 | 0 | 0 | 0 | 0 | 0 | 0 | 73 | 29 |
| 462 | 177 | 3 | 1 | 0 | 0 | 213 | 0 | 0 | 6 | 0 | 0 | 0 | 3 | 0 | 0 | 0 | 0 | 0 | 108 | 70 |
| 463 | 177 | 7 | 0 | 0 | 0 | 151 | 0 | 0 | 9 | 1 | 0 | 0 | 1 | 0 | 0 | 0 | 0 | 0 | 18 | 5 |
| 464 | 177 | 6 | 1 | 1 | 0 | 94 | 0 | 0 | 139 | 0 | 3 | 0 | 1 | 0 | 0 | 0 | 0 | 0 | 236 | 136 |
| 465 | 176 | 17 | 0 | 0 | 0 | 21 | 0 | 0 | 0 | 0 | 0 | 0 | 0 | 0 | 0 | 0 | 0 | 0 | 20 | 3 |
| 466 | 176 | 9 | 6 | 1 | 0 | 78 | 0 | 0 | 19 | 0 | 1 | 0 | 0 | 0 | 0 | 0 | 0 | 0 | 515 | 648 |
| 467 | 176 | 4 | 2 | 0 | 0 | 164 | 0 | 0 | 0 | 0 | 0 | 0 | 0 | 0 | 0 | 0 | 0 | 0 | 32 | 20 |
| 468 | 176 | 19 | 0 | 0 | 0 | 3 | 0 | 0 | 1 | 0 | 0 | 0 | 0 | 0 | 0 | 0 | 0 | 0 | 16 | 18 |
| 469 | 176 | 0 | 2 | 2 | 0 | 239 | 0 | 0 | 11 | 0 | 1 | 0 | 0 | 0 | 0 | 0 | 0 | 0 | 511 | 412 |
| 470 | 175 | 0 | 2 | 0 | 0 | 329 | 0 | 0 | 1 | 0 | 0 | 0 | 0 | 0 | 0 | 0 | 0 | 0 | 35 | 19 |
| 471 | 175 | 14 | 0 | 0 | 0 | 107 | 0 | 0 | 0 | 1 | 0 | 0 | 0 | 0 | 0 | 0 | 0 | 0 | 25 | 6 |
| 472 | 174 | 17 | 1 | 0 | 0 | 23 | 0 | 0 | 2 | 0 | 0 | 0 | 0 | 0 | 0 | 0 | 0 | 0 | 58 | 17 |
| 473 | 174 | 0 | 1 | 0 | 0 | 276 | 0 | 0 | 2 | 0 | 0 | 0 | 0 | 0 | 0 | 0 | 0 | 0 | 83 | 27 |
| 474 | 173 | 0 | 7 | 7 | 0 | 243 | 0 | 0 | 0 | 0 | 0 | 0 | 0 | 0 | 0 | 0 | 0 | 0 | 284 | 147 |
| 475 | 173 | 9 | 3 | 0 | 0 | 146 | 0 | 0 | 2 | 0 | 0 | 0 | 0 | 0 | 0 | 0 | 0 | 0 | 13 | 9 |
| 476 | 172 | 6 | 2 | 0 | 0 | 151 | 0 | 0 | 15 | 1 | 1 | 0 | 0 | 0 | 0 | 0 | 0 | 0 | 875 | 774 |
| 477 | 172 | 8 | 1 | 0 | 0 | 215 | 0 | 0 | 0 | 0 | 0 | 0 | 0 | 0 | 0 | 0 | 0 | 0 | 16 | 11 |
| 478 | 172 | 0 | 0 | 0 | 0 | 245 | 0 | 0 | 4 | 0 | 0 | 0 | 0 | 0 | 0 | 0 | 0 | 0 | 127 | 73 |
| 479 | 171 | 0 | 0 | 0 | 1 | 243 | 0 | 0 | 3 | 0 | 0 | 0 | 0 | 0 | 0 | 0 | 0 | 0 | 186 | 53 |
| 480 | 171 | 9 | 1 | 4 | 18 | 76 | 0 | 0 | 10 | 0 | 0 | 0 | 0 | 0 | 0 | 0 | 0 | 0 | 287 | 63 |
| 481 | 170 | 1 | 3 | 0 | 0 | 253 | 0 | 0 | 2 | 0 | 0 | 0 | 0 | 0 | 0 | 0 | 0 | 0 | 221 | 156 |
| 482 | 170 | 26 | 0 | 0 | 0 | 1 | 0 | 0 | 0 | 0 | 0 | 0 | 0 | 0 | 0 | 0 | 0 | 0 | 46 | 17 |
| 483 | 170 | 7 | 0 | 0 | 0 | 296 | 0 | 0 | 1 | 0 | 0 | 0 | 0 | 0 | 0 | 0 | 0 | 0 | 57 | 43 |
| 484 | 170 | 6 | 1 | 0 | 0 | 207 | 0 | 0 | 0 | 0 | 0 | 0 | 0 | 0 | 0 | 0 | 0 | 0 | 13 | 4 |
| 485 | 170 | 0 | 0 | 0 | 0 | 282 | 0 | 0 | 8 | 0 | 0 | 0 | 0 | 0 | 0 | 0 | 0 | 0 | 257 | 119 |
| 486 | 169 | 22 | 1 | 0 | 0 | 6 | 0 | 0 | 2 | 0 | 0 | 0 | 0 | 0 | 0 | 0 | 0 | 0 | 75 | 54 |
| 487 | 168 | 4 | 2 | 0 | 0 | 222 | 0 | 0 | 1 | 0 | 0 | 0 | 0 | 0 | 0 | 0 | 0 | 0 | 24 | 30 |
| 488 | 168 | 0 | 0 | 0 | 0 | 316 | 0 | 0 | 3 | 0 | 0 | 0 | 0 | 0 | 0 | 0 | 0 | 0 | 328 | 367 |
| 489 | 167 | 12 | 1 | 0 | 0 | 110 | 0 | 0 | 9 | 0 | 1 | 0 | 0 | 0 | 0 | 0 | 2 | 0 | 294 | 191 |
| 490 | 167 | 16 | 0 | 0 | 0 | 66 | 0 | 0 | 9 | 0 | 0 | 0 | 0 | 0 | 0 | 0 | 0 | 0 | 131 | 74 |
| 491 | 166 | 0 | 2 | 0 | 0 | 272 | 0 | 0 | 3 | 0 | 0 | 0 | 0 | 0 | 1 | 0 | 0 | 0 | 208 | 126 |
| 492 | 166 | 14 | 0 | 0 | 0 | 83 | 0 | 0 | 2 | 0 | 0 | 0 | 0 | 0 | 0 | 0 | 0 | 0 | 64 | 37 |
| 493 | 166 | 0 | 0 | 0 | 0 | 235 | 0 | 0 | 0 | 0 | 0 | 0 | 0 | 0 | 1 | 0 | 0 | 0 | 52 | 31 |
| 494 | 166 | 27 | 0 | 0 | 0 | 16 | 0 | 0 | 1 | 0 | 0 | 0 | 0 | 0 | 2 | 0 | 0 | 0 | 89 | 66 |
| 495 | 166 | 0 | 0 | 0 | 0 | 224 | 0 | 0 | 6 | 0 | 0 | 0 | 0 | 0 | 0 | 0 | 0 | 0 | 5 | 3 |
| 496 | 165 | 0 | 0 | 0 | 0 | 198 | 0 | 0 | 0 | 0 | 0 | 0 | 0 | 0 | 0 | 0 | 0 | 0 | 33 | 3 |
| 497 | 165 | 0 | 0 | 0 | 0 | 235 | 0 | 0 | 0 | 0 | 0 | 0 | 0 | 0 | 0 | 0 | 0 | 0 | 26 | 3 |
| 498 | 164 | 1 | 2 | 0 | 0 | 178 | 0 | 0 | 3 | 0 | 0 | 0 | 0 | 0 | 0 | 0 | 0 | 0 | 169 | 35 |
| 499 | 164 | 1 | 1 | 0 | 0 | 266 | 0 | 0 | 5 | 0 | 0 | 0 | 1 | 0 | 0 | 0 | 0 | 0 | 67 | 21 |
| 500 | 164 | 10 | 1 | 0 | 0 | 65 | 0 | 1 | 1 | 0 | 0 | 0 | 0 | 0 | 0 | 0 | 0 | 0 | 101 | 38 |
|  | | | | | | | | | | | | | | | | | | | | |
